# Transcriptomic and phenotype analysis revealed the role of *rpoS* in stress resistance and virulence of a novel ST3355 ESBL-producing hypervirulent *Klebsiella pneumoniae* isolate

**DOI:** 10.3389/fcimb.2023.1259472

**Published:** 2023-10-23

**Authors:** Yi Zhou, Yue Cheng, Tianyou Ma, Jun Wang, Shaoru Li, Jingdan Wang, Lei Han, Xinyao Hou, Xinxin Ma, Sijin Jiang, Pu Li, Jia Lv, Bei Han, Rong Da

**Affiliations:** ^1^ School of Public Health, Health Science Center, Xi’an Jiaotong University, Xi’an, China; ^2^ Department of Microbiology Laboratory, Tongchuan Center for Disease Control and Prevention, Tongchuan, Shaanxi, China; ^3^ School of Basic Medicine, Health Science Center, Xi’an Jiaotong University, Xi’an, China; ^4^ Department of Clinical Laboratory, The First Affiliated Hospital of Xi’an Jiaotong University, Xi’an, China

**Keywords:** hypervirulent *Klebsiella pneumoniae*, ESBLs, pathogenomic, rpoS, virulence, environmental stress and disinfectants tolerance, T4SS

## Abstract

**Introduction:**

An extended-spectrum beta-lactamase (ESBL)-hypervirulent *Klebsiella pneumoniae* (HvKP) strain HKE9 was isolated from the blood in an outpatient.

**Methods:**

The effect of the global regulatory factor RpoS on antimicrobial resistance, pathogenicity, and environmental adaptability was elucidated.

**Results:**

HKE9 is a novel ST3355 (K20/O2a) hypervirulent strain with a positive string test and resistant to cephems except cefotetan. It has a genome size of 5.6M, including two plasmids. CTX-M-15 was found in plasmid 2, and only *ompk37* was found in the chromosome. HKE9 could produce bacterial siderophores, and genes of enterobactin, yersiniabactin, aerobactin, and salmochelin have been retrieved in the genome. As a global regulatory factor, knockout of *rpoS* did not change antimicrobial resistance or hemolytic phenotype while increasing the virulence to *Galleria mellonella* larvae and showing higher viscosity. Moreover, *rpoS* knockout can increase bacterial competitiveness and cell adhesion ability. Interestingly, HKE9-M-rpoS decreased resistance to acidic pH, high osmotic pressure, heat shock, and ultraviolet and became sensitive to disinfectants (H_2_O_2_, alcohol, and sodium hypochlorite). Although there were 13 Type 6 secretion system (T6SS) core genes divided into two segments with *tle1* between segments in the chromosome, transcriptomic analysis showed that *rpoS* negatively regulated T4SS located on plasmid 2, type 1, and type 3 fimbriae and positively regulate genes responsible for acidic response, hyperosmotic pressure, heat shock, oxidative stress, alcohol and hypochlorous acid metabolism, and quorum sensing.

**Discussion:**

Here, this novel ST3355 ESBL-HvKP strain HKE9 may spread via various clonal types. The important regulation effect of *rpoS* is the enhanced tolerance and resistance to environmental stress and disinfectants, which may be at the cost of reducing virulence and regulated by T4SS.

## 1 Introduction

*Klebsiella pneumoniae* is one of the most important threats to human health because of its ever-increasing drug resistance to almost all known antibiotics for the treatment of Gram-negative bacilli. Particularly, the carbapenem-resistant strains of *K. pneumoniae* (CRKP) have extremely high lethality, and the mortality of bloodstream infection can reach approximately 50% (Munoz-Price et al., 2013), which might result in a clinical anti-infective treatment dilemma. Since the 1980s, a new hypervirulent strain of *K. pneumoniae* [extended-spectrum beta-lactamases (ESBLs)] together with the characteristics of hypermucoviscosity has gradually appeared in clinical infections from Southeast Asia, which was also recently reported in Western countries (Babouee Flury et al., 2017; Lee et al., 2017; Scapaticci et al., 2017) and even affected non-human primates (NHPs) (Anzai et al., 2017). In a clinical study, 22.8% (84/369) of *K. pneumoniae* strains were hypermucoviscous isolates resulting in invasive infections (Guo et al., 2017). Moreover, the emergence and widespread of HvKP strains often lead to fatal metastatic infections (Shon et al., 2013).

Most HvKP strains are sensitive to almost all commonly used antibiotics other than ampicillin in clinical application. However, there have been reports of drug-resistant HvKP strains in recent years, such as the ESBL-producing HvKP (ESBL-HvKP) and carbapenem-resistant HvKP (CR-HvKP) strains, which may become the next crisis in clinical infection treatments (Lee et al., 2017). Those strains have both resistance to antibiotics and virulence factors caused by invasive infections, which will expose patients to serious infection without effectively susceptible antimicrobials. Thus, it raised the importance of clarifying the precise mechanism of antimicrobial resistance and virulence factors in those multidrug-resistant HvKP isolates.

HvKP was initially isolated from liver abscesses caused by severe community-acquired infections and is now prevalent in bacteremia (Shankar et al., 2018). In this study, an ESBL-HvKP strain HKE9 with novel ST3355 was isolated from the blood of an outpatient of a tertiary hospital in northwest China. The strain was characterized as a hypermucoviscous *K. pneumoniae* (HmKP), and the whole genome was sequenced. The antimicrobial resistance and virulence factors were elucidated. RpoS, a global regulatory sigma factor, is important for bacterial survival under extreme conditions. Many enterobacteria and their ability to survive in a changing environment could be an essential step for their virulence (Shankar et al., 2018). In order to know if RpoS regulates gene expression at multiple levels related to multidrug resistance (MDR) and virulence of HvKP/HmKP, the *rpoS* knockout mutant was generated in HKE9. It was found that *rpoS* could affect the phenotype and virulence of HKE9 in many ways, including viscosity, cell adhesion, resistance to environmental stress, iron ion transport, and quorum sensing. The *rpoS* can also affect the tolerance of ESBL-HvKP strain HKE9 to various disinfectants and bacterial competitiveness, which may be related to T4SS. Transcriptomic analysis was used to verify how RpoS participates in those metabolic pathways.

## 2 Materials and methods

### 2.1 Bacterial strains and cell culture

The strains, plasmids, and primers used in this study are shown in **Table S1**. *Acinetobacter baumannii* 798129 is a clinical isolate; *K. pneumoniae* ATCC 700603, *Escherichia coli* DH5α, *E. coli* ATCC 25922, and *A. baumannii* 798129 were all stored in our laboratory. The strains were inoculated in 5 mL of LB medium and incubated at 37°C for 12–18 h aerobically. Antibiotics were used as required: ampicillin (Amp) 100 μg/mL, tetracycline (Tet) 15 μg/mL, chloramphenicol (Chl) 100 μg/mL, and kanamycin (Kan) 50 μg/mL.

Human lung adenocarcinoma cells A549 were maintained in Roswell Park Memorial Institute (RPMI) 1640 medium supplemented with 10% fetal bovine serum and 1% non-essential amino acid (Gibco/BRL, Grand Island, NY, USA) and incubated at 37°C in 5% CO_2_–95% air atmosphere. Cells were seeded in a 24-well plate (2 × 10^5^ cells/well) and incubated at 37°C for 24 h. After the cells reached confluency, they were used in the following experiments.

### 2.2 Isolation and characterization of ESBL-HvKP strain HKE9

A string test-positive *K. pneumoniae* strain (HKE9) was isolated from the blood of an outpatient in the clinical laboratory of the First Affiliated Hospital of Xi’an Jiaotong University, Xi’an, Shaanxi Province, China. The HKE9 was resistant to most cephems and determined as an ESBL-producing strain. This study was approved by the ethics committee of the First Affiliated Hospital of Xi’an Jiaotong University.

An antibiotic susceptibility test was performed using a VITEK 2 Compact device (bioMérieux Inc., Marcy-l’Étoile, France) with VITEK 2 AST-GN 09. The interpretation of susceptibility data followed the CLSI M100 31st Edition. The HKE9 strain was tested for ESBL production by disk diffusion test, and *K. pneumoniae* ATCC 700603 (positive) and *E. coli* ATCC 25922 (negative) were used as quality control and performed as required in the manufacturer’s handbook.

### 2.3 Mucoviscosity test

The string test was performed as previously described (Pan et al., 2011). Briefly, the tested strains were inoculated on blood agar plates and incubated at 37°C overnight. A positive string test was defined by the formation of viscous filament ≥5 mm in length when a standard bacteriologic loop was used to stretch the colony. Low-speed centrifugation was also performed in liquid culture in semi-quantitation of mucoviscosity. Briefly, equal numbers of bacteria cells in 1.1 mL of lysogeny broth medium were centrifuged at 2,000 *g* for 5 min. Then, the optical density at 600 nm (OD_600_) of the 1-mL supernatant was measured. The test was repeated three times.

### 2.4 Bacterial siderophore detection

The siderophore (aerobactin, salmochelin, enterobactin, and yersiniabactin) biosynthesis in *K. pneumoniae* and iron acquisition from the host are essential for virulence (Hsieh et al., 2008). The HvKP strains have a 6- to 10-fold increased siderophore activity as compared with cKP strains. The siderophore production of the HKE9 strain was detected qualitatively by using a CAS (Chrome Azurol Sulfonate) agar test (Schwyn and Neilands, 1987). The orange halos around the *K. pneumoniae* colonies on blue CAS agar are positive siderophore excretion. For quantitative detection, a UV–visible spectrophotometric assay was performed (Pu et al., 2023). The HKE9 strain culture of 1.5 × 10^9^ CFU/mL was sub-cultured into peptone liquid medium (10 g/L of peptone, 5 g/L of NaCl, pH 7.8) and incubated at 37°C for 18 h. The culture was centrifuged at 1,091 *g* for 15 min, and the supernatant was transferred and mixed with CAS solution for 15 min. OD_630_ was measured finally. The sterile medium mixed with CAS solution served as the blank control. The *K. pneumoniae* strain ATCC 700603 was used as the negative control. The percentage of secreted siderophores units was calculated as [(Ar − As)/Ar] × 100%, where Ar is OD_630_ of the negative control, and As is OD_630_ of the tested sample.

### 2.5 Complete genome sequence analysis of HKE9

The genomic DNA of HKE9 was extracted by kit (Applied Biosystems^®^ 4413021, Foster City, CA, USA). The library was constructed and sequenced on an Oxford Nanopore Technologies (ONT) sequencer at Biomarker Technologies, Beijing, China. The filtered reads were assembled by Canu v1.5, and then circlator v1.5.5 was taken to cyclize the assembled genome. Coding gene prediction was performed by Prodigal v2.6.3. The GenBlastA v1.0.4 was used to scan the whole genomes after masking predicted functional genes. Putative candidates were then analyzed by searching for non-mature mutations and frame-shift mutations using GeneWise v2.2.0. For functional annotation, the predicted proteins were blast (e-value: 1e−5) against Nr, Swiss-Prot, TrEMBL, KEGG, EggNOG, and Blast2GO and used for Gene Ontology (GO) annotation. Furthermore, the pathogenicity and drug resistance can be researched by blast against CAZy (Carbohydrate-Active enZYmes Database, http://www.cazy.org/), TCDB (Transporter Classification Database, https://tcdb.org/), CARD (the Comprehensive Antibiotic Resistance Database, https://card.mcmaster.ca/), PHI (Pathogen Host Interactions, http://www.phi-base.org/), and VFDB (Virulence Factor Database, http://www.mgc.ac.cn/VFs/main.htm). Secretory proteins were detected by SignalP after transmembrane proteins were filtered by TMHMM v2.0c. With the use of the assembled and predicted genome information, such as tRNA, rRNA, repeat sequences, GC content, and gene function information, the software Circos v0.66 (Krzywinski et al., 2009) was used to draw a genome circle map. For the plasmid sequences, TAfinder was applied to identify Type II toxin–antitoxin loci (Xie et al., 2018) (https://bioinfo-mml.sjtu.edu.cn/TAfinder/TAfinder.php); MobileElementFinder was used to detect the mobile genetic elements associated with antibiotic resistance in HKE9 (Johansson et al., 2021). The bacterial integrative and conjugative elements were analyzed in ICEfinder with ICEberg2.0 (Liu et al., 2019) (https://bioinfo-mml.sjtu.edu.cn/ICEfinder/index.php).

### 2.6 Multi-locus sequence typing

The phylogenetic distance of HKE9 was analyzed by multi-locus sequence typing (MLST) (Guo et al., 2015). The sequence types (STs) were assigned through the *Klebsiella* database (https://pubmlst.org/). The complete genome sequence was also submitted to *Klebsiella* Sequence Typing Home Page (https://bigsdb.pasteur.fr/klebsiella/) to determine the MLST type. The MLST locus data were submitted to compare with MLST data of *K. pneumoniae* using global optimal eBURST (http://www.phyloviz.net/goeburst/). Kaptive 2.0 was used for identifying and typing capsule (K) and outer LPS (O) loci from whole genome sequence data (https://github.com/katholt/kaptive) (Wick et al., 2018). Kleborate was used to identify the integrative conjugative element (ICEKp) (Lam et al., 2018).

### 2.7 Determination of antibiotic resistance mechanisms

For the analysis of antibiotic resistance genes and mutation in the bacterial genome, the complete genome sequence of HKE9 was analyzed using Resistance Gene Identifier (RGI, https://card.mcmaster.ca/analyze/rgi) software for prediction of resistome based on homology and SNP models in CARD.

### 2.8 Determination of virulence factors

For the analysis of virulence factors in the bacterial genome, the complete genome sequence of HKE9 was blast against VFDB. Furthermore, we compare pathogenomics with four representative *K. pneumoniae* strains, including *K. pneumoniae* subsp. *pneumoniae* 1084 (chromosome NC_018522), *K. pneumoniae* subsp. *pneumoniae* HS11286 (chromosome NC_016845), *K. pneumoniae* subsp. *pneumoniae* MGH78578 (chromosome NC_009648), and *K. pneumoniae* subsp. *pneumoniae* NTUH-K2044 (chromosome NC_012731). Strain NTUH-K2044 is an ST23 HvKP; 1084 is string test negative with capsule typing of K1 and causes bloodstream infection; HS11286 is an ST11 HvKP with carbapenem resistance; MGH78578 is usually used as the reference *K. pneumoniae* strain. HKE9 is a new ST3355 strain with high mucoviscosity with MDR, and it is compared with other four representative *K. pneumoniae* strains to reveal its own characteristics.

### 2.9 Mechanism exploration of global regulatory factor *rpoS* in HKE9

#### 2.9.1 Phylogenetic analysis of *rpoS*

Homology analysis was conducted between *rpoS* sequences of strain HKE9 and *rpoS* sequences of the other 23 strains. For homologous genes, MEGA7 software was used for multi-sequence alignment to construct the evolutionary tree.

#### 2.9.2 Construction of mutant strain HKE9-M-*rpoS* and complement strain HKE9-C-M-*rpoS*

The *rpoS* knockout mutant HKE9-M-*rpoS* was constructed using the lambda Red recombination method (Datsenko and Wanner, 2000). A two-step polymerase chain reaction was used to amplify the kanamycin resistance gene fragment with a flippase recognition site and the gene fragment of approximately 150 bp upstream and downstream of *rpoS* using pKD4 and HKE9 as templates, respectively, to obtain the *rpoS* gene mutation cassette. Primers are shown in **Table S1**. To construct *rpoS* deletion mutants, plasmid pKD46 was transformed into wild-type HKE9 with arabinose induction. The pCP20 was transformed into the recombinant mutant strain to remove the kanamycin resistance gene. Finally, *rpoS* mutant HKE9-M-*rpoS* was obtained.

The complete *rpoS* gene coding region was amplified by PCR and cloned into plasmid pBAD33 to obtain pBAD33-*rpoS*. The pBAD33-*rpoS* was transformed into HKE9-M-*rpoS* by electroporation. Complementation strain HKE9-C-M-*rpoS* was selected on LB agar supplemented with 100 µg/mL of chloramphenicol.

#### 2.9.3 Hemolytic activity assay

Hemolytic activity of *K. pneumoniae* strains was evaluated (Maturana et al., 2017). HKE9, HKE9-M-*rpoS*, and HKE9-C-M-*rpoS* were cultured in an LB liquid medium overnight. Then, they were subjected to the streak plate method on Columbia agar supplemented with 5% (v/v) sheep blood and cultured at 37°C for 24 h to observe the hemolytic cycle of colonies on plates. Hemolytic activity can be divided into α-hemolysis, β-hemolysis, and γ-hemolysis.

#### 2.9.4 Environmental stress tolerance ability measurement

HKE9, HKE9-M-*rpoS*, and HKE9-C-M-*rpoS* were grown overnight in LB at 37°C. The overnight bacterial culture (1 mL) was centrifuged, and the collected cells were washed and resuspended into phosphate-buffered saline (PBS) with OD_600_ of 0.2. Different pH values (2.0, 3.0, 4.0, 5.0, and 6.0), NaCl solutions (2.5%, 5.0%, 7.5%, 10%, 12.5%), heat shock (42°C, 50°C), and UV were used as the treatment. The treatment time of pH, NaCl, and high temperature was 1 h, and the UV treatment was 10 min, 20 min, and 30 min. Sterilized PBS (pH 7.0) was used as the negative control. The treated bacterial solution was serially diluted and spread on LB plates and cultured at 37°C for 24 h, and colonies were counted to calculate the bacterial survival rate. Three replicates were set up for each experiment (Chiang et al., 2011).


Survival rate(%)=[CFU of treated bacteria/CFU of untreated bacteria]×100%.

#### 2.9.5 Disinfectant stress tolerance ability measurement

HKE9, HKE9-M-*rpoS*, and HKE9-C-M-*rpoS* were cultured overnight and centrifuged, and then cell pellets were resuspended to OD_600 _= 0.2. The 110-µL bacterial solution was spread on an LB plate. Then, 6-mm sterilized disks were placed on an LB plate, and different concentrations of H_2_O_2_ (5%, 10%, 20%, and 30%), alcohol (55%, 65%, and 75%), and sodium hypochlorite disinfectant (1:0, 1:5, 1:10, 1:50, and 1:100) were dropped. Liquid with a volume of 15 μL in a different concentration or dilution ratio was added to each disk. Plates were incubated overnight at 37°C, and the inhibition zone was measured. Three replicates were set up for each experiment (Zhang et al., 2023).

#### 2.9.6 Bacterial virulence/toxicity assay

The *Galleria mellonella* larvae infection model was used for bacterial virulence assay (Storey et al., 2020). The larvae weighing approximately 300 mg (Tianjin Huiyude Biotech Company, Tianjin, China) were maintained on woodchips in the dark at 15°C before use. The overnight cultures of HKE9, HKE9-M-*rpoS*, and HKE9-C-M-*rpoS* suspension were adjusted into concentrations of 1 × 10^3^ CFU/mL, 1 × 10^4^ CFU/mL, 1 × 10^5^ CFU/mL, 1 × 10^6^ CFU/mL, 1 × 10^7^ CFU/mL, and 1 × 10^8^ CFU/mL. Ten randomly selected larvae were used in each group. Each larva was inoculated with the bacterial suspension via the rear left proleg using a 10-μL Hamilton syringe. The LB medium was injected into larvae and set as the negative control. The treated *G. mellonella* larvae were incubated at a 37°C incubator for 72 h, and the survival rate of the larvae was recorded every 12 h.

#### 2.9.7 Bacterial competition assay

Predator and prey strains were grown to the mid-exponential stage, collected, and resuspended in PBS to OD_600_ of 1.0. In self-competition, predator HKE9 and predator HKE9-C-M-*rpoS* were mixed with prey HKE9-M-*rpoS* in a ratio of 1:1. In interspecies or intraspecies bacterial competition, HKE9, HKE9-M-*rpoS*, and HKE9-C-M-*rpoS* are predators. *K. pneumoniae* ATCC 700603, *E. coli* DH5α, and *A. baumannii* 798129 are prey strains. Then, predators and preys were mixed in a ratio of 10:1. The 25 μL of mixed bacterial cultures was incubated on preheated LB agar at 37°C for 5 h. Bacterial spots were harvested, the recovered cells were plated on a selective medium containing the corresponding antibiotics, and the viability of prey cells per milliliter was measured (Hsieh et al., 2019).


Survival rate(%)=(CFU of competitive treated bacteria/CFU of control bacteria)×100%.

#### 2.9.8 Cell adhesion assay

Adhesion assays were performed with A549 cells in a 24-well plate (2.5 × 10^5^ cells per well) and were prewashed with sterilized PBS (Hsieh et al., 2016). HKE9, HKE9-M-*rpoS*, and HKE9-C-M-*rpoS* (mid-log phase, OD_600 _= 0.4–0.6) suspending in fetal bovine serum (FBS)-free RPMI 1640 medium in 8 × 10^7^ CFU/mL were added to each well at a multiplicity of infection (MOI) of 10 bacteria/cell and incubated for 30 min at 37°C in a humid environment of 5% CO_2_ atmosphere. After incubation, cell wells were washed to remove the free bacteria, and 0.25% trypsin was added to A549 cells and digested for 3–5 min to lyse the adherent *K. pneumoniae* cells. Recovered bacteria were quantified by plate counting. All experiments were performed in triplicate.


$$\begin{array}{l} \text{Relative }\mathrm{adhesion}\;\mathrm{rate}(\%)=[\mathrm{adhered}\;\mathrm{HKE9}-\text{M}-rpoS\;or\;\mathrm{HKE9}-\text{C}-\text{M}-rpoS\\ (\mathrm{CFU}/\mathrm{mL})/\mathrm{adhered}\;\mathrm{HKE9}\;(\mathrm{CFU}/\mathrm{mL})]\times 100\%. \end{array}$$

#### 2.9.9 Transcriptome sequencing

Fresh cultures of HKE9, HKE9-M-*rpoS*, and HKE9-C-M-*rpoS* were grown to OD_600_ of 0.6. Total RNA was extracted using RNeasy minikit (Qiagen, Gaithersburg, DE, USA). RNA quality was monitored. RNA purity was checked using the NanoPhotometer^®^ spectrophotometer (Implen, Westlake Village, CA, USA). RNA integrity was measured using Bioanalyzer 2100 (Agilent, Santa Clara, CA, USA). A total amount of 3 μg of RNA per sample was used as input material for RNA sample preparations. Sequencing libraries were generated using NEBNext^®^ UltraTM RNA Library Prep Kit for Illumina^®^ (NEB, Ipswich, MA, USA), and then transcriptome sequencing was performed at Novaseq 6000 in Gene Denovo Co., Ltd. (Guangzhou, China). Quality-trimmed reads were mapped to HKE9 using Bowtie2 v2.2.8 (Langmead and Salzberg, 2012), and reads mapped to ribosome RNA were removed. Retained reads were aligned with the reference genome to identify known genes and calculated gene expression by RSEM (Li and Dewey, 2011). To evaluate reproducibility between samples, the correlation coefficient among replicas was calculated. Values closer to one indicated better reproducibility. Principal component analysis (PCA) was performed with the R package gmodels (http://www.r-project.org) to reveal the relationship between samples. The gene expression level was further normalized by using the Fragments per Kilobase of transcript per Million mapped reads (FPKM) method to eliminate the influence of different gene lengths and amount of sequencing data on the calculation of gene expression. The edgeR package (http://www.r-project.org/) was used to identify differentially expressed genes (DEGs) across samples with fold changes ≥2 and a false discovery rate (FDR)-adjusted *p* (*q*)< 0.05. DEGs were then subjected to an enrichment analysis of GO functions and Kyoto Encyclopedia of Genes and Genomes (KEGG) pathways, and *q* values<0.05 were used as the threshold. The raw sequencing data were then submitted to the National Center for Biotechnology Information (NCBI) Gene Expression Omnibus (GEO) database (GSE224709).

#### 2.9.10 Validation of RNA-Seq by qRT-PCR

To validate the transcriptomic analysis of the mRNA sequencing data, qRT-PCR was used to quantify gene expression levels of HKE9, HKE9-M-*rpoS*, and HKE9-C-M-*rpoS*. RNA was extracted as described before. The total RNA samples (5 μg each) were transcribed into cDNA, the RT-PCR program was 42°C for 1 h and 72°C for 10 min, and the PCR product was stored at −20°C for quantitative real-time PCR. 16S rDNA served as a reference gene to quantify the expression of all genes. The primers for the selected genes (10 DEGs of *sufC*, *ariR*, *virB5*, *cbpA*, *virB10*, *mrkC*, *virB1*, *katE*, *osmY*, and *fimC*), the amplified fragment length, and the annealing temperature are all listed in **Table S1**. The reaction mixture consisted of SYBR Green I master mix (Roche, Basel, Switzerland), 0.25 µM of primer, and 1 µL of cDNA. To determine the expression of genes, the relative expression was analyzed using the 2^−ΔΔCt^ method, with 16S rDNA as the internal reference gene. Pearson’s correlation coefficient was used to analyze the correlation between RNA-Seq and qRT-PCR data.

### 2.10 Statistical analysis

All experimental data were presented in triplicate. The means ± SEM was used for all types of measures. ANOVA and Dunnett’s t-test were used to compare the differences between groups. *p<* 0.01 was considered a significant difference. Statistical analysis of the data was performed using the software SPSS V20.0 (IBM Inc., Chicago, IL, USA), and GraphPad Prism 9 was applied for graphical plotting and analysis.

## 3 Results

### 3.1 Mucoviscosity of ESBL-HvKP strain HKE9

**Figure 1:**
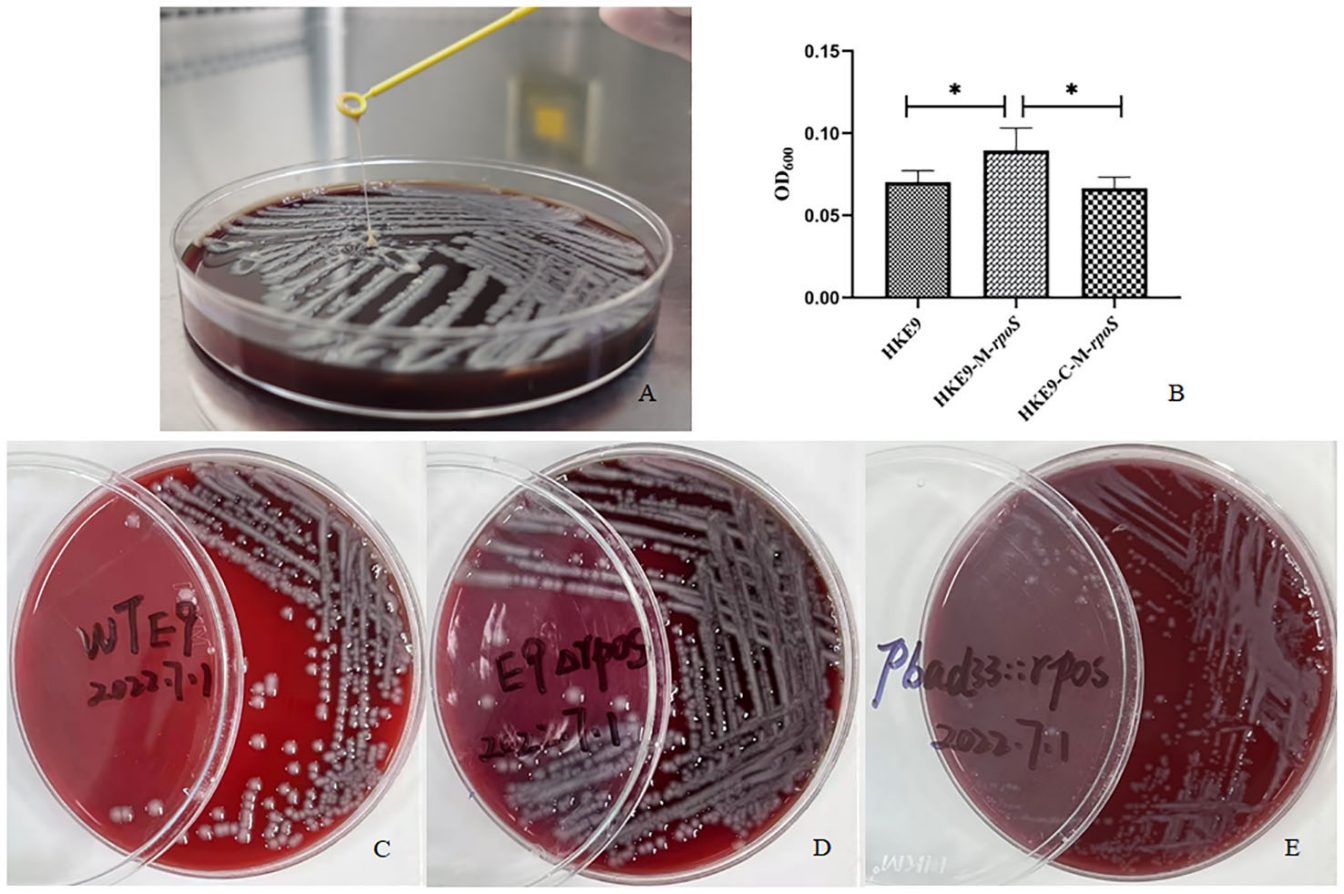
Mucoviscosity qualitative measurement by string test **(A)** and semi-quantitative measurement by low-speed centrifugation **(B)** in strains HKE9, HKE9-M*-rpoS*, and HKE9-C-M*-rpoS*. The hemolytic phenotype of HKE9 **(C)**, HKE9-M-*rpoS* **(D)**, and HKE9-C-M-*rpoS* **(E)**. *,P<0.05.

HKE9 was isolated in a blood sample from an outpatient. Positive mucoviscosity result was verified by string test with the formation of a 32-mm viscous filament in length when stretching the colony (**Figure 1A**). Furthermore, the mucoviscosity was quantitatively determined through low-speed centrifugation of 2,000 *g*, and the OD_600_ was 0.07 ± 0.01 (**Figure 1B**).

### 3.2 General bacteriological characteristics of HKE9

**Figure 2:**
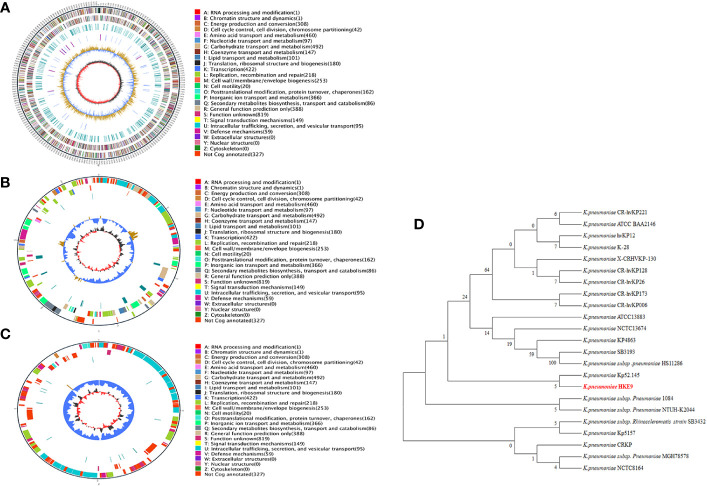
The circle diagram of the assembled genome of the *K. pneumonia* strain HKE9, genome **(A)**, plasmid 1 **(B)**, and plasmid 2 **(C)**. Genomic evolutionary analysis of *rpoS* **(D)**.

The genome of HKE9 is composed of a single circular chromosome (5,582,300 bp) with G+C content of 56.98%, one circular plasmid (161,792 bp), and one linear plasmid (99,544 bp) (**Figures 2A–C**). HKE9 contains 5,193 genes, of which 4,925 genes are on the chromosome, 155 genes are on the circular plasmid, and 113 genes are on the linear plasmid. The detailed genetic feature information is summarized in **Table 1** and **Table S2**. The genome sequence of HKE9 was deposited in GenBank (CP089768).

**Table 1:** The detailed information of the two plasmids carried by *K. pneumonia* strain HKE9.

	Location	Genes/locus	Name/synonyms
Antimicrobial resistance	Plasmid 2	GE005134 GE005135 GE005175	Cephalosporin
Plasmid mobilization	Plasmid 1	138653-139960	ISKpn2 (Insertion sequence)
	Plasmid 2	83997-85652	ISEc9/ISEcp1/ISEcp1B (Insertion sequence)
		46642-48297	ISEc9/ISEcp1/ISEcp1B (Insertion sequence)
		46641-85652	cn_39011_ISEc9 (Composite transposon)
Toxin–antitoxin (TA) system	Plasmid 1	55885-56361	Toxin (GNAT like_domain, COG2153)
		55610-55888	Antitoxin (RHH like_domain, CO4453)
		116603-117073	Toxin (COG5654 like_domain)
		116163-116606	Antitoxin (COG5642 like_domain)
Inc-group	Plasmid 1	144040-144599	IncFIB (K)
		38875-39103	IncFII
	Plasmid 2	94405-94728	Incl2

HKE9 was determined as a new ST3355, and seven alleles were submitted to the MLST system for alleles defined with *gapA* (1), *infB* (1), *mdh* (1), *pgi* (1), *phoE* (213), *rpoB* (185), and *tonB* (458). Compared with goeBURST, ST3355 is a single ST and does not belong to any clusters in the *K. pneumoniae* group. It showed the capsule (K) type of K20 and the outer LPS (O) type of O2a identified by Kaptive 2.0.

### 3.3 Antibiotic resistance phenotype and the related genes

The antibiotic susceptibility test result is shown in **Table 2**. HKE9 had an ESBL-producing phenotype and was resistant to most of the cephems except cefotetan. It was resistant to penicillins and monobactams, including piperacillin and aztreonam. For β-lactam combination agents, HKE9 was also resistant to ampicillin–sulbactam but susceptible to piperacillin–tazobactam. Moreover, HKE9 remains susceptible to aminoglycosides, fluoroquinolones, carbapenems, and folate pathway antagonists.

**Table 2:** The antimicrobial susceptibility test distribution of *K. pneumonia* strain HKE9.

		HKE9		HKE9-M-*rpoS*	
Antimicrobial class	Antimicrobial agent	MIC (µg/mL)	Interpretive categories	MIC (µg/mL)	Interpretive categories
Penicillins	Piperacillin	≥128	R	≥128	R
Cephems (parenteral)	Cefazolin	≥64	R	≥64	R
	Cefepime	≥64	R	≥64	R
	Cefotetan	≤4	S	≤4	S
	Ceftazidime	≥64	R	≥64	R
	Ceftriaxone	≥64	R	≥64	R
	Cefuroxime	≥64	R	≥64	R
Aminoglycosides	Gentamicin	≤1	S	≤1	S
	Tobramycin	≤1	S	≤1	S
	Amikacin	≤2	S	≤2	S
Fluoroquinolones	Ciprofloxacin	1	S	1	S
	Levofloxacin	1	S	1	S
Carbapenems	Imipenem	≤1	S	≤1	S
	Meropenem	≤0.25	S	≤0.25	S
β-Lactam combination agents	Ampicillin–sulbactam	≥32/16	R	≥32/16	R
	Piperacillin–tazobactam	≤4/4	S	≤4/4	S
Monobactams	Aztreonam	≥64	R	≥64	R
Folate pathway antagonists	Trimethoprim/sulfamethoxazole	≤20 (1/19)	S	≤20 (1/19)	S

MIC, minimum inhibitory concentration.

HKE9 harbored CTX-M-15 gene in the linear plasmid, and resistance genes to almost all antibiotic classes were annotated. There were four resistance mechanisms retrieved in the HKE9 chromosome, including antibiotic efflux, antibiotic inactivation, antibiotic target alteration, and reduced permeability. The above four mechanisms are all related to resistance to cephalosporin. The main outer membrane proteins of *K. pneumoniae* are OmpK 35, OmpK 36, and OmpK37. The mutation or deletion of genes encoding outer membrane proteins will render the antibiotics unable to penetrate the interior of bacteria and thus become ineffective and unable to play their role, resulting in antibiotic resistance. Here in the HKE9 chromosome, OmpK37, not OmpK35 and OmpK36, was retrieved. The genes and mutations are shown in **Tables 1** and **3**.

**Table 3:** The susceptibility and resistance gene and mutation in HvKP/HmKP.

Resistance mechanism	Protein homolog model	Protein variant model	Drug class
Antibiotic efflux	CrcB, baeR		Aminoglycoside antibiotic
	LptD		Carbapenem; peptide antibiotic; aminocoumarin antibiotic; rifamycin antibiotic
	e*mrR*, *rsmA*, *oqxA*, *adeF*, *marA*		Fluoroquinolone antibiotic
	*Klebsiella pneumoniae* KpnF, KpnE, KpnH, KpnG, H-NS, CRP		Macrolide antibiotic; aminoglycoside antibiotic; cephalosporin; tetracycline antibiotic; peptide antibiotic; rifamycin antibiotic
	*msbA*		Nitroimidazole antibiotic
Antibiotic inactivation	SHV-1		Carbapenem; cephalosporin; penam
	CTX-M-15, *Escherichia coli ampH* beta-lactamase		Cephalosporin
	FosA6		Fosfomycin
Antibiotic target alteration	*Haemophilus influenzae* PBP3 conferring resistance to beta-lactam antibiotics	*H. influenzae* PBP3 conferring resistance to beta-lactam antibiotics D350N, S357N	Cephalosporin; cephamycin; penam
		*E. coli* EF-Tu mutants conferring resistance to Pulvomycin (R234F)	Elfamycin antibiotic
		*Salmonella enterica gyrA* conferring resistance to fluoroquinolones (S83F)	Fluoroquinolone antibiotic
		*E. coli* UhpT with mutation conferring resistance to fosfomycin (E350Q)	Fosfomycin
	*eptB*, *ArnT*		Peptide antibiotic
rreduced permeability to antibiotic	*K. pneumoniae* OmpK37, OmpK37		Monobactam; carbapenem; cephalosporin; cephamycin; penam; penem

### 3.4 Bacterial siderophore production and the related genes

HKE9 could produce siderophores and generate an orange autocrine loop on CAS agar. The relative amount of siderophores was 7.90% ± 0.05% in HKE9 and 1.5% ± 0.04% in *K. pneumoniae* ATCC 700603. The bacterial siderophore genes in the genome are listed in **Table 4**. Enterobactin genes and yersiniabactin genes are in the chromosome. The aerobactin genes are in the circular plasmid. The salmochelin gene *iroE* is in the chromosome, while *iroN*, *iroB*, *iroC*, and *iroD* are in the circular plasmid.

**Table 4:** Comparative pathogenomics of four representative *K. pneumoniae* str strains with HKE9.

Virulence factors	Related genes	Strain 1084	Strain HS11286	Strain MGH 78578	Strain NTUH-K2044		Strain HKE9	
		Chromosome NC_018522 (5,386,705 bp)	Chromosome NC_016845 (5,333,942 bp)	Chromosome NC_009648 (5,315,120 bp)	Chromosome NC_012731 (5,248,520 bp)	pK2044 NC_006625 (224,152 bp)	Chromosome (5,320,954 bp)	Plasmid (161,792 bp)
Adherence								
Type I fimbriae	*fimB*	+	+	+	+	−	+	−
	*fimE*	+	+	+	+	−	+	−
	*fimA*	+	+	+	+	−	+	−
	*fimI*	+	+	+	+	−	+	−
	*fimC*	+	+	+	+	−	+	−
	*fimD*	+	+	+	+	−	+	−
	*fimF*	+	+	+	+	−	+	−
	*fimG*	+	+	+	+	−	+	−
	*fimH*	+	+	+	+	−	+	−
	*fimK*	+	+	+	+	−	+	−
Effector delivery system								
T6SS	*vipA/tssB*	+	+	+	+	−	+	−
	*vipB/tssC*	+	+	+	+	−	+	−
	*vase/tssK*	+	+	+	+	−	+	−
	*dotU/tssL*	+	+	+	+	−	+	−
	*ompA*	+	+	+	+	−	+	−
	*hcp/tssD*	+	+	+	+	−	+	−
	*clpV/tssH*	+	+	+	+	−	+	−
	*vgrG/tssI*	+	+	+	+	−	+	−
	*tli1*	−	+	−	−	−	−	−
	*tle1*	−	+	−	−	−	+	−
	*icmF/tssM*	+	+	+	+	−	+	−
	*impA/tssA*	+	+	+	+	−	+	−
	*tssF*	+	+	+	+	−	+	−
	*tssG*	+	+	−	+	−	+	−
	*sciN/tssJ*	+	+	−	+	−	+	−
T6SS-II	*impF*	−	−	+	−	−	−	−
	*sciN*	−	−	−	−	−	−	−
	*impH*	−	−	+	−	−	−	−
	*vasa/impG*	−	−	+	−	−	−	−
	*icmF*	−	−	+	−	−	−	−
	*vgrG*	−	−	+	−	−	−	−
	*ompA*	−	−	+	−	−	−	−
	*dotU*	−	−	−	−	−	−	−
	*impJ*	−	−	+	−	−	−	−
	*clpV*	+	+	+	+	−	−	−
T6SS-III	*impJ*	+	+	+	+	−	−	−
	*dotU*	+	+	−	+	−	+	−
	*ompA*	+	+	+	+	−	+	−
	*vgrG*	+	+	+	+	−	+	−
	*lysM*	−	−	−	−	−	−	−
	*icmF*	+	+	+	+	−	+	−
	*impG*	+	+	+	+	−	−	−
	*impH*	+	+	+	+	−	−	−
	*sciN*	+	+	+	+	−	+	−
	*impF*	+	+	−	+	−	−	−
	*impA*	+	+	+	+	−	−	−
Exotoxin								
Colibactin	*clbA*	+	−	−	−	−	−	−
	*clbB*	+	−	−	−	−	+	−
	*clbC*	+	−	−	−	−	−	−
	*clbD*	+	−	−	−	−	+	−
	*clbE*	+	−	−	−	−	−	−
	*clbF*	+	−	−	−	−	+	−
	*clbG*	+	−	−	−	−	+	−
	*clbH*	+	−	−	−	−	−	−
	*clbI*	+	−	−	−	−	+	−
	*clbJ*	+	−	−	−	−	−	−
	*clbK*	+	−	−	−	−	−	−
	*clbL*	+	−	−	−	−	+	−
	*clbM*	+	−	−	−	−	+	−
	*clbN*	+	−	−	−	−	−	−
	*clbO*	+	−	−	−	−	−	−
	*clbP*	+	−	−	−	−	+	−
	*clbQ*	+	−	−	−	−	−	−
	*clbS*	+	−	−	−	−	−	−
Immune modulation								
Capsule	–	+	+	+	+	−	+	−
LPS	–	+	+	+	+	−	+	−
Biofilm								
Type 3 fimbriae	*mrkH*	+	+	−	+	−	+	−
	*mrkI*	+	+	+	+	−	+	−
	*mrkJ*	+	+	+	+	−	+	−
	*mrkF*	+	+	+	+	−	+	−
	*mrkD*	+	+	+	+	−	+	−
	*mrkC*	+	+	+	+	−	+	−
	*mrkB*	+	+	+	+	−	+	−
	*mrkA*	+	+	+	+	−	+	−
Nutritional/Metabolic factor								
Aerobactin	*iucA*	−	−	−	−	+	−	+
	*iucB*	−	−	−	−	+	−	+
	*iucC*	−	−	−	−	+	−	+
	*iucD*	−	−	−	−	+	−	+
	*iutA*	+	+	+	+	+	+	+
Allantoin utilization	*allS*	+	−	−	+	−	+	−
	*allA*	+	−	−	+	−	+	−
	*allR*	+	−	−	+	−	+	−
	*allB*	+	−	−	+	−	+	−
	*allC*	+	−	−	+	−	+	−
	*allD*	+	−	−	+	−	+	−
Ent	*entA*	+	+	+	+	−	+	−
	*entB*	+	+	+	+	−	+	−
	*entE*	+	+	+	+	−	+	−
	*entC*	+	+	+	+	−	+	−
	*fepB*	+	+	+	+	−	+	−
	*entS*	+	+	+	+	−	−	−
	*fepD*	+	+	+	+	−	+	−
	*fepG*	+	+	+	+	−	+	−
	*fepC*	+	+	+	+	−	+	−
	*entF*	+	+	+	+	−	+	−
	*fes*	+	+	+	+	−	+	−
	*fepA*	+	+	+	+	−	+	−
	*entD*	+	+	+	+	−	+	−
Sal	*iroE*	+	+	+	+	−	+	−
	*iroN*	+	+	+	+	+	+	+
	*iroB*	+	−	−	+	+	−	+
	*iroC*	−	−	−	+	+	−	+
	*iroD*	+	−	−	+	+	−	+
Ybt	*ybtS*	+	+	−	+	−	+	−
	*ybtX*	+	+	−	+	−	+	−
	*ybtQ*	+	+	−	+	−	+	−
	*ybtP*	+	+	−	+	−	+	−
	*ybtA*	+	+	−	+	−	+	−
	*irp2*	+	+	−	+	−	+	−
	*irp1*	+	+	−	+	−	+	−
	*ybtU*	+	+	−	+	−	+	−
	*ybtT*	+	+	−	+	−	+	−
	*ybtE*	+	+	−	+	−	+	−
	*fyuA*	+	+	−	+	−	+	−
Antimicrobial activity/competitive advantage								
AcrAB	*acrA*	+	+	+	+	−	+	−
	*acrB*	+	+	+	+	−	+	−
Regulation								
RcsAB	*rcsA*	+	+	+	+	−	+	−
	*rcsB*	+	+	+	+	−	+	−
RmpA	*rmpA*	−	−	−	+	+	−	−
	*rmpA2*	−	−	−	−	−	−	−

### 3.5 Virulence factors and the related genes

The pathogenomics of HKE9 and four other *K. pneumoniae* strains was compared, and results are listed in **Table 4** and **Table S3**. All strains had virulence factors including adherence (type 1 fimbriae), effector delivery system (T6SS, T6SS II, and T6SS III), immune modulation (capsule and LPS), biofilm (type 3 fimbriae), nutritional/metabolic factor (aerobactin, allantoin utilization, ent, and sal), and antimicrobial activity/competitive advantage (AcrAB).

For the effector delivery system, 13 core genes in T6SS were retrieved in strains 1084, HS11286, NTUH-K2044, and HKE9. Strain HS11286 has both *tli1* and *tle1*, which were type VI lipase immunity and type VI lipase effector, respectively. HKE9 has only *tle1*. For T6SS II, only strain MGH78578 harbors the clusters. For T6SS III, all five strains have partial core genes.

For exotoxin, only strains 1084 and HKE9 have colibactin coding genes in the chromosome. Most of the aerobactin coding genes including *iucA*, *iucB*, *iucC*, and *iucD* were retrieved in plasmids of strains NTUH-K2044 and HKE9. *iutA* was detected widely in all strains. The genes related to allantoin utilization were retrieved in the chromosome of strains 1084, NTUH-K2044, and HKE9. *Ybt* was retrieved in strains 1084, HS11286, and HKE9. Except for strain NTUH-K2044, there was no *rmpA* either in the chromosome or plasmid of HKE9 and other strains.

### 3.6 Genomic evolutionary analysis of *rpoS*

Through phylogenetic tree construction, it was found that HKE9 did not belong to the same group as the reported strains of drug resistance, high toxicity, high viscosity, and other characteristics (**Figure 2D**). However, it is similar to *K. pneumoniae* Kp52.145.

### 3.7 Mechanism exploration of global regulatory factor *rpoS* in HKE9

#### 3.7.1 Effect of *rpoS* on hemolysis activity, mucoviscosity, and antibacterial susceptibility

HKE9, HKE9-M-*rpoS*, and HKE9-C-M-*rpoS* showed no hemolytic activity (**Figures 1C–E**), and the string tests were all positive. The semi-quantitative viscosity of HKE9-M-*rpoS* was higher than that of HKE9 and HKE9-C-M-*rpoS* (0.070 *vs.* 0.089, *p* = 0.05; 0.089 *vs.* 0.066, *p* = 0.027) (**Figure 1B**). The minimum inhibitory concentration (MIC) values showed no statistical difference between HKE9 and HKE9-M-*rpoS* (**Table 2**).

#### 3.7.2 Effect of *rpoS* on the virulence to *G. mellonella* larvae

**Figure 3:**
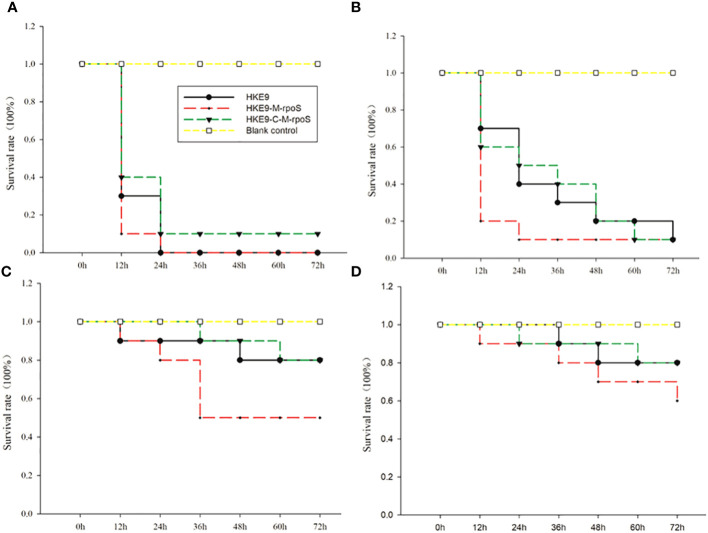
Survival curves of *Galleria mellonella* larvae were recorded for 72 h after being infected with 10 μL of HKE9, HKE9-M-*rpoS*, and HKE9-C-M-*rpoS* at concentrations of 1 × 10^6^ CFU/mL **(A)**, 1 × 10^5^ CFU/mL **(B)**, 1 × 10^4^ CFU/mL, **(C)** and 1 × 10^3^ CFU/mL **(D)**, and 10 μL of LB medium was used as negative control.

It was shown from the survival rate of *G. mellonella* larvae infected with 1 × 10^3^ CFU/mL, 1 × 10^4^ CFU/mL, 1 × 10^5^ CFU/mL, and 1 × 10^6^ CFU/mL of HKE9, HKE9-M-*rpoS*, and HKE9-C-M-*rpoS* that *rpoS* had a negative effect on the virulence of *K. pneumoniae*. For the larvae infected with 1 × 10^3^ CFU/mL, 1 × 10^4^ CFU/mL, 1 × 10^5^ CFU/mL, and 1 × 10^6^ CFU/mL of HKE9-M-*rpoS*, their survival rates were all lower than those of HKE9 and HKE9-C-M-*rpoS* (**Figure 3**), which indicated the higher toxicity in HKE9 without *rpoS*. At 12 h after infection, the IC_50_ values of HKE9, HKE9-M-*rpoS*, and HKE9-C-M-*rpoS* were 5.6 × 10^5^ CFU/mL, 3.8 × 10^4^ CFU/mL, and 5.4 × 10^5^ CFU/mL, respectively.

#### 3.7.3 Effect of *rpoS* on the tolerance of environmental stress

**Figure 4:**
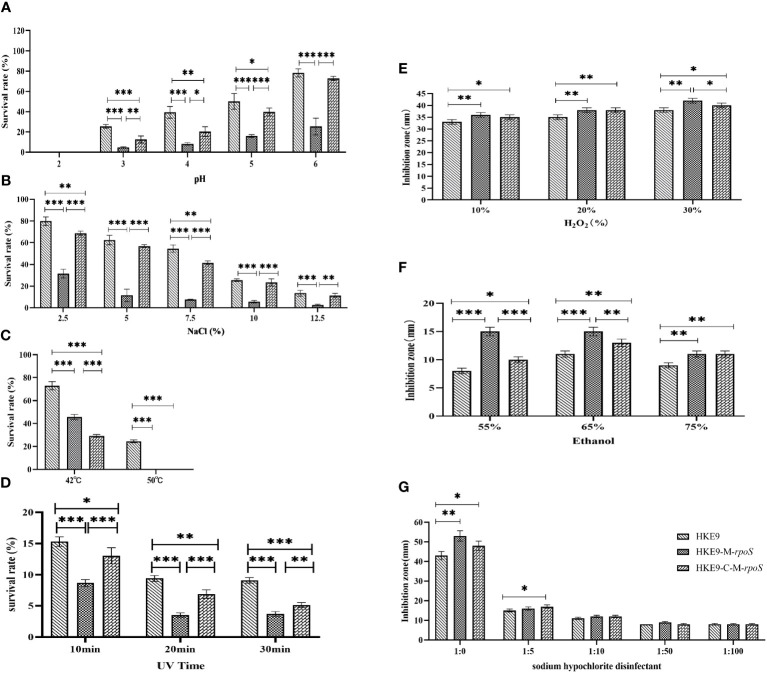
Tolerance of HKE9, HKE9-M-*rpoS*, and HKE9-C-M-*rpoS* to different environmental stress and disinfectants. **(A)** pH. **(B)** NaCl. **(C)** Temperature. **(D)** UV. **(E)** H_2_O_2_. **(F)** Alcohol. **(G)** Sodium hypochlorite disinfectant. *, P<0.05. **, P<0.01. ***, P<0.001.

For the tolerance to acidic pH, HKE9, HKE9-M-*rpoS*, and HKE9-C-M-*rpoS* could not survive at pH = 2. At pH values of 3, 4, 5, and 6, HKE9-M-*rpoS* had a significantly lower tolerance than HKE9 (4.79% *vs.* 24.45%, *p<* 0.001; 8.18% *vs.* 39.33%, *p<* 0.001; 15.90% *vs.* 50.10%, *p<* 0.001; 25.37% *vs.* 78.25%, *p<* 0.001); the tolerance of HKE9-C-M-*rpoS* to the acidic environment was significantly improved as compared with HKE9-M-*rpoS* (12.66% *vs.* 4.79%, *p<* 0.001; 20.40% *vs.* 8.18%, *p =* 0.014; 39.85% *vs.* 15.90%, *p =* 0.001; 72.73% *vs.* 25.37%, *p<* 0.001) (**Figure 4A**).

For hypertonic reaction, knockout *rpoS* significantly reduced the growth of HKE9 in 2.5%, 5.0%, 7.5%, 10%, and 12.5% NaCl (31.50% *vs.* 79.80%, *p<* 0.001; 11.44% *vs.* 62.40%, *p<* 0.001; 7.60% *vs.* 54.49%, *p<* 0.001; 5.65% *vs.* 25.49%, *p<* 0.001; 2.61% *vs.* 13.41%, *p =* 0.001). HKE9-C-M-*rpoS* had significantly increased tolerance to high osmotic pressure as compared with HKE9-M-*rpoS* (68.54% *vs.* 31.50%, *p<* 0.001; 51.81% *vs.* 11.44%, *p<* 0.001; 41.38% *vs.* 7.60%, *p<* 0.001; 23.38% *vs.* 5.65%, *p<* 0.001; 11.33% *vs.* 2.61%, *p* = 0.002) (**Figure 4B**).

After 1-h treatment at 42°C and 52°C, the survival rate of HKE9-M-*rpoS* was significantly lower than that of HKE9 (45.7% *vs.* 72.9%, *p<* 0.001; 0% *vs.* 24.5%, *p<* 0.001). HKE9-M-*rpoS* and HKE9-C-M-*rpoS* could not survive at 52°C (**Figure 4C**).

When exposed to ultraviolet at 10 min, 20 min, and 30 min, the survival rate of HKE9-M-*rpoS* was significantly lower than that of HKE9 (8.66% *vs.* 15.30%, *p<* 0.001; 3.50% *vs.* 9.40%, *p<* 0.001; 3.70% *vs.* 9.10%, *p<* 0.001). HKE9-C-M-*rpoS* had significantly increased tolerance UV than HKE9-M-*rpoS* (13.00% *vs.* 8.66%, *p* = 0.001; 6.88% *vs.* 3.50%, *p<* 0.001; 5.12% *vs.* 3.70%, *p* = 0.005) (**Figure 4D**). The results showed that *rpoS* impaired the ability of HKE9 in response to various environmental stresses.

#### 3.7.4 Effect of *rpoS* on the tolerance of disinfectant stress

After being treated with 10%, 20%, and 30% H_2_O_2_, the inhibition zone of HKE9-M-*rpoS* was significantly larger than that of HKE9 (*p* = 0.01, *p* = 0.01, and *p* = 0.003), and the increased susceptibility partially recovered in HKE9-C-M-*rpoS* without a statistical difference (*p* > 0.05) (**Figure 4E**). When treated with 55%, 65%, and 75% ethanol, it was found that compared with HKE9, HKE9-M-rpoS showed significantly higher sensitivity to ethanol (*p<* 0.001, *p<* 0.001, and *p<* 0.001), and the increased susceptibility was significantly recovered in HKE9-C-M-*rpoS* (*p* = 0.001, *p* = 0.01, and *p* = 0.01) (**Figure 4F**). In the sodium hypochlorite disinfectant-treated *K. pneumoniae*, the inhibition zone of HKE9-M-*rpoS* significantly increased by 10 mm when compared with HKE9 (*p* = 0.002), while the inhibition zone of HKE9-C-M-*rpoS* was significantly smaller than that of HKE9-M-*rpoS* (*p* = 0.044). However, there was no statistical significance in treatments with low concentrations of sodium hypochlorite disinfectant dilution solution (1:5, 1:10, 1:50, and 1:100) (*p* > 0.05) (**Figure 4G**). The results showed that knockout *rpoS* can increase the susceptibility of HKE9 to disinfectants.

#### 3.7.5 Effect of *rpoS* on bacterial competition

**Figure 5:**
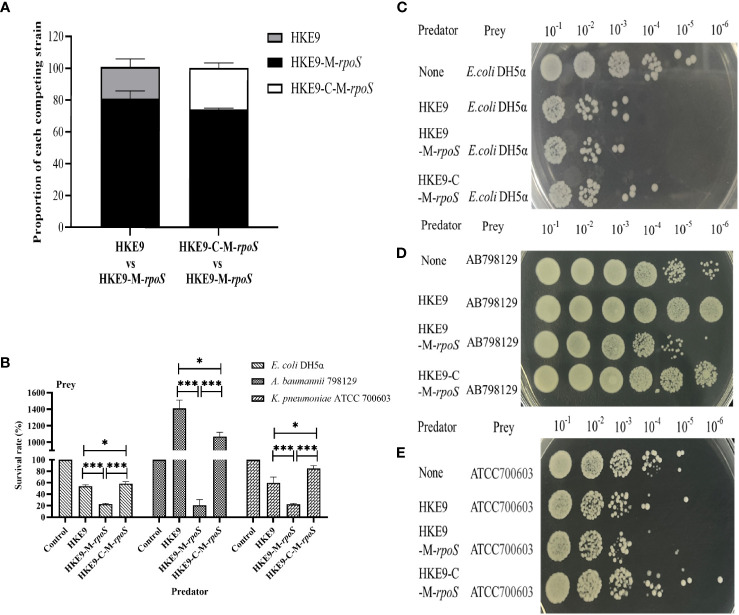
The regulation effect of *RpoS* in bacterial intraspecies and interspecies competitive growth. HKE9 and HKE9-C-M-*rpoS* were mixed with HKE9-M-*rpoS* in the ratio of 1:1 after 5 h of coincubation for self-competition **(A)**. Survival rates **(B)** and growth of the prey strains *Escherichia coli* DH5α **(C)**, *Acinetobacter baumannii* 798129 **(D)**, and *Klebsiella pneumoniae* ATCC 700603 **(E)** after 5 h of coincubation with the predator strains of HKE9, HKE9-M-*rpoS*, and HKE9-C-M-*rpoS*. *, P<0.05. ***, P<0.001.

In self-competition, HKE9-M-*rpoS* was more competitive than HKE9 and HKE9-C-M-*rpoS* (80.86% *vs.* 20.00%, *p<* 0.001; 26.2% *vs.* 80.86%, *p<* 0.001) (**Figure 5A**). When competing with *E. coli* DH5α, *A. baumannii* 798129, and *K. pneumoniae* ATCC 700603, HKE9-M-*rpoS* showed higher interspecies competitiveness and stronger bactericidal ability than HKE9, and the survival of preys was all significantly reduced (23.00% *vs.* 53.79%, *p<* 0.001; 20.00% *vs.* 1,400.00%, *p<* 0.001; 22.66% *vs.* 60.00%, *p<* 0.001) (**Figures 5B–E**). The results of the interspecies or intraspecies competition showed that the bacterial competition ability of HKE9 was negatively regulated by *rpoS*.

#### 3.7.6 Effect of *rpoS* on cell adhesion ability

**Figure 6:**
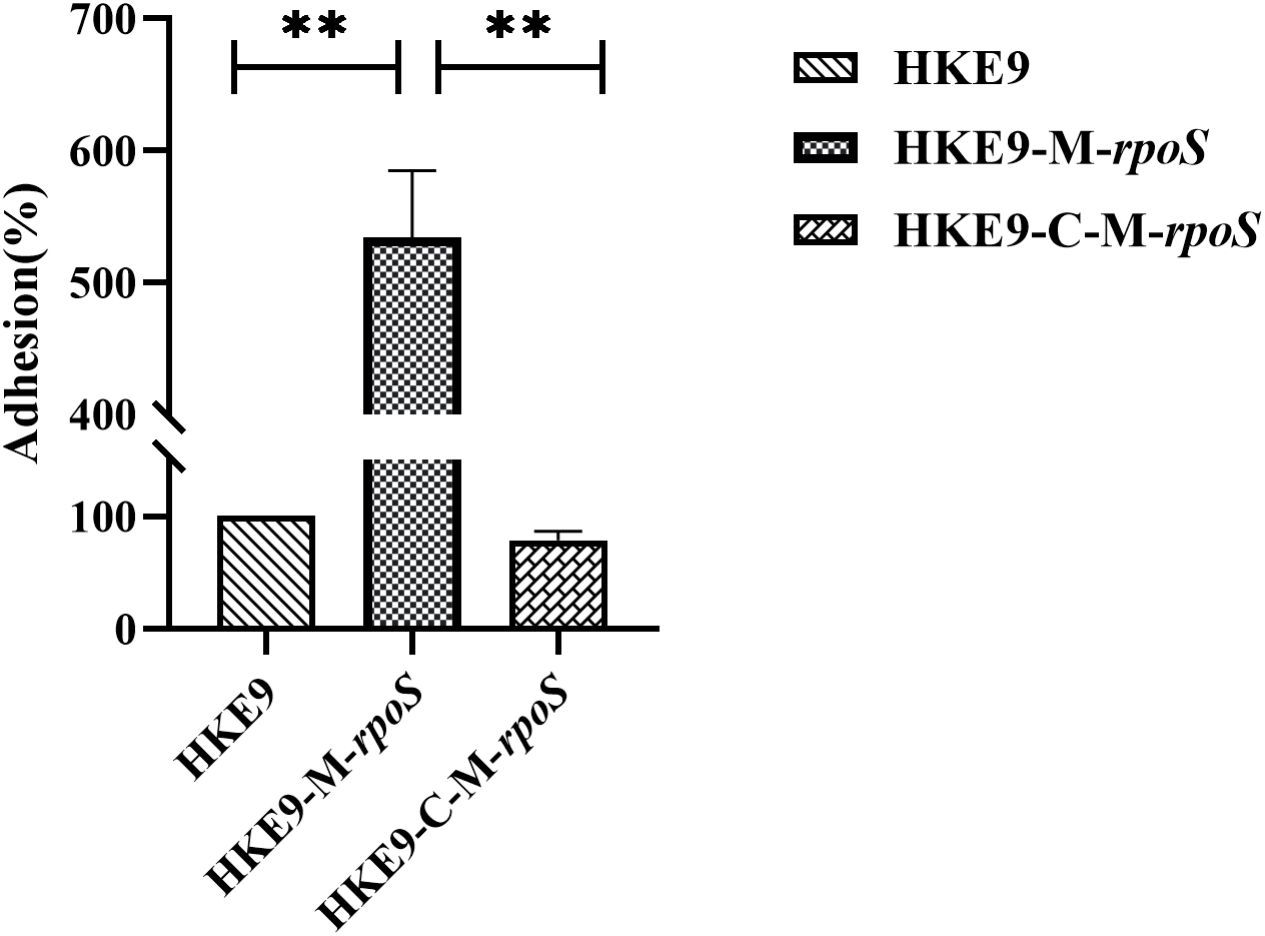
The cell adhesion assay. Human lung adenocarcinoma A549 cells in a 24-well plate (2.5 × 10^5^ cells per well) viability with incubation of *Klebsiella pneumoniae* HKE9, HKE9-M-*rpoS*, and HKE9-C-M-*rpoS* (OD_600_, 0.4–0.6) for 30 min, the amount of adhered *K. pneumoniae* cells was counted, and the relative adhesion rate of *K. pneumoniae* cells was calculated. **, P<0.01.

The relative adhesion of HKE9-M-*rpoS* to A549 cells was significantly increased by 5.2-fold (524.33% *vs.* 100%, *p<* 0.001). In contrast, the adhesion of HKE9-C-M-*rpoS* to A549 cells was not different from that of HKE9 (78.33% *vs.* 100%, *p* = 0.403) and significantly lower than that of HKE9-M-*rpoS* (*p<* 0.001) (**Figure 6**). The result indicated that knockout *rpoS* can increase the adhesion ability of HKE9 to A549 cells.

### 3.8 RNA-Seq analysis of HKE9, HKE9-M-*rpoS*, and HKE9-C-M-*rpoS*

It can be seen that there are significant differences in gene expression among the three groups of samples (**Figure S1A**). Compared with HKE9, HKE9-M-*rpoS* had 255 genes expressed significantly differently (FDR< 0.05 and |log2 FC| > 2), among which 21 genes were upregulated and 234 genes were downregulated (**Figure S1B**, **Table S4**); HKE9-C-M-*rpoS* had 289 genes expressed significantly differently, among which 72 genes were upregulated and 217 genes were downregulated (**Figure S1C**, **Table S5**). Compared with HKE9-M-*rpoS*, HKE9-C-M-*rpoS* had 124 genes expressed significantly differently, among which 84 genes were upregulated and 40 genes were downregulated (**Figure S1D**, **Table S6**).

#### 3.8.1 Gene expression changes associated with environmental stress

**Figure 7:**
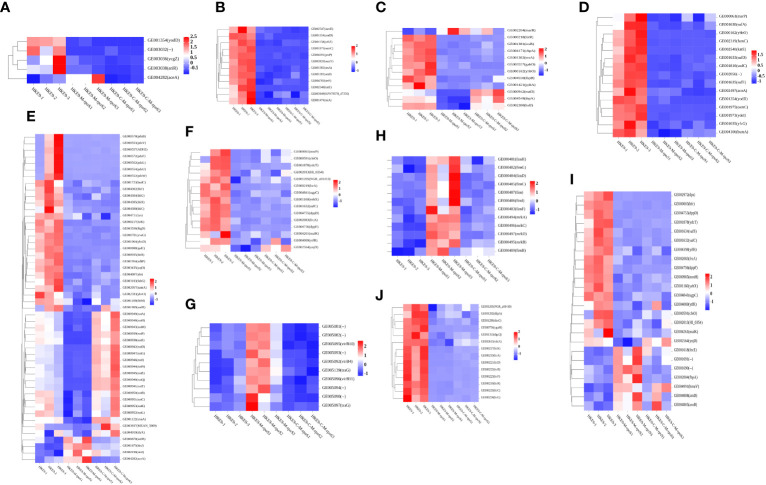
Transcriptomic analysis heat map of HKE9, HKE9-M-*rpoS*, and HKE9-C-M-*rpoS* gene expression. Environmental stress-related genes of acidic pH **(A)**, hyperosmotic pressure **(B)**, and heat shock **(C)**. Disinfectant-related genes of H_2_O_2_ **(D)**, alcohol **(E)**, and sodium hypochlorite disinfectant **(F)**. Gene expression in T4SS **(G)**. Gene expression in type 1 fimbriae and type 3 fimbriae **(H)**. Gene expression in transport of iron in cells **(I)**. Gene expression in quorum sensing **(J)**.

Knockout *rpoS* impaired the ability of HKE9 to cope with environmental stress, and transcriptome results confirmed it. The expressions of genes *yodD*, *GE00303*, *ycgZ*, and *ariR*, which are related to response to an acidic environment, decreased and *aceA* increased in HKE9-M-*rpoS* (**Figure 7A**). The expressions of *yodD*, *YcgZ*, and *ariR* were decreased by 20.27-fold, 5.16-fold, and 4.4-fold, respectively. *GE00303* expression was decreased from 15.2 to 0. The expression of *aceA* increased by 2.2 times.

The 12 genes’ expressions related to hyperosmotic pressure were downregulated. *osmC/E/Y* expressions in HKE9-M-*rpoS* were 31.68-fold, 4.69-fold, and 74-fold lower than those in HKE9 (**Figure 7B**). The 13 genes’ expressions related to heat shock in HKE9-M-*rpoS* were decreased (**Figure 7C**). The expressions of *cbpA*, *ibpA*, *ibpB*, *hsIJ*, *cspC*, and *YhbO* in HKE9-M-*rpoS* were decreased by 5-fold, 2.84-fold, 4.69-fold, 2.03-fold, 2-fold, and 49-fold, respectively.

#### 3.8.2 Gene expression associated with disinfectants

The 15 genes expressed in HKE9-M-*rpoS* related to oxidative stress decreased (**Figure 7D**). The expressions of *fumC*, *sodc*, *katE*, *osmC*, *yfcG*, and *AcnA* decreased by 22.19-fold, 11.76-fold, 35.01-fold, 31.68-fold, 9.14-fold, and 3.88-fold compared with HKE9, respectively.

The 50 genes related to alcohol metabolism and decomposition expressed differently (**Figure 7E**), among which 46 genes in HKE9-M-*rpoS* were downregulated and four genes were upregulated. The expression of *ADH2* decreased by 86.9-fold. *AdhP*, *name*, and *ahr* were reduced by 4.54-fold, 5.21-fold, and 2.08-fold, respectively.

The 15 genes in HKE9-M-*rpoS* were downregulated (**Figure 7F**). The expressions of *CbiO*, *ydcT*, *HI_0354*, *NGR_a01410*, *Ugpc*, *yehX*, *dppD*, *dppF*, *ytfR*, and *ynjD* were decreased by 3.83-fold, 8.34-fold, 2.45-fold, 2.44-fold, 3.21-fold, 9.09-fold, 7.32-fold, 5.53-fold, 2.26-fold, and 3.17-fold, respectively. The expression of *msrP* decreased by 2.73-fold.

#### 3.8.3 Gene expression of T4SS secretion system

In HKE9-M-*rpoS*, T6SS-related genes did not change differently, while all 10 genes involved in T4SS were upregulated (**Figure 7G**). *GE005081*, *GE005082*, *GE005090*, *GE005092* (*virB4*), *GE005093*, *GE005094*, *GE005095* (*virB10*), *GE005096* (*virB11*), *GE005097* (*traG*), and *GE005139* (*traG*) were 2.31, 2.42, 2.47, 2.36, 2.59, 2.54, 2.03, 2.25, 3.08, and 2.94 times higher than those in HKE9. All 10 genes are located in the linear plasmid. It was speculated that the unregulated T4SS gene might be related to the stronger competitive ability of HKE9-M-*rpoS*.

#### 3.8.4 Virulence-related gene expression

Type 1 and type 3 fimbriae of *K. pneumoniae* help bacterial cells adhere to the host. The adhesion ability of HKE9-M-*rpoS* to A549 cells was 5.2 times higher than that of HKE9. Eight genes’ expressions encoding type 1 fimbriae of HKE9-M-*rpoS* (*fim*, *fimB*, *fimC*, *fimD*, *fimF*, *fimG*, *fimH*, and *fimI*) were upregulated 4.97, 2.07, 3.48, 2.63, 2.50, 3.49, 2.77, and 4.22 times, respectively. Four genes’ expressions encoding type 3 fimbriae of HKE9-M-*rpoS* (*mrkA*, *mrkB*, *mrkC*, and *mrkD*) were upregulated 21, 3.60, 2.87, and 2.27 times, respectively (**Figure 7H**).

*RpoS* had an effect on the ferric transport and quorum sensing in *K. pneumoniae*. There were 24 genes’ expressions involved in iron transport, which changed in HKE9-M-*rpoS* (**Figure 7I**). Among them, there were seven genes upregulated and 17 genes downregulated. *afuA*, *afuB*, *fbpA*, *fecE* (Fe^3+^ dicitrate transport ATP-binding protein FecE), *hmuV* (a component of the ferric carrier transport system), a*rnB*, and *arnD* expressions of HKE9-M-*rpoS* were upregulated 2.8, 4.43, 2.6, 2.5, 2.2, 2.05, and 3.6 times, respectively. However, genes *dps*, *bfr*, *sufC/S*, *yifB*, *mntH*-related ferritin, and divalent metal cation transporter were decreased by 10.69, 9.31, 4.20, 4.39, 3.04, and 2.89 times, respectively.

In quorum sensing, 14 genes downregulated in HKE9-M-*rpoS* (**Figure 7J**). *lsrK*, *lsrR*, *lsrA*, *lsrC*, *lsrD*, *lsrB*, *lsrF*, and *lsrG* were downregulated 27.75, 17.86, 58.93, 51.62, 125.72, 40.98, 21.02, and 22.27 times, respectively. *DdpA* (d-dipeptide-binding periplasmic protein), *dosC* (diguanylate cyclase C), *dgcQ* (diguanylate cyclase Q), and *vdcA* (diguanylate cyclase) were decreased by 2.82, 4.80, 2.14, and 2.43 times, respectively.

HKE9-C-M-*rpoS* had nine genes encoding fimbriae and eight genes related to ferritin recovered to the expression of HKE9 (FDR > 0.05). However, the expression level of HKE9-C-M-*rpoS* genes related to quorum sensing did not recover to HKE9.

#### 3.8.5 Quantitative RT-PCR validation

The qRT-PCR results of 10 genes (*sufC*, *ariR*, *virB5*, *cbpA*, *virB10*, *mrkC*, *virB1*, *katE*, *osmY*, and *fimC*) of HKE9-M-*rpoS* and HKE9-C-M-*rpoS* (**Figures S2A**, **S2B**) showed the consistent expression with RNA-Seq. A correlation coefficient (R^2^) of 0.9040 between expressed data in RNA-Seq and qRT-PCR confirmed the reliability of RNA-Seq data (**Figure S2C**).

## 4 Discussion

Classic *K. pneumoniae* (CKP) and HvKP are two unrelated groups, but recent data showed that HvKP gradually gained resistance (Lee et al., 2017). The coherence of increasing multidrug resistance and inherited virulence of HvKP might result in a clinical crisis. ESBL-HvKP HKE9 isolated in this study was a novel ST3355. It was reported that CR-HvKP strains ST11 and ST23 harbored hypervirulence and MDR simultaneously (Gu et al., 2018). For ST3355 and ST23, three loci of *infB*, *mdh*, and *pgi* were the same. For ST3355 and ST11, only two loci of *mdh* and *pgi* were the same. HKE9 has a significant difference with ST23 and ST11 strains, and it was supposed that the ESBL-HvKP strain can be spread through various clonal types.

The complex resistance mechanisms in HvKP indicate that it can acquire resistance through several routes. With the advent of CTX-M-15 enzymes in the 2000, plasmids encoding ESBL were increasingly described in *K. pneumoniae*. HKE9 carried a plasmid containing CTX-M-15, which is the same as the reported isolation of HvKP-producing CTX-M-15 (Su et al., 2008). The reduction, deletion, or mutation of outer membrane proteins is also the main mechanism of resistance to antibiotics in *K. pneumoniae*. The deletion of OmpK35 and OmpK36 combined with the expression of ESBL can lead to resistance to carbapenem in *K. pneumoniae*. HKE9 lacks OmpK35 and OmpK36, except OmpK37, but expression of OmpK37 did not change after *rpoS* knockout, which may indicate that RpoS did not affect the antimicrobial resistance in HKE9.

In this study, we found a decreased tolerance of HKE9-M-*rpoS* to environmental stress and disinfectants. Transcriptome results also verified the functional changes. Osmoregulatory genes, heat shock protein genes, cold shock-like protein CspC, and acid tolerance genes were downregulated. *YhbO* in HKE9-M-*rpoS* was decreased, and HKE9-M-*rpoS* became more sensitive to oxidation, heat, UV, and pH, which was consistent with the research results of Abdallah (Abdallah et al., 2016). The genes related to oxidative stress, ethanol, and chlorine-containing disinfectants were downregulated. *fumC*, a regulator of superoxide reaction (soxRS), was activated by the SoxRS oxidative stress regulatory system, and expression of *fumC* increased under iron restriction (Himpsl et al., 2020). *kat*E has been shown to be a Catalase HPII regulated by *rpoS* (Robbe-Saule et al., 2001; Tondo et al., 2020). The expressions of *eut* family genes, aldol dehydrogenase ADH2, and alcohol dehydrogenase *adhP* genes in HKE9-M-*rpoS* were decreased. *Eu*t genes are involved in the metabolism and transformation of ethanolamine, ethanol, and acetaldehyde (Huseby and Roth, 2013). *MsrP* is a newly discovered methionine sulfoxide reductase system involved in the repair of periplasmic proteins oxidized by hypochlorite (Juillan-Binard et al., 2017). However, HKE9 is only sensitive to high concentrations of chlorine-containing disinfectants, which may be because HKE9 has chlorine-containing disinfectant resistance genes *MarA* and *MarR* (Collao et al., 2012).

Bacterial siderophore genes including enterobactin, yersiniabactin, aerobactin, and salmochelin were annotated in the HKE9 genome, which were not affected by knockout of *rpoS* in HKE9-M-*rpoS*. In contrast, *rpoS* caused changes in genes related to iron ion transport. Dps, a universally conserved prokaryotic ferritin, uses its ferritin and DNA binding functions to respond to various environmental stresses (Orban and Finkel, 2022). The *bfr* encodes bacterial ferritin and affects the activity of iron oxidase. The *sufC/S* operon may be involved in oxidative stress and iron restriction in the assembly of [Fe-S] clusters to promote bacterial pathogenicity (Nachin et al., 2003). In *yifB*, with iron chelatase activity, the pathophysiological characteristics of survival in host tissues are related to nutrient competitive adaptation (Parker et al., 2020).

HvKP has higher virulence than CKP. The virulence determinants of HvKP are poorly understood except that virulence gene *rmpA*/*A2* and pK2044-like virulence large plasmid carries a siderophore (*iroBCD*/*iucABCD*) (Pomakova et al., 2012). The *G. mellonella* infection model showed that *rpoS* negatively affects the virulence of HKE9. Together with the decreased resistance to the environmental stress and disinfectant and the increased bacterial competitiveness and cell adhesion ability in *rpoS* knockout HKE9, we guessed that the important regulation effect of *rpoS* is the enhanced tolerance and resistance to environmental stress and disinfectants, which may be at the cost of reducing virulence. When exploring the influence of *rpoS* on adhesion in cell infection, we found that the absence of *rpoS* could upregulate genes encoding type 1 and type 3 fimbriae. Type 1 fimbriae participated in the adhesion of PLA *K. pneumoniae* (Hsieh et al., 2016) and mediated adhesion of *K. pneumoniae* (Stahlhut et al., 2009). Type 3 fimbriae-dependent adhesion may be the first step in the colonization of *K. pneumoniae* (Schroll et al., 2010).

To clarify the virulence factors involved in pathogenicity, the pathogenomics of HKE9 was compared with that of four *K. pneumoniae* strains. Previous studies have found that T6SS effector protein is a newly discovered virulence factor in *K. pneumoniae* (Hsieh et al., 2019). Tle1 was found to be an effector protein transferred by T6SS, and the sub-inhibitory concentration of β-lactam antibiotics might be an impact factor for T6SS secretion and antibacterial activity (Liu et al., 2017). There exists a *tle1* in T6SS clusters of HKE9, which might have a similar effect via the Tle1 effector.

The *rpoS* positively regulates T6SS (Storey et al., 2020), and T6SS mutant pathogenic bacteria are less competitive than the wild type (Robbe-Saule et al., 2001). However, in this study, HKE9-M-*rpoS* was more competitive than HKE9, regardless of interspecies or intraspecies competition. The transcriptomic analysis showed that it was T4SS, not T6SS, gene expression that changed. There were 10 genes encoding T4SS upregulated. T4SS has two gene clusters in the HKE9 genome: one on the chromosome and the other on the linear plasmid. These upregulated T4SS genes are present on linear plasmids, and there was no change in T4SS gene expression on the chromosome. In ESKAPE bacteria, *bla*_CTX-M_ gene usually spreads rapidly through plasmids (D'Andrea et al., 2013). Compared with other bacterial secretion systems, T4SSs have the capacity to transport a variety of molecular substrates to target prokaryotic or eukaryotic cells. We speculated that the upregulated expression of T4SS gene on the mutant might be related to the competitive ability of HKE9.

## 5 Conclusions

Recent epidemiological data in clinical HvKP isolates from China were warning globally of high antimicrobial resistance, which will be an urgent priority to recognize and control these strains. In this study, an ST3355/K20/O2a ESBL-HvKP strain HKE9, isolated from the blood of an outpatient, may spread via various clonal types, where CTX-M-15, OmpK35(−), OmpK36(−), and Ompk37(+) were involved in the resistance mechanism, and Tle1 of T6SS, colibactin, and allantoin utilization had unique significance in pathogenicity. The most important regulation effect of global regulatory factor *rpoS* in HKE9 is the enhanced tolerance and resistance to environmental stress and disinfectants, which may be involved with T4SS, not T6SS. Thus, in pathogen infection control, the excessive and unnecessary use of polluted disposal, sterilization, and disinfection should be taken into consideration.

## Data availability statement

The datasets presented in this study can be found in online repositories. The names of the repository/repositories and accession number(s) can be found below: https://www.ncbi.nlm.nih.gov/genbank/, CP089768.

## Ethics statement

This study was approved by the ethics committee of the First Affiliated Hospital of Xi ’an Jiaotong University (No.XJTU1AF2019LSK-124) signed on November 26, 2019, and the need for informed consent was waived. Moreover, we confirm that all methods were performed in accordance with the relevant guidelines and regulations (Declaration of Helsinki).

## Author contributions

YZ: Writing – original draft, Formal Analysis, Methodology, Validation. YC: Investigation, Software, Writing – review & editing. TM: Investigation, Writing – review & editing, Data curation, Project administration. JW: Writing – review & editing, Resources, Validation. SL: Writing – review & editing, Data curation, Methodology, Visualization. JW: Data curation, Visualization, Writing – review & editing, Formal Analysis. LH: Writing – review & editing, Conceptualization, Software. XH: Writing – review & editing, Data curation, Validation, Visualization. XM: Data curation, Validation, Visualization, Writing – review & editing. SJ: Data curation, Validation, Writing – review & editing. PL: Validation, Writing – review & editing, Visualization. JL: Visualization, Writing – review & editing, Investigation. RD: Writing – review & editing, Conceptualization, Funding acquisition, Methodology, Resources, Supervision. BH: Conceptualization, Funding acquisition, Writing – review & editing, Supervision, Writing – original draft.

## References

[B1] Abdallah J. Mihoub M. Gautier V. Richarme G. (2016). The DJ-1 superfamily members YhbO and YajL from *Escherichia coli* repair proteins from glycation by methylglyoxal and glyoxal. Biochem. Biophys. Res. Commun. 470, 282–286. doi: 10.1016/j.bbrc.2016.01.068 26774339

[B2] Anzai E. K. de Souza J. C. Peruchi A. R. Fonseca J. M. Gumpl E. K. Pignatari A. C. C.. (2017). First case report of non-human primates (*Alouatta clamitans*) with the hypervirulent *Klebsiella pneumoniae* serotype K1 strain ST 23: A possible emerging wildlife pathogen. J. Med. Primatol. 46, 337–342. doi: 10.1111/jmp.12296 28809435

[B3] Chiang M. K. Lu M. C. Liu L. C. Lin C. T. Lai Y. C. (2011). Impact of Hfq on global gene expression and virulence in *Klebsiella pneumoniae* . PloS One 6, e22248. doi: 10.1371/journal.pone.0022248 21779404 PMC3136514

[B4] Collao B. Morales E. H. Gil F. Polanco R. Calderón I. L. Saavedra C. P. (2012). Differential expression of the transcription factors *MarA, Rob*, and *SoxS* of *Salmonella typhimurium* in response to sodium hypochlorite: down-regulation of rob by MarA and SoxS. Arch. Microbiol. 194, 933–942. doi: 10.1007/s00203-012-0828-8 22752112

[B5] D'Andrea M. M. Arena F. Pallecchi L. Rossolini G. M. (2013). CTX-M-type β-lactamases: a successful story of antibiotic resistance. Int. J. Med. Microbiol. 303, 305–317. doi: 10.1016/j.ijmm.2013.02.008 23490927

[B6] Datsenko K. A. Wanner B. L. (2000). One-step inactivation of chromosomal genes in *Escherichia coli* K-12 using PCR products. Proc. Natl. Acad. Sci. 97, 6640–6645. doi: 10.1073/pnas.120163297 10829079 PMC18686

[B7] Flury B. B. Donà V. Buetti N. Furrer H. Endimiani A. (2017). First two cases of severe multifocal infections caused by *Klebsiella pneumoniae* in Switzerland: characterization of an atypical non-K1/K2-serotype strain causing liver abscess and endocarditis. J. Glob. Antimicrob. Resist. 10, 165–170. doi: 10.1016/j.jgar.2017.04.006 28729207

[B8] Gu D. Dong N. Zheng Z. W. Lin D. Huang M. Wang L. H. (2018). A fatal outbreak of ST11 carbapenem-resistant hypervirulent *Klebsiella pneumoniae* in a Chinese hospital: a molecular epidemiological study. Lancet Infect. Dis. 18, 37–46. doi: 10.1016/S1473-3099(17)30489-9 28864030

[B9] Guo C. Yang X. W. Wu Y. R. Wu Y. R. Han Y. P. Yang R. F.. (2015). MLST-based inference of genetic diversity and population structure of clinical *Klebsiella pneumoniae*, China. Sci. Rep. 5, 7612. doi: 10.1038/srep07612 25556771 PMC5154592

[B10] Guo Y. Wang S. S. Zhan L. L. Jin Y. Duan J. J. Hao Z. H.. (2017). Microbiological and clinical characteristics of hypermucoviscous *Klebsiella pneumoniae* isolates associated with invasive infections in China. Front. Cell. Infect. Microbiol. 7. doi: 10.3389/fcimb.2017.00024 PMC5286779 28203549

[B11] Himpsl S. D. Shea A. E. Zora J. Stocki J. A. Stocki J. A. Alteri C. J.. (2020). The oxidative fumarase FumC is a key contributor for E. coli fitness under iron-limitation and during UTI. PloS Pathog. 16, e1008382. doi: 10.1371/journal.ppat.1008382 32106241 PMC7064253

[B12] Hsieh P. F. Lin T. L. Lee C. Z. Tsai S. F. Wang J. T. (2008). Serum-induced iron-acquisition systems and TonB contribute to virulence in *Klebsiella pneumoniae* causing primary pyogenic liver abscess. J. Infect. Dis. 197, 1717–1727. doi: 10.1086/588383 18433330

[B13] Hsieh P. F. Hsu C. R. Chen C. T. Lin T. L. Wang J. T. (2016). The *Klebsiella pneumoniae YfgL (BamB)* lipoprotein contributes to outer membrane protein biogenesis, type-1 fimbriae expression, anti-phagocytosis, and in *vivo* virulence. Virulence 7, 587–601. doi: 10.1080/21505594.2016.1171435 27029012 PMC5038167

[B14] Hsieh P. F. Lu Y. R. Lin T. L. Lai L. Y. Wang J. T. (2019). *Klebsiella pneumoniae* type VI secretion system contributes to bacterial competition, cell Invasion, type-1 fimbriae expression, and in *vivo* colonization. J. Infect. Dis. 219, 637–647. doi: 10.1093/infdis/jiy534 30202982 PMC6350951

[B15] Huseby D. L. Roth J. R. (2013). Evidence that a metabolic microcompartment contains and recycles private cofactor pools. J. Bacteriol. 195, 2864–2879. doi: 10.1128/JB.02179-12 23585538 PMC3697265

[B16] Johansson M. H. K. Bortolaia V. Tansirichaiya S. Aarestrup F. M. Roberts A. P. Petersen T. N. (2021). Detection of mobile genetic elements associated with antibiotic resistance in *Salmonella* enterica using a newly developed web tool: MobileElementFinder. J. Antimicrob. Chemother. 76, 101–109. doi: 10.1093/jac/dkaa390 33009809 PMC7729385

[B17] Juillan-Binard C. Picciocchi A. Andrieu J. P. Dupuy J. Petit-Hartlein I. Caux-Thang C.. (2017). A two-component NADPH oxidase (NOX)-like system in bacteria is involved in the electron transfer chain to the methionine sulfoxide reductase MsrP. J. Biol. Chem. 292, 2485–2494. doi: 10.1074/jbc.M116.752014 28028176 PMC5313115

[B18] Krzywinski M.. (2009). Circos: an information aesthetic for comparative genomics. Genome Res. 19, 1639–1645. doi: 10.1101/gr.092759.109 19541911 PMC2752132

[B19] Lam M. M. C. Schein J. Birol I. Connors J. Gascoyne R. Horsman D.. (2018). Genetic diversity, mobilisation and spread of the yersiniabactin-encoding mobile element ICEKp in *Klebsiella pneumoniae* populations. Microb. Genom. 4, e000196. doi: 10.1099/mgen.0.000196 29985125 PMC6202445

[B20] Langmead B. Salzberg S. L. (2012). Fast gapped-read alignment with Bowtie 2. Nat. Methods 9, 357–359. doi: 10.1038/nmeth.1923 22388286 PMC3322381

[B21] Lee C. R. Lee J. H. Park K. S. Jeon J. H. Kim Y. B. Cha C. J.. (2017). Antimicrobial resistance of hypervirulent *Klebsiella pneumoniae*: epidemiology, hypervirulence-associated determinants, and resistance mechanisms. Front. Cell. Infect. Microbiol. 7, 483. doi: 10.3389/fcimb.2017.00483 29209595 PMC5702448

[B22] Li B. Dewey C. N. (2011). RSEM: accurate transcript quantification from RNA-Seq data with or without a reference genome. BMC Bioinf. 12, 323. doi: 10.1186/1471-2105-12-323 PMC3163565 21816040

[B23] Liu L. Ye M. P. Li X. B. Li J. Deng Z. X. Yao Y. F.. (2017). Identification and characterization of an antibacterial type VI secretion system in the carbapenem-resistant strain *Klebsiella pneumoniae* HS11286. Front. Cell. Infect. Microbiol. 7, 442. doi: 10.3389/fcimb.2017.00442 29085808 PMC5649205

[B24] Liu M. Li X. B. Xie Y. Z. Bi D. X. Sun J. Y. Li J.. (2019). ICEberg 2.0: an updated database of bacterial integrative and conjugative elements. Nucleic Acids Res. 47, D660–D665. doi: 10.1093/nar/gky1123 30407568 PMC6323972

[B25] Maturana P. Martinez M. Noguera M. E. Santos N. C. Disalva E. A. Semorile L.. (2017). Lipid selectivity in novel antimicrobial peptides: Implication on antimicrobial and hemolytic activity. Colloids. Surf. B. Biointerfaces. 153, 152–159. doi: 10.1016/j.colsurfb.2017.02.003 28236791

[B26] Munoz-Price L. S. Poirel L. Bonomo R. A. Schwaber M. J. Daikos G. L. Cormican M.. (2013). Clinical epidemiology of the global expansion of *Klebsiella pneumoniae* carbapenemases. Lancet Infect. Dis. 13, 785–796. doi: 10.1016/S1473-3099(13)70190-7 23969216 PMC4673667

[B27] Nachin L. Loiseau L. Expert D. Barras F.. (2003). SufC: an unorthodox cytoplasmic ABC/ATPase required for [Fe-S] biogenesis under oxidative stress. EMBO J. 22, 427–437. doi: 10.1093/emboj/cdg061 12554644 PMC140745

[B28] Orban K. Finkel S. E. (2022). *Dps* is a universally conserved dual-action DNA-binding and ferritin protein. J. Bacteriol. 204, e0003622. doi: 10.1128/jb.00036-22 35380871 PMC9112962

[B29] Pan Y. J. Lin T. L. Hsu C. R. Wang J. T. (2011). Use of a Dictyostelium model for isolation of genetic loci associated with phagocytosis and virulence in *Klebsiella pneumoniae* . Infect. Immun. 79, 997–1006. doi: 10.1128/IAI.00906-10 21173313 PMC3067516

[B30] Parker A. C. Bergonia H. A. Seals N. L. Baccanale C. L. Rocha E. R. (2020). The *uroS* and *yifB* genes conserved among tetrapyrrole synthesizing-deficient Bacteroidales are involved in *Bacteroides fragilis* heme assimilation and survival in experimental intra-abdominal infection and intestinal colonization. Infect. Immun. 88, e00103-20. doi: 10.1128/IAI.00103-20 32457103 PMC7375757

[B31] Pomakova D. K. Hsiao C. B. Beanan J. M. Olson R. MacDonald U. Keynan Y. (2012). Clinical and phenotypic differences between classic and hypervirulent *Klebsiella pneumonia*: an emerging and under-recognized pathogenic variant. Eur. J. Clin. Microbiol. Infect. Dis. 31, 981–989. doi: 10.1007/s10096-011-1396-6 21918907

[B32] Pu D. Zhao J. K. Lu B. H. Zhang Y. L. Wu Y. L. Li Z. Y.. (2023). Within-host resistance evolution of a fatal ST11 hypervirulent carbapenem-resistant *Klebsiella pneumoniae* . Int. J. Antimicrob. Agents. 61, 106747. doi: 10.1016/j.ijantimicag 36758779

[B33] Robbe-Saule V. Coynault C. Ibanez-Ruiz M. Hermant D. Norel F. (2001). Identification of a non-haem catalase in *Salmonella* and its regulation by RpoS (sigmaS). Mol. Microbiol. 39, 1533–1545. doi: 10.1046/j.1365-2958.2001.02340.x 11260470

[B34] Scapaticci M. Biscaro M. Burelli F. Cadamuro L. Biscaro R. Bartolini A. (2017). A case of invasive infection caused by a highly virulent strain of *Klebsiella pneumoniae* displaying hypermucoviscosity in a patient with hepatic involvement without liver abscess. Infez. Med. 25, 362–365. 29286017

[B35] Schroll C. Barken K. B. Krogfelt K. A. Struve C. (2010). Role of type 1 and type 3 fimbriae in *Klebsiella pneumoniae* biofilm formation. BMC Microbiol. 10, 179. doi: 10.1186/1471-2180-10-179 20573190 PMC2911432

[B36] Schwyn B. Neilands J. B. (1987). Universal chemical assay for the detection and determination of siderophores. Anal. Biochem. 160, 47–56. doi: 10.1016/0003-2697(87)90612-9 2952030

[B37] Shankar C. Veeraraghavan B. Nabarro L. E. B. Ravi R. Ragupathi N. K. D. Rupali P. (2018). Whole genome analysis of hypervirulent *Klebsiella pneumoniae* isolates from community and hospital acquired bloodstream infection. BMC Microbiol. 18, 6. doi: 10.1186/s12866-017-1148-6 29433440 PMC5809863

[B38] Shon A. S. Bajwa R. P. S. Russo T. A. (2013). Hypervirulent (hypermucoviscous) *Klebsiella pneumoniae*: a new and dangerous breed. Virulence 4, 107–118. doi: 10.4161/viru.22718 23302790 PMC3654609

[B39] Stahlhut S. G. Chattopadhyay S. Struve C. Weissman S. J. Aprikian P. Libby S. J.. (2009). Population variability of the FimH type 1 fimbrial adhesin in *Klebsiella pneumoniae* . J. Bacteriol. 191, 1941–1950. doi: 10.1128/JB.00601-08 19151141 PMC2648365

[B40] Storey D. McNally A. Åstrand M. Santos J. S. P. G. Rodriguez-Escudero I. Elmore B.. (2020). *Klebsiella pneumoniae* type VI secretion system-mediated microbial competition is PhoPQ controlled and reactive oxygen species dependent. PloS Pathog. 16, e1007969. doi: 10.1371/journal.ppat.1007969 32191774 PMC7108748

[B41] Su S. C. Siu L. K. Ma L. Yeh K. M. Fung C. P. Lin J. C.. (2008). Community-acquired liver abscess caused by serotype K1 *Klebsiella pneumoniae* with CTX-M-15-type extended-spectrum beta-lactamase. Antimicrob. Agents Chemother. 52, 804–805. doi: 10.1128/AAC.01269-07 18056273 PMC2224741

[B42] Tondo M. L. de Pedro-Jové R. Vandecaveye A. Piskulic L. Orellano E. G. Valls M. (2020). KatE from the bacterial plant pathogen *Ralstonia solanacearum* is a monofunctional catalase controlled by HrpG that plays a major role in bacterial survival to hydrogen peroxide. Front. Plant Sci. 11, 1156. doi: 10.3389/fpls.2020.01156 32849714 PMC7412880

[B43] Wick R. R. Heinz E. Holt K. E. Wyres K. L. (2018). Kaptive web: user-friendly capsule and lipopolysaccharide serotype prediction for *Klebsiella* genomes. J. Clin. Microbiol. 56, e00197-18. doi: 10.1128/JCM.00197-18 29618504 PMC5971559

[B44] Xie Y. Wei Y. Q. Shen Y. Li X. B. Zhou H. Tai C.. (2018). TADB 2.0: an updated database of bacterial type II toxin-antitoxin loci. Nucleic Acids Res. 46, D749–d753. doi: 10.1093/nar/gkx1033 29106666 PMC5753263

[B45] Zhang S. Mi P. Wang J. D. Li P. Luo K. Liu S. Y.. (2023). The optimized carbapenem inactivation method for objective and accurate detection of carbapenemase-producing *Acinetobacter baumannii* . Front. Microbiol. 14, 1185450. doi: 10.3389/fmicb.2023.1185450 37520356 PMC10372451

