# Supplementary material for: Transcriptomic and phenotype analysis revealed the role of rpoS in stress resistance and virulence of a novel ST3355 ESBL-producing hypervirulent Klebsiella pneumoniae isolate
Source: Front Cell Infect Microbiol. 2023 Oct 23;13:1259472. doi: 10.3389/fcimb.2023.1259472 (PMC10627032; doi:10.3389/fcimb.2023.1259472)
Supplement: Supplementary file 1 [file Table_1.docx]

Table S1. Bacterial strains, plasmids, and primers used in the present study.

Table S2 The toxin genes annotated with VFDB in two plasmids carried by HKE9.

Table S3 Comparative pathogenomics of *K. pneumoniae* strains*.*

Table S4 The DEGs of HKE9VSHKE9-M-rpoS with FDR < 0.05 and | log2FC | > 2.

Table S5 The DEGs of HKE9VSHKE9-C-M-*rpoS* with FDR < 0.05 and | log2FC | > 2.

Table S6 The DEGs of HKE9-M-*rpoS* VSHKE9-C-M-*rpoS* with FDR < 0.05 and | log2FC | > 1.

Figure S1 RNA-Seq features. PCA and Pearson correlation coefficient between HKE9, HKE9-M-*rpoS* and HKE9-C-M-*rpoS*(A); Volcanic maps between HKE9 and HKE9-M-*rpoS*(B); Volcanic maps between HKE9 and HKE9-C-M-*rpoS*(C); Volcanic maps between HKE9-M-*rpoS* and HKE9-C-M-*rpoS*(D).

Figure S2 Validation of transcriptome results and quantitative RT-PCR. HKE9 vs HKE9-M-*rpoS* RNA validation (A); HKE9 vs HKE9-C-M- *rpoS* RNA validation(B); quantitative RT-PCR validation (C).

Table S1. Bacterial strains, plasmids, and primers used in the present study.

| Strain, plasmid or primers | Genotype or comments | Source |
| --- | --- | --- |
| Strains | | |
| *Klebsiella pneumoniae* | | |
| *K. pneumoniae* ATCC 700603 | Kan^r^ | This study |
| HKE9 | Amp^r^, Clinical isolate, the parent strain for generation of isogenic mutant, the parent strain of mutant | This study |
| HKE9-M-*rpoS*  HKE9-C-M-*rpoS* | HKE9 isogenic mutant with a deletion of *rpoS*  HKE9-M-*rpoS* mutant carrying the pBAD33-*rpoS* plasmid | This study  This study |
| *Acinetobacter baumannii* | | |
| AB798129 | Kan^r^, Clinical isolate | This study |
| *Esherichia coli* | | |
| DH5ɑ | SupE44, DlacU169, *endA1*, *recA1*, *relA1* | This study |
| Plasmids | | |
| pK18mobsacB | Kan^r^, Integration vector | This study |
| pkd4 | Kan^r^, Amp^r^, *ori* R6Kgamma, rgnB | This study |
| pkd46-Tc | Tc^r^, repA101(ts), *ori*R101,*araBp-gam-bet-exo* | This study |
| pcp20 | Amp^r^, Cm^r^, ts-rep, [Ci857] (lambda)(ts), FLP | This study |
| pbad33 | Amp^r^, Cm^r^, Arabinose-inducible expression vector | This study |
| pbad33-*rpoS* | Cm^r^, pbad33 derivative containing the *rpoS* gene from HKE9 | This study |
| Primers (5’-3’) | | |
| Construction mutant in *K. pneumoniae* | | |
| *rpoS*-up-F | AAGGGGAAATCCGTCAACCC | This study |
| *rpoS*- up-R | TCCAGCCTACACAAT AAAGGTGGCTC | This study |
| *rpoS*-down- F | GGAGGATATTCATATGGACC TAAGACGTTTTC | This study |
| *rpoS*-down- R | GCCGCAGTGCTATATCGTGA | This study |
| Kan-FRT-F | GAGCCACCTTT ATTGTGTAGGCTGGAGCTGCTTC | This study |
| Kan-FRT-R | GTCTTA GGTCCATATGAATATCCTCCTTAGTTCC | This study |
| *rpoS*-Screen-F | GCTACCAAGATGTTGCCTAAT | This study |
| *rpoS*-Screen-R | GGTGAGAAGATGGACTACCCA | This study |
| pkd46-*bet*-F | ATGAGTACTGCACTCGCAACGCTGG | This study |
| pkd46-*bet*-R | TCATGCTGCCACCTTCTGCTCTGCG | This study |
| pcp20-F | GCCTACTAACGCTTGTCTTTG | This study |
| pcp20-R | TACCTTCAACCTCAAGCCAGA | This study |
| Construction complement in *K. pneumoniae* | | |
| *rpoS* – HB -F | TAGCGAATTCGAGCTGATCACGGGTAGGAGCCACCTTT | This study |
| *rpoS* – HB -R | CTGCAGGTCGACTCTAG CAGCAAGCGGAAGGGAAAAT | This study |
| pbad33-HB-F | TCCCTTCCGCTTGCTG CTAGAGTCGACCTGCAGGCA | This study |
| pbad33- HB -R | GCTCCTACCCGTGATCAGCTCGAATTCGCTAGCCCA | This study |
| KP -HB -Screen-F | CCATAAGATTAGCGGATCCTACCT | This study |
| KP-HB -Screen-R | CTTCTCTCATCCGCCAAAACAG | This study |
| RT-PCR |  | This study |
| KP-*16S* -RT-F | ATGACCAGCCACACTGGAAC | This study |
| KP-*16S* -RT-R | CTTCCTCCCCGCTGAAAGTG | This study |
| *rpoS*-RT-F | CGTCTATCTGCGTACCGC | This study |
| *rpoS* -RT-R | TTTCTGAATCGCCACCC | This study |
| *ariR-*16S -RT-F | CGCTCGCCATCAACA | This study |
| *ariR*-16S -RT-R | TAGCCAACGACAATCTCCA | This study |
| *katE-*16S -RT-F | GCTCAACGAACGCACCTC | This study |
| *katE*-16S -RT-R | ACCACCACCGCATCCAC | This study |
| *osmY-*16S -RT-F | CCAGCAATGCGGGAG | This study |
| *osmY*-16S -RT-R | GCTGAGGGTGACCACTTTATT | This study |
| *cbpA-*16S -RT-F | ATCAGCGTCGTCGTCAACA | This study |
| *cbpA*-16S -RT-R | TCGGCGTTTCGCTTTC | This study |
| *sufC-*16S -RT-F | TATCTTTATGGCCTTCCAGTAT | This study |
| *sufC*-16S -RT-R | TGCAGCAGCTTGATCTTTT | This study |
| *virB5-*16S -RT-F | AATGGGTTTAATGACGC | This study |
| *virB5*-16S -RT-R | TCGCCCTTTCCTGCA | This study |
| *virB10-*16S -RT-F | ATAGCCAACGGCACTCC | This study |
| *virB10*-16S -RT-R | TTCGCCTGGTTCCTGAT | This study |
| *virB1-*16S -RT-F | AAAGCAGGAGCGGCAGTC | This study |
| *virB1*-16S -RT-R | CGTTACCCGAATAATACAAAGA | This study |
| *fimC-*16S -RT-F | CGCAGGATCGGGAAAG | This study |
| *fimC*-16S -RT-R | CGCTACGGCTAAAGGTCA | This study |
| *mrkC-*16S -RT-F | AGGCGGGTCGGATAACA | This study |
| *mrkC*-16S -RT-R | GGGCGCTGGAGATAGGT | This study |

Table S2 The toxin genes annotated with VFDB in two plasmids carried by HKE9.

|  | gene_ID | VFDB gene | VFDB gene function | Virulence factor name | E-value | Identity% | Query-cover% | score |
| --- | --- | --- | --- | --- | --- | --- | --- | --- |
| plasmid1 | GE004935 | *trwK* | Trw type IV secretion sysmtem VirB4-like trwK protein | Trw type IV secretion system | 5.86E-07 | 22.32 | 36.18 | 50 |
|  | GE004962 | *cyaD* | cyclolysin secretion protein | Cya | 1.54E-19 | 24.76 | 92.6 | 87 |
|  | GE004963 | *hlyB* | hemolysin transport protein | Hemolysin | 1.38E-82 | 28.35 | 92.91 | 277 |
|  | GE004969 | *iroN* | salmochelin receptor IroN | Sal | 9.23E-07 | 21.25 | 95.42 | 48 |
|  | GE004974 | *bopD* | sugar-binding transcriptional regulator, LacI family | BopD | 1.76E-21 | 27.33 | 85.6 | 90 |
|  | GE004975 | *clbB* | colibactin hybrid non-ribosomal peptide synthetase/type I polyketide synthase ClbB | Colibactin | 5.26E-08 | 29.08 | 41.67 | 51 |
|  | GE004989 | *chuU* | heme permease protein ChuU | Chu | 8.18E-40 | 34.56 | 87.43 | 142 |
|  | GE004991 | *fepC* | ferrienterobactin ABC transporter ATPase | Enterobactin | 1.98E-38 | 34.6 | 83.64 | 135 |
|  | GE004996 | *entE* | 2,3-dihydroxybenzoate-AMP ligase component of enterobactin synthase multienzyme complex | Enterobactin | 7.35E-40 | 27.46 | 94.52 | 149 |
|  | GE005005 | *bfpH* | BfpH lipoprotein | BFP | 1.00E-17 | 37.19 | 70.99 | 73 |
|  | GE005016 | *iucA* | aerobactin Synthetase IucA | Aerobactin | 0 | 98.15 | 99.83 | 1202 |
|  | GE005017 | *iucB* | N-acetyltransferase IucB | Aerobactin | 0 | 99.05 | 99.68 | 642 |
|  | GE005018 | *iucC* | aerobactin siderophore biosynthesis protein IucC | Aerobactin | 0 | 99.31 | 99.83 | 1178 |
|  | GE005019 | *iucD* | lysine 6-monooxygenase IucD | Aerobactin | 0 | 98.35 | 95.5 | 877 |
|  | GE005020 | *iutA* | ferric aerobactin receptor IutA | Aerobactin | 0 | 99.73 | 99.86 | 1491 |
|  | GE005031 | *pvdS* | extracytoplasmic-function sigma-70 factor | Pyoverdine | 7.01E-12 | 31.54 | 72.83 | 57 |
|  | GE005033 | *bauA* | TonB-dependent siderophore receptor BauA | Acinetobactin | 1.67E-11 | 22.82 | 41.1 | 64 |
|  | GE005044 | *iroB* | glucosyltransferase IroB | Sal | 0 | 97.84 | 99.73 | 742 |
|  | GE005045 | *iroC* | ABC transporter | Sal | 0 | 98.93 | 99.92 | 2423 |
|  | GE005046 | *iroD* | siderophore esterase IroD | Sal | 0 | 99.26 | 99.51 | 823 |
|  | GE005047 | *iroN* | salmochelin receptor IroN | Sal | 0 | 93.23 | 99.86 | 1376 |
|  | GE005061 | *AHA_1389* | CobQ/CobB/MinD/ParA family protein | Polar flagella | 3.07E-12 | 31.48 | 40.72 | 63 |
|  | GE005070 | *afaG-VII* | AfaG-VII | Afimbrial adhesin, AFA-VII | 3.84E-08 | 31.4 | 17.17 | 49 |
|  | GE005076 | *etgA* | T3SS-associated peptidoglycan lytic enzyme | LEE encoded T3SS | 5.89E-36 | 44.36 | 81.37 | 122 |
| plasmid2 | GE005081 | *virB6-3* | type IV secretion system protein VirB6 family | Rvh T4SS | 7.31E-08 | 27.96 | 60.67 | 50 |
|  | GE005087 | *pilL* | type IV pilus biosynthesis protein PilL | Type IV pili | 8.77E-19 | 36.72 | 30.66 | 83 |
|  | GE005088 | *pilM* | type IV pilus inner membrane platform protein PilM | Type IV pili | 6.94E-16 | 28.95 | 75.86 | 68 |
|  | GE005090 | *virB1* | type IV secretion system attachment mediating protein VirB1 Homolog | VirB type IV secretion system | 7.86E-26 | 34.52 | 81.96 | 98 |
|  | GE005092 | *trwK* | Trw type IV secretion sysmtem VirB4-like trwK protein | Trw type IV secretion system | 1.02E-97 | 29.87 | 97.07 | 322 |
|  | GE005093 | *virB8* | type IV secretion system protein VirB8 | VirB type IV secretion system | 1.07E-33 | 32.2 | 95.08 | 121 |
|  | GE005094 | *ptlF* | Ptl Type IV secretion system outer membrane complex protein PtlF | Ptx | 7.21E-27 | 32.02 | 78.43 | 103 |
|  | GE005095 | *virB10* | type IV secretion system channel protein VirB10 | VirB type IV secretion system | 7.44E-49 | 37.79 | 63.06 | 166 |
|  | GE005096 | *trwD* | Trw type IV secretion sysmtem VirB11 homolog, trwD protein | Trw type IV secretion system | 4.82E-70 | 38.36 | 86.32 | 223 |
|  | GE005097 | *virD4* | type IV secretion system component VirD4 | Rvh T4SS | 1.36E-29 | 33.7 | 95.65 | 115 |
|  | GE005098 | *virD4* | type IV secretion system component VirD4 | Rvh T4SS | 2.64E-26 | 27.56 | 80.32 | 107 |
|  | GE005100 | *pilN* | type IV pilus inner membrane platform protein PilN | Type IV pili | 2.24E-108 | 38.28 | 97.26 | 336 |
|  | GE005101 | *pilO* | type IV pilus inner membrane platform protein PilO | Type IV pili | 1.22E-17 | 21.51 | 95.18 | 81 |
|  | GE005102 | *pilP* | type IV pilus biogenesis protein PilP | Type IV pili | 1.16E-09 | 29.27 | 71.35 | 51 |
|  | GE005103 | *pilQ* | type IV pilus biogenesis protein PilQ | Type IV pili | 3.17E-42 | 43.1 | 57.86 | 150 |
|  | GE005104 | *pilQ* | type IV pilus biogenesis protein PilQ | Type IV pili | 1.54E-46 | 45.86 | 92.61 | 158 |
|  | GE005105 | *pilR* | type IV pilus biosynthesis protein PilR | Type IV pili | 1.61E-38 | 30.5 | 84.01 | 140 |
|  | GE005106 | *pilS* | putative type IV pilus prepilin | Type IV pili | 1.39E-10 | 29.65 | 91.35 | 54 |
|  | GE005107 | *ipgF* | type III secretion system protein IpgF | TTSS | 8.06E-22 | 36.51 | 78.12 | 85 |
|  | GE005108 | *tapD* | prepilin peptidase, pilD-like | Tap type IV pili | 3.41E-07 | 30.58 | 54.03 | 46 |
|  | GE005109 | *pilV* | type IV pilus biogenesis protein PilV | Type IV pili | 1.78E-88 | 41.34 | 81.31 | 276 |
|  | GE005138 | *trwD* | Trw type IV secretion sysmtem VirB11 homolog, trwD protein | Trw type IV secretion system | 2.31E-70 | 38.36 | 92.94 | 223 |
|  | GE005139 | *virD4* | type IV secretion system component VirD4 | Rvh T4SS | 2.62E-59 | 28.62 | 89.11 | 211 |
|  | GE005141 | *pilN* | type IV pilus inner membrane platform protein PilN | Type IV pili | 2.24E-108 | 38.28 | 97.26 | 336 |
|  | GE005142 | *pilO* | type IV pilus inner membrane platform protein PilO | Type IV pili | 1.64E-16 | 20.82 | 95.19 | 78 |
|  | GE005143 | *pilP* | type IV pilus biogenesis protein PilP | Type IV pili | 1.16E-09 | 29.27 | 71.35 | 51 |
|  | GE005144 | *pilQ* | type IV pilus biogenesis protein PilQ | Type IV pili | 8.86E-28 | 32.99 | 78.78 | 108 |
|  | GE005145 | *pilQ* | type IV pilus biogenesis protein PilQ | Type IV pili | 1.96E-69 | 48.41 | 93.42 | 222 |
|  | GE005146 | *pilR* | type IV pilus biosynthesis protein PilR | Type IV pili | 3.75E-38 | 30.77 | 91.02 | 138 |
|  | GE005147 | *pilS* | putative type IV pilus prepilin | Type IV pili | 1.39E-10 | 29.65 | 91.35 | 54 |
|  | GE005148 | *ipgF* | type III secretion system protein IpgF | TTSS | 8.06E-22 | 36.51 | 78.12 | 85 |
|  | GE005149 | *tapD* | prepilin peptidase, pilD-like | Tap type IV pili | 3.41E-07 | 30.58 | 54.03 | 46 |
|  | GE005150 | *pilV* | type IV pilus biogenesis protein PilV | Type IV pili | 1.78E-88 | 41.34 | 81.31 | 276 |

Table S3 Comparative pathogenomics of *K. pneumoniae* strains.

| **Virulence factors** | **Related genes** | **Strain 1084** | **Strain HS11286** | **Strain MGH 78578** | **Strain NTUH-K2044** | | **Strain HKE9** | |
| --- | --- | --- | --- | --- | --- | --- | --- | --- |
|  |  | **chromosome NC_018522** | **chromosome NC_016845** | **chromosome NC_009648** | **chromosome NC_012731** | **pK2044 NC_006625** | **chromosome** | **plasmid** |
| ***Adherence*** | | | | | | | | |
| Type I fimbriae | *fimB* | + | + | + | + | - | + | - |
|  | *fimE* | + | + | + | + | - | + | - |
|  | *fimA* | + | + | + | + | - | + | - |
|  | *fimI* | + | + | + | + | - | + | - |
|  | *fimC* | + | + | + | + | - | + | - |
|  | *fimD* | + | + | + | + | - | + | - |
|  | *fimF* | + | + | + | + | - | + | - |
|  | *fimG* | + | + | + | + | - | + | - |
|  | *fimH* | + | + | + | + | - | + | - |
|  | *fimK* | + | + | + | + | - | + | - |
| ***Effector delivery system*** | | | | | | | | |
| T6SS | *vipA/tssB* | + | + | + | + | - | + | - |
|  | *vipB/tssC* | + | + | + | + | - | + | - |
|  | *vasE/tssK* | + | + | + | + | - | + | - |
|  | *dotU/tssL* | + | + | + | + | - | + | - |
|  | *ompA* | + | + | + | + | - | + | - |
|  | *hcp/tssD* | + | + | + | + | - | + | - |
|  | *clpV/tssH* | + | + | + | + | - | + | - |
|  | *vgrG/tssI* | + | + | + | + | - | + | - |
|  | *tli1* | - | + | - | - | - | - | - |
|  | *tle1* | - | + | - | - | - | + | - |
|  | *icmF/tssM* | + | + | + | + | - | + | - |
|  | *impA/tssA* | + | + | + | + | - | + | - |
|  | *tssF* | + | + | + | + | - | + | - |
|  | *tssG* | + | + | - | + | - | + | - |
|  | *sciN/tssJ* | + | + | - | + | - | + | - |
| T6SS-II | *impF* | - | - | + | - | - | - | - |
|  | *sciN* | - | - | - | - | - | - | - |
|  | *impH* | - | - | + | - | - | - | - |
|  | *vasA/impG* | - | - | + | - | - | - | - |
|  | *icmF* | - | - | + | - | - | - | - |
|  | *vgrG* | - | - | + | - | - | - | - |
|  | *ompA* | - | - | + | - | - | - | - |
|  | *dotU* | - | - | - | - | - | - | - |
|  | *impJ* | - | - | + | - | - | - | - |
|  | *clpV* | + | + | + | + | - | - | - |
| T6SS-III | *impJ* | + | + | + | + | - | - | - |
|  | *dotU* | + | + | - | + | - | + | - |
|  | *ompA* | + | + | + | + | - | + | - |
|  | *vgrG* | + | + | + | + | - | + | - |
|  | *lysM* | - | - | - | - | - | - | - |
|  | *icmF* | + | + | + | + | - | + | - |
|  | *impG* | + | + | + | + | - | - | - |
|  | *impH* | + | + | + | + | - | - | - |
|  | *sciN* | + | + | + | + | - | + | - |
|  | *impF* | + | + | - | + | - | - | - |
|  | *impA* | + | + | + | + | - | - | - |
| ***Exotoxin*** | | | | | | | | |
| Colibactin | *clbA* | + | - | - | - | - | - | - |
|  | *clbB* | + | - | - | - | - | + | - |
|  | *clbC* | + | - | - | - | - | - | - |
|  | *clbD* | + | - | - | - | - | + | - |
|  | *clbE* | + | - | - | - | - | - | - |
|  | *clbF* | + | - | - | - | - | + | - |
|  | *clbG* | + | - | - | - | - | + | - |
|  | *clbH* | + | - | - | - | - | - | - |
|  | *clbI* | + | - | - | - | - | + | - |
|  | *clbJ* | + | - | - | - | - | - | - |
|  | *clbK* | + | - | - | - | - | - | - |
|  | *clbL* | + | - | - | - | - | + | - |
|  | *clbM* | + | - | - | - | - | + | - |
|  | *clbN* | + | - | - | - | - | - | - |
|  | *clbO* | + | - | - | - | - | - | - |
|  | *clbP* | + | - | - | - | - | + | - |
|  | *clbQ* | + | - | - | - | - | - | - |
|  | *clbS* | + | - | - | - | - | - | - |
| ***Immune modulation*** | | | | | | | | |
| Capsule | - | + | + | + | + | - | + | - |
| LPS | - | + | + | + | + | - | + | - |
| ***Biofilm*** | | | | | | | | |
| Type 3 fimbriae | *mrkH* | + | + | - | + | - | + | - |
|  | *mrkI* | + | + | + | + | - | + | - |
|  | *mrkJ* | + | + | + | + | - | + | - |
|  | *mrkF* | + | + | + | + | - | + | - |
|  | *mrkD* | + | + | + | + | - | + | - |
|  | *mrkC* | + | + | + | + | - | + | - |
|  | *mrkB* | + | + | + | + | - | + | - |
|  | *mrkA* | + | + | + | + | - | + | - |
| ***Nutritional/Metabolic factor*** | | | | | | | | |
| Aerobactin | *iucA* | - | - | - | - | + | - | + |
|  | *iucB* | - | - | - | - | + | - | + |
|  | *iucC* | - | - | - | - | + | - | + |
|  | *iucD* | - | - | - | - | + | - | + |
|  | *iutA* | + | + | + | + | + | + | + |
| Allantoin utilization | *allS* | + | - | - | + | - | + | - |
|  | *allA* | + | - | - | + | - |  | - |
|  | *allR* | + | - | - | + | - | + | - |
|  | *allB* | + | - | - | + | - |  | - |
|  | *allC* | + | - | - | + | - | + | - |
|  | *allD* | + | - | - | + | - | + | - |
| Ent | *entA* | + | + | + | + | - | + | - |
|  | *entB* | + | + | + | + | - | + | - |
|  | *entE* | + | + | + | + | - | + | - |
|  | *entC* | + | + | + | + | - | + | - |
|  | *fepB* | + | + | + | + | - | + | - |
|  | *entS* | + | + | + | + | - | - | - |
|  | *fepD* | + | + | + | + | - | + | - |
|  | *fepG* | + | + | + | + | - | + | - |
|  | *fepC* | + | + | + | + | - | + | - |
|  | *entF* | + | + | + | + | - | + | - |
|  | *fes* | + | + | + | + | - | + | - |
|  | *fepA* | + | + | + | + | - | + | - |
|  | *entD* | + | + | + | + | - | + | - |
| Sal | *iroE* | + | + | + | + | - | + | - |
|  | *iroN* | + | + | + | + | + | + | + |
|  | *iroB* | + | - | - | + | + | - | + |
|  | *iroC* | - | - | - | + | + | - | + |
|  | *iroD* | + | - | - | + | + | - | + |
| Ybt | *ybtS* | + | + | - | + | - | + | - |
|  | *ybtX* | + | + | - | + | - | + | - |
|  | *ybtQ* | + | + | - | + | - | + | - |
|  | *ybtP* | + | + | - | + | - | + | - |
|  | *ybtA* | + | + | - | + | - | + | - |
|  | *irp2* | + | + | - | + | - | + | - |
|  | *irp1* | + | + | - | + | - | + | - |
|  | *ybtU* | + | + | - | + | - | + | - |
|  | *ybtT* | + | + | - | + | - | + | - |
|  | *ybtE* | + | + | - | + | - | + | - |
|  | *fyuA* | + | + | - | + | - | + | - |
| ***Antimicrobial activity/Competitive advantage*** | | | | | | | | |
| AcrAB | *acrA* | + | + | + | + | - | + | - |
|  | *acrB* | + | + | + | + | - | + | - |
| ***Regulation*** | | | | | | | | |
| RcsAB | *rcsA* | + | + | + | + | - | + | - |
|  | *rcsB* | + | + | + | + | - | + | - |
| RmpA | *rmpA* | - | - | - | + | + | - | - |
|  | *rmpA2* | - | - | - | - | - | - | - |

**Table S4** **The DEGs of HKE9 vs HKE9-M-*rpoS* with FDR < 0.05 and | log2FC | >2.**

| id | Symbol | log2(FC) | 2^-ΔΔT^ | Pvalue | FDR | Description |
| --- | --- | --- | --- | --- | --- | --- |
| GE003776 | *oadG* | 12.77 | 6973.33 | 0.01 | 0.04 | Oxaloacetate decarboxylase gamma chain |
| GE000085 | *oadA* | 5.98 | 63.33 | 0.00 | 0.01 | Oxaloacetate decarboxylase alpha chain |
| GE000458 | *--* | 4.47 | 22.19 | 0.00 | 0.01 | hypothetical protein |
| GE000237 | *--* | 3.83 | 14.26 | 0.01 | 0.04 | putative 15.0 kDa protein in dhaT-dhaS intergenic region |
| GE003517 | *ecpB* | 3.23 | 9.41 | 0.00 | 0.00 | putative fimbrial chaperone EcpB |
| GE001935 | *catA* | 2.67 | 6.37 | 0.00 | 0.00 | Catechol 1,2-dioxygenase |
| GE002041 | *asd* | 2.65 | 6.26 | 0.00 | 0.00 | Aspartate-semialdehyde dehydrogenase |
| GE003518 | *ecpA* | 2.58 | 5.98 | 0.00 | 0.00 | Common pilus major fimbrillin subunit EcpA |
| GE001486 | *tdcE* | 2.53 | 5.79 | 0.00 | 0.00 | PFL-like enzyme TdcE |
| GE003807 | *thrA* | 2.45 | 5.45 | 0.00 | 0.00 | Bifunctional aspartokinase/homoserine dehydrogenase 1 |
| GE004697 | *lldD* | 2.41 | 5.32 | 0.00 | 0.00 | L-lactate dehydrogenase |
| GE004032 | *argI* | 2.37 | 5.19 | 0.00 | 0.00 | Ornithine carbamoyltransferase |
| GE002311 | *ydgI* | 2.33 | 5.03 | 0.01 | 0.04 | Putative arginine/ornithine antiporter |
| GE000487 | *fim* | 2.31 | 4.97 | 0.00 | 0.00 | Fimbrial subunit type 1 |
| GE003519 | *ecpR* | 2.18 | 4.52 | 0.01 | 0.04 | HTH-type transcriptional regulator EcpR |
| GE001691 | *--* | 2.16 | 4.47 | 0.00 | 0.00 | hypothetical protein |
| GE002167 | *metQ* | 2.14 | 4.42 | 0.00 | 0.01 | putative D-methionine-binding lipoprotein MetQ |
| GE002202 | *sotB* | 2.12 | 4.33 | 0.00 | 0.00 | putative sugar efflux transporter |
| GE004699 | *lldP* | 2.07 | 4.21 | 0.01 | 0.05 | L-lactate permease |
| GE000486 | *fimI* | 2.05 | 4.13 | 0.00 | 0.00 | Fimbrin-like protein FimI |
| GE001484 | *tdcC* | 2.04 | 4.12 | 0.00 | 0.00 | Threonine/serine transporter TdcC |
| GE001541 | *chaB* | -2.00 | 0.25 | 0.00 | 0.00 | Putative cation transport regulator ChaB |
| GE001968 | *treS* | -2.00 | 0.25 | 0.00 | 0.00 | Trehalose synthase |
| GE000592 | *cobQ* | -2.00 | 0.25 | 0.00 | 0.00 | Cobyric acid synthase |
| GE002877 | *ycaP* | -2.03 | 0.25 | 0.00 | 0.00 | UPF0702 transmembrane protein YcaP |
| GE001635 | *sufE* | -2.03 | 0.24 | 0.00 | 0.00 | Cysteine desulfuration protein SufE |
| GE000579 | *cbiT* | -2.04 | 0.24 | 0.00 | 0.00 | Cobalt-precorrin-6B C(15)-methyltransferase (decarboxylating) |
| GE001631 | *sufB* | -2.04 | 0.24 | 0.00 | 0.00 | FeS cluster assembly protein SufB |
| GE004106 | *bsmA* | -2.04 | 0.24 | 0.00 | 0.00 | Lipoprotein BsmA |
| GE000585 | *cbiJ* | -2.05 | 0.24 | 0.00 | 0.00 | Cobalt-precorrin-6A reductase |
| GE000196 | *yqjG* | -2.05 | 0.24 | 0.00 | 0.00 | Glutathionyl-hydroquinone reductase PcpF |
| GE000593 | *cobQ* | -2.05 | 0.24 | 0.00 | 0.00 | Cobyric acid synthase |
| GE001232 | *--* | -2.06 | 0.24 | 0.00 | 0.00 | hypothetical protein |
| GE000954 | *eutR* | -2.06 | 0.24 | 0.00 | 0.00 | HTH-type transcriptional regulator EutR |
| GE001630 | *sufA* | -2.07 | 0.24 | 0.00 | 0.00 | Protein SufA |
| GE002551 | *astE* | -2.07 | 0.24 | 0.00 | 0.00 | Succinylglutamate desuccinylase |
| GE001632 | *sufC* | -2.07 | 0.24 | 0.00 | 0.00 | putative ATP-dependent transporter SufC |
| GE000588 | *cbiM* | -2.07 | 0.24 | 0.00 | 0.00 | Cobalt transport protein CbiM |
| GE005051 | *ybcY* | -2.09 | 0.24 | 0.00 | 0.00 | Putative uncharacterized protein YbcY |
| GE004748 | *yhjY* | -2.11 | 0.23 | 0.00 | 0.00 | putative protein YhjY |
| GE001969 | *sadH* | -2.12 | 0.23 | 0.00 | 0.00 | Putative oxidoreductase SadH |
| GE001719 | *cycH* | -2.13 | 0.23 | 0.00 | 0.00 | Cytochrome c-type biogenesis protein CycH |
| GE001634 | *sufS* | -2.13 | 0.23 | 0.00 | 0.00 | Cysteine desulfurase |
| GE003038 | *ariR* | -2.14 | 0.23 | 0.00 | 0.00 | Regulatory protein AriR |
| GE004079 | *ytfK* | -2.15 | 0.22 | 0.00 | 0.00 | hypothetical protein |
| GE002020 | *--* | -2.16 | 0.22 | 0.00 | 0.00 | hypothetical protein |
| GE001302 | *erfK* | -2.17 | 0.22 | 0.00 | 0.00 | putative L,D-transpeptidase ErfK/SrfK |
| GE001954 | *adhP* | -2.18 | 0.22 | 0.00 | 0.00 | Alcohol dehydrogenase, propanol-preferring |
| GE003025 | *ctpF* | -2.18 | 0.22 | 0.00 | 0.00 | putative cation-transporting ATPase F |
| GE000590 | *cbiQ* | -2.19 | 0.22 | 0.00 | 0.00 | Cobalt transport protein CbiQ |
| GE000595 | *cobT* | -2.20 | 0.22 | 0.00 | 0.00 | Nicotinate-nucleotide--dimethylbenzimidazole phosphoribosyltransferase |
| GE003502 | *eutB* | -2.21 | 0.22 | 0.00 | 0.00 | Ethanolamine ammonia-lyase heavy chain |
| GE002547 | *osmE* | -2.23 | 0.21 | 0.00 | 0.00 | hypothetical protein |
| GE004550 | *ibpB* | -2.23 | 0.21 | 0.00 | 0.00 | Small heat shock protein IbpB |
| GE002552 | *astB* | -2.24 | 0.21 | 0.00 | 0.00 | N-succinylarginine dihydrolase |
| GE001286 | *dosC* | -2.26 | 0.21 | 0.00 | 0.00 | Diguanylate cyclase DosC |
| GE001884 | *ortT* | -2.29 | 0.21 | 0.00 | 0.01 | Orphan toxin OrtT |
| GE000950 | *eutB* | -2.29 | 0.20 | 0.00 | 0.00 | Ethanolamine ammonia-lyase heavy chain |
| GE003936 | *--* | -2.29 | 0.20 | 0.00 | 0.02 | hypothetical protein |
| GE004302 | *thiF* | -2.29 | 0.20 | 0.00 | 0.00 | Sulfur carrier protein ThiS adenylyltransferase |
| GE001633 | *sufD* | -2.30 | 0.20 | 0.00 | 0.00 | FeS cluster assembly protein SufD |
| GE001382 | *otsA* | -2.30 | 0.20 | 0.00 | 0.00 | Trehalose-6-phosphate synthase |
| GE003558 | *gabT* | -2.33 | 0.20 | 0.00 | 0.00 | 4-aminobutyrate aminotransferase GabT |
| GE004171 | *cbpA* | -2.33 | 0.20 | 0.00 | 0.00 | Curved DNA-binding protein |
| GE002248 | *lgoT* | -2.34 | 0.20 | 0.00 | 0.00 | putative L-galactonate transporter |
| GE000199 | *yqjE* | -2.34 | 0.20 | 0.00 | 0.00 | hypothetical protein |
| GE000594 | *cobU* | -2.36 | 0.20 | 0.00 | 0.00 | Bifunctional adenosylcobalamin biosynthesis protein CobU |
| GE004701 | *yibT* | -2.36 | 0.19 | 0.00 | 0.00 | putative protein YibT |
| GE001201 | *gci* | -2.37 | 0.19 | 0.00 | 0.00 | D-galactarolactone cycloisomerase |
| GE003036 | *ycgZ* | -2.37 | 0.19 | 0.00 | 0.00 | putative two-component-system connector protein YcgZ |
| GE000577 | *cbiD* | -2.38 | 0.19 | 0.00 | 0.00 | Cobalt-precorrin-5B C(1)-methyltransferase |
| GE002057 | *namA* | -2.38 | 0.19 | 0.00 | 0.00 | NADPH dehydrogenase |
| GE004173 | *cbpM* | -2.41 | 0.19 | 0.00 | 0.00 | Chaperone modulatory protein CbpM |
| GE002442 | *--* | -2.41 | 0.19 | 0.00 | 0.00 | hypothetical protein |
| GE000661 | *yhcA* | -2.42 | 0.19 | 0.00 | 0.00 | putative MFS-type transporter YhcA |
| GE004107 | *yjfN* | -2.43 | 0.19 | 0.00 | 0.00 | hypothetical protein |
| GE001629 | *--* | -2.44 | 0.18 | 0.00 | 0.00 | hypothetical protein |
| GE004905 | *fic* | -2.44 | 0.18 | 0.00 | 0.00 | putative protein adenylyltransferase Fic |
| GE002058 | *ydhS* | -2.45 | 0.18 | 0.00 | 0.00 | putative protein YdhS |
| GE001861 | *ydcK* | -2.46 | 0.18 | 0.00 | 0.00 | putative acetyltransferase YdcK |
| GE002373 | *feaB* | -2.46 | 0.18 | 0.00 | 0.00 | Phenylacetaldehyde dehydrogenase |
| GE002444 | *bcsA* | -2.47 | 0.18 | 0.00 | 0.00 | Cellulose synthase catalytic subunit |
| GE004756 | *dppF* | -2.47 | 0.18 | 0.00 | 0.00 | Dipeptide transport ATP-binding protein DppF |
| GE000201 | *yqjC* | -2.48 | 0.18 | 0.00 | 0.00 | hypothetical protein |
| GE003037 | *--* | -2.49 | 0.18 | 0.00 | 0.00 | hypothetical protein |
| GE000578 | *cbiE* | -2.50 | 0.18 | 0.00 | 0.00 | Cobalt-precorrin-7 C(5)-methyltransferase |
| GE002441 | *pleD* | -2.50 | 0.18 | 0.00 | 0.00 | Response regulator PleD |
| GE002805 | *pdeG* | -2.51 | 0.18 | 0.00 | 0.00 | putative cyclic di-GMP phosphodiesterase PdeG |
| GE000576 | *cbiC* | -2.54 | 0.17 | 0.00 | 0.00 | Cobalt-precorrin-8 methylmutase |
| GE004752 | *dppA* | -2.54 | 0.17 | 0.00 | 0.00 | Periplasmic dipeptide transport protein |
| GE004068 | *ytfT* | -2.56 | 0.17 | 0.00 | 0.00 | Fructose import permease protein FruF |
| GE002443 | *--* | -2.56 | 0.17 | 0.00 | 0.00 | hypothetical protein |
| GE002003 | *DPEP1* | -2.57 | 0.17 | 0.00 | 0.00 | hypothetical protein |
| GE004095 | *yjfY* | -2.58 | 0.17 | 0.00 | 0.00 | hypothetical protein |
| GE000200 | *yqjD* | -2.58 | 0.17 | 0.00 | 0.00 | Protein ElaB |
| GE003029 | *nolG* | -2.58 | 0.17 | 0.00 | 0.00 | Nodulation protein NolG |
| GE004705 | *yibF* | -2.58 | 0.17 | 0.00 | 0.00 | putative GST-like protein YibF |
| GE000589 | *cbiN* | -2.59 | 0.17 | 0.00 | 0.00 | Cobalt transport protein CbiN |
| GE002449 | *dhkJ* | -2.60 | 0.16 | 0.00 | 0.00 | Autoinducer 2 sensor kinase/phosphatase LuxQ |
| GE003557 | *gabD* | -2.63 | 0.16 | 0.00 | 0.00 | Succinate-semialdehyde dehydrogenase GabD |
| GE004754 | *dppC* | -2.63 | 0.16 | 0.00 | 0.00 | Dipeptide transport system permease protein DppC |
| GE002725 | *msyB* | -2.64 | 0.16 | 0.00 | 0.00 | Acidic protein MsyB |
| GE004753 | *dppB* | -2.65 | 0.16 | 0.00 | 0.00 | Dipeptide transport system permease protein DppB |
| GE001381 | *otsB* | -2.65 | 0.16 | 0.00 | 0.00 | Trehalose-phosphate phosphatase |
| GE004854 | *glgX* | -2.65 | 0.16 | 0.00 | 0.00 | Glycogen debranching enzyme |
| GE001612 | *yeaQ* | -2.66 | 0.16 | 0.00 | 0.00 | UPF0410 protein YeaQ |
| GE001421 | *pphA* | -2.66 | 0.16 | 0.00 | 0.00 | Serine/threonine-protein phosphatase 1 |
| GE002997 | *clsB* | -2.66 | 0.16 | 0.00 | 0.00 | Cardiolipin synthase B |
| GE003559 | *gabP* | -2.67 | 0.16 | 0.00 | 0.00 | GABA permease |
| GE003937 | *MXAN_5909* | -2.68 | 0.16 | 0.00 | 0.01 | putative oxidoreductase MXAN_5909 |
| GE003026 | *uspG* | -2.70 | 0.15 | 0.00 | 0.00 | Universal stress protein G |
| GE000225 | *patA* | -2.70 | 0.15 | 0.00 | 0.00 | Putrescine aminotransferase |
| GE002450 | *NIK1* | -2.71 | 0.15 | 0.00 | 0.00 | Sensor histidine kinase GacS |
| GE004172 | *cbpA* | -2.72 | 0.15 | 0.00 | 0.00 | Curved DNA-binding protein |
| GE003028 | *mdtA* | -2.73 | 0.15 | 0.00 | 0.00 | Multidrug resistance protein MdtA |
| GE000574 | *cbiA* | -2.74 | 0.15 | 0.00 | 0.00 | Cobyrinate a,c-diamide synthase |
| GE000198 | *yqjK* | -2.75 | 0.15 | 0.00 | 0.00 | hypothetical protein |
| GE003389 | *ybaY* | -2.81 | 0.14 | 0.00 | 0.00 | putative lipoprotein YbaY |
| GE001161 | *yehW* | -2.82 | 0.14 | 0.00 | 0.00 | Glycine betaine uptake system permease protein YehW |
| GE001285 | *cpo* | -2.82 | 0.14 | 0.00 | 0.00 | Non-heme chloroperoxidase |
| GE000943 | *eutM* | -2.86 | 0.14 | 0.00 | 0.00 | Ethanolamine utilization protein EutM |
| GE004755 | *dppD* | -2.87 | 0.14 | 0.00 | 0.00 | Dipeptide transport ATP-binding protein DppD |
| GE001720 | *--* | -2.88 | 0.14 | 0.01 | 0.05 | hypothetical protein |
| GE002451 | *mndB* | -2.89 | 0.14 | 0.00 | 0.00 | hypothetical protein |
| GE001945 | *pkk2A* | -2.90 | 0.13 | 0.00 | 0.00 | putative polyphosphate kinase PKK2A |
| GE002980 | *ybiI* | -2.92 | 0.13 | 0.00 | 0.00 | putative protein YbiI |
| GE002447 | *--* | -2.92 | 0.13 | 0.00 | 0.00 | hypothetical protein |
| GE000575 | *cobD* | -2.93 | 0.13 | 0.00 | 0.00 | Cobalamin biosynthesis protein CobD |
| GE003939 | *--* | -2.93 | 0.13 | 0.01 | 0.03 | hypothetical protein |
| GE002448 | *--* | -2.95 | 0.13 | 0.00 | 0.00 | hypothetical protein |
| GE002625 | *cotJC* | -2.96 | 0.13 | 0.00 | 0.00 | Protein CotJC |
| GE002240 | *yedK* | -2.98 | 0.13 | 0.00 | 0.00 | SOS response-associated protein YedK |
| GE002289 | *--* | -3.01 | 0.12 | 0.00 | 0.00 | hypothetical protein |
| GE002806 | *--* | -3.05 | 0.12 | 0.00 | 0.00 | hypothetical protein |
| GE001878 | *ydcT* | -3.06 | 0.12 | 0.00 | 0.00 | putative ABC transporter ATP-binding protein YdcT |
| GE003692 | *aroP* | -3.07 | 0.12 | 0.00 | 0.00 | Aromatic amino acid transport protein AroP |
| GE001881 | *patD* | -3.10 | 0.12 | 0.00 | 0.00 | Gamma-aminobutyraldehyde dehydrogenase |
| GE002445 | *--* | -3.15 | 0.11 | 0.00 | 0.00 | hypothetical protein |
| GE003313 | *--* | -3.18 | 0.11 | 0.00 | 0.00 | hypothetical protein |
| GE001160 | *yehX* | -3.19 | 0.11 | 0.00 | 0.00 | Glycine betaine uptake system ATP-binding protein YehX |
| GE001035 | *yfcG* | -3.19 | 0.11 | 0.00 | 0.00 | Disulfide-bond oxidoreductase YfcG |
| GE004576 | *--* | -3.20 | 0.11 | 0.00 | 0.00 | hypothetical protein |
| GE001158 | *yehZ* | -3.21 | 0.11 | 0.00 | 0.00 | Glycine betaine-binding protein YehZ |
| GE002976 | *ybiO* | -3.21 | 0.11 | 0.00 | 0.00 | Moderate conductance mechanosensitive channel YbiO |
| GE000005 | *bfr* | -3.22 | 0.11 | 0.00 | 0.00 | Bacterioferritin |
| GE001474 | *treA* | -3.23 | 0.11 | 0.00 | 0.00 | Periplasmic trehalase |
| GE003244 | *KPN78578_05520* | -3.23 | 0.11 | 0.00 | 0.00 | Putative glutamate--cysteine ligase 2 |
| GE003302 | *--* | -3.24 | 0.11 | 0.00 | 0.00 | hypothetical protein |
| GE003556 | *lhgD* | -3.26 | 0.10 | 0.00 | 0.00 | L-2-hydroxyglutarate dehydrogenase |
| GE004853 | *glgB* | -3.26 | 0.10 | 0.00 | 0.00 | 1,4-alpha-glucan branching enzyme GlgB |
| GE004096 | *MT2327* | -3.27 | 0.10 | 0.00 | 0.00 | putative protein MT2327 |
| GE001159 | *yehY* | -3.27 | 0.10 | 0.00 | 0.00 | Glycine betaine uptake system permease protein YehY |
| GE004575 | *--* | -3.27 | 0.10 | 0.00 | 0.01 | hypothetical protein |
| GE004870 | *--* | -3.28 | 0.10 | 0.00 | 0.00 | hypothetical protein |
| GE002446 | *--* | -3.31 | 0.10 | 0.00 | 0.00 | hypothetical protein |
| GE000935 | *tktB* | -3.38 | 0.10 | 0.00 | 0.00 | Transketolase 2 |
| GE003183 | *fabG* | -3.38 | 0.10 | 0.00 | 0.00 | 3-oxoacyl-[acyl-carrier-protein] reductase FabG |
| GE002972 | *dps* | -3.42 | 0.09 | 0.00 | 0.00 | DNA protection during starvation protein |
| GE001153 | *yohC* | -3.44 | 0.09 | 0.00 | 0.00 | hypothetical protein |
| GE004777 | *yhjG* | -3.45 | 0.09 | 0.00 | 0.00 | AsmA family protein YhjG |
| GE002807 | *glgP* | -3.50 | 0.09 | 0.00 | 0.00 | Glycogen phosphorylase |
| GE004844 | *ggt* | -3.52 | 0.09 | 0.00 | 0.00 | Glutathione hydrolase proenzyme |
| GE004736 | *yiaG* | -3.54 | 0.09 | 0.00 | 0.00 | putative HTH-type transcriptional regulator YiaG |
| GE002090 | *kbp* | -3.55 | 0.09 | 0.00 | 0.00 | Potassium binding protein Kbp |
| GE001816 | *sodC* | -3.56 | 0.08 | 0.00 | 0.00 | Superoxide dismutase |
| GE004812 | *yhhT* | -3.56 | 0.08 | 0.00 | 0.00 | Putative transport protein YhhT |
| GE002627 | *yciF* | -3.57 | 0.08 | 0.00 | 0.00 | Protein YciF |
| GE003033 | *--* | -3.58 | 0.08 | 0.00 | 0.00 | hypothetical protein |
| GE001877 | *ydcS* | -3.58 | 0.08 | 0.00 | 0.00 | Bifunctional polyhydroxybutyrate synthase / ABC transporter periplasmic binding protein |
| GE004183 | *yjdN* | -3.58 | 0.08 | 0.00 | 0.00 | hypothetical protein |
| GE000600 | *ygdI* | -3.60 | 0.08 | 0.00 | 0.00 | putative lipoprotein YgdI |
| GE001925 | *narV* | -3.61 | 0.08 | 0.00 | 0.00 | Respiratory nitrate reductase 2 gamma chain |
| GE000781 | *yvaG* | -3.66 | 0.08 | 0.00 | 0.00 | Nodulation protein G |
| GE004137 | *blc* | -3.71 | 0.08 | 0.00 | 0.00 | Outer membrane lipoprotein Blc |
| GE002587 | *KPN78578_11640* | -3.71 | 0.08 | 0.00 | 0.00 | UPF0229 protein KPN78578_11640 |
| GE000988 | *ipdC* | -3.71 | 0.08 | 0.00 | 0.00 | Indole-3-pyruvate decarboxylase |
| GE002626 | *yciE* | -3.72 | 0.08 | 0.00 | 0.00 | Protein YciE |
| GE003053 | *ybgS* | -3.72 | 0.08 | 0.00 | 0.00 | putative protein YbgS |
| GE000936 | *talA* | -3.75 | 0.07 | 0.00 | 0.00 | Transaldolase A |
| GE001856 | *ydcJ* | -3.75 | 0.07 | 0.00 | 0.00 | putative protein YdcJ |
| GE003503 | *eutC* | -3.81 | 0.07 | 0.00 | 0.00 | Ethanolamine ammonia-lyase light chain |
| GE000949 | *eutA* | -3.81 | 0.07 | 0.00 | 0.00 | Ethanolamine utilization protein EutA |
| GE004619 | *yahK* | -3.89 | 0.07 | 0.00 | 0.00 | Aldehyde reductase YahK |
| GE001423 | *yebV* | -3.91 | 0.07 | 0.00 | 0.00 | putative protein YebV |
| GE003897 | *ssdA* | -3.93 | 0.07 | 0.00 | 0.00 | Succinate-semialdehyde dehydrogenase |
| GE002173 | *yjiJ* | -3.96 | 0.06 | 0.00 | 0.01 | hypothetical protein |
| GE001880 | *ydcV* | -3.97 | 0.06 | 0.00 | 0.00 | Inner membrane ABC transporter permease protein YdcV |
| GE000074 | *yhcO* | -3.98 | 0.06 | 0.00 | 0.00 | putative protein YhcO |
| GE001964 | *yhxD* | -4.07 | 0.06 | 0.00 | 0.00 | putative oxidoreductase YhxD |
| GE000944 | *eutN* | -4.10 | 0.06 | 0.00 | 0.00 | Ethanolamine utilization protein EutN |
| GE000946 | *eutJ* | -4.11 | 0.06 | 0.00 | 0.00 | Ethanolamine utilization protein EutJ |
| GE000945 | *eutE* | -4.13 | 0.06 | 0.00 | 0.00 | Ethanolamine utilization protein EutE |
| GE000218 | *lsrR* | -4.16 | 0.06 | 0.00 | 0.00 | Transcriptional regulator LsrR |
| GE001927 | *narY* | -4.22 | 0.05 | 0.00 | 0.00 | Respiratory nitrate reductase 2 beta chain |
| GE001354 | *yodD* | -4.34 | 0.05 | 0.00 | 0.00 | hypothetical protein |
| GE000223 | *lsrF* | -4.39 | 0.05 | 0.00 | 0.00 | 3-hydroxy-5-phosphonooxypentane-2,4-dione thiolase |
| GE000948 | *eutH* | -4.41 | 0.05 | 0.00 | 0.00 | Ethanolamine utilization protein EutH |
| GE001926 | *narW* | -4.42 | 0.05 | 0.00 | 0.00 | putative nitrate reductase molybdenum cofactor assembly chaperone NarW |
| GE002319 | *fumC* | -4.47 | 0.05 | 0.00 | 0.00 | Fumarate hydratase class II |
| GE000224 | *lsrG* | -4.48 | 0.04 | 0.00 | 0.00 | (4S)-4-hydroxy-5-phosphonooxypentane-2,3-dione isomerase |
| GE001879 | *ydcU* | -4.52 | 0.04 | 0.00 | 0.00 | Inner membrane ABC transporter permease protein YdcU |
| GE001977 | *--* | -4.55 | 0.04 | 0.00 | 0.00 | hypothetical protein |
| GE000947 | *eutG* | -4.56 | 0.04 | 0.00 | 0.00 | Ethanolamine utilization protein EutG |
| GE001467 | *ycgB* | -4.59 | 0.04 | 0.00 | 0.00 | putative protein YcgB |
| GE001929 | *narU* | -4.61 | 0.04 | 0.00 | 0.00 | Nitrate/nitrite transporter NarU |
| GE003475 | *yaiA* | -4.63 | 0.04 | 0.00 | 0.00 | putative protein YaiA |
| GE002628 | *yciG* | -4.65 | 0.04 | 0.00 | 0.00 | putative protein YciG |
| GE001191 | *fbaB* | -4.67 | 0.04 | 0.00 | 0.00 | Fructose-bisphosphate aldolase class 1 |
| GE001973 | *ydeI* | -4.69 | 0.04 | 0.00 | 0.00 | putative protein YdeI |
| GE000217 | *lsrK* | -4.79 | 0.04 | 0.00 | 0.00 | Autoinducer-2 kinase |
| GE004193 | *phnP* | -4.83 | 0.04 | 0.00 | 0.00 | Phosphoribosyl 1,2-cyclic phosphate phosphodiesterase |
| GE000572 | *pduF* | -4.89 | 0.03 | 0.00 | 0.00 | Propanediol diffusion facilitator |
| GE003480 | *psiF* | -4.91 | 0.03 | 0.00 | 0.00 | Phosphate starvation-inducible protein PsiF |
| GE000866 | *csiE* | -4.97 | 0.03 | 0.00 | 0.00 | Stationary phase-inducible protein CsiE |
| GE000552 | *pduW* | -4.98 | 0.03 | 0.00 | 0.00 | putative propionate kinase |
| GE001975 | *osmC* | -4.99 | 0.03 | 0.00 | 0.00 | Peroxiredoxin OsmC |
| GE000556 | *rnfC* | -5.05 | 0.03 | 0.00 | 0.00 | Na(+)-translocating ferredoxin:NAD(+) oxidoreductase complex subunit C |
| GE001928 | *narZ* | -5.06 | 0.03 | 0.00 | 0.00 | Respiratory nitrate reductase 2 alpha chain |
| GE002540 | *katE* | -5.13 | 0.03 | 0.00 | 0.00 | Catalase HPII |
| GE003832 | *KPK_4780* | -5.35 | 0.02 | 0.00 | 0.00 | UPF0391 membrane protein KPK_4780 |
| GE000222 | *lsrB* | -5.36 | 0.02 | 0.00 | 0.00 | Autoinducer 2-binding protein LsrB |
| GE000941 | *eutT* | -5.60 | 0.02 | 0.00 | 0.00 | Ethanolamine utilization cobalamin adenosyltransferase |
| GE000942 | *eutD* | -5.61 | 0.02 | 0.00 | 0.00 | Ethanolamine utilization protein EutD |
| GE000162 | *yhbO* | -5.62 | 0.02 | 0.00 | 0.00 | Protein/nucleic acid deglycase 2 |
| GE000554 | *pduU* | -5.67 | 0.02 | 0.00 | 0.00 | Propanediol utilization protein PduU |
| GE000220 | *lsrC* | -5.69 | 0.02 | 0.00 | 0.00 | Autoinducer 2 import system permease protein LsrC |
| GE000219 | *lsrA* | -5.88 | 0.02 | 0.00 | 0.00 | Autoinducer 2 import ATP-binding protein LsrA |
| GE000553 | *pduV* | -6.04 | 0.02 | 0.00 | 0.00 | Propanediol utilization protein PduV |
| GE004252 | *yjbJ* | -6.13 | 0.01 | 0.00 | 0.00 | UPF0337 protein ECA0631 |
| GE003833 | *osmY* | -6.21 | 0.01 | 0.00 | 0.00 | Osmotically-inducible protein Y |
| GE000756 | *ygaM* | -6.33 | 0.01 | 0.00 | 0.00 | putative protein YgaM |
| GE002586 | *yeaG* | -6.40 | 0.01 | 0.00 | 0.00 | putative protein YeaG |
| GE000562 | *pduL* | -6.44 | 0.01 | 0.00 | 0.00 | Phosphate propanoyltransferase |
| GE000557 | *ADH2* | -6.44 | 0.01 | 0.00 | 0.00 | Aldehyde-alcohol dehydrogenase |
| GE000940 | *eutQ* | -6.52 | 0.01 | 0.00 | 0.00 | Ethanolamine utilization protein EutQ |
| GE000563 | *ccmK* | -6.93 | 0.01 | 0.00 | 0.00 | Carbon dioxide-concentrating mechanism protein CcmK |
| GE000566 | *ddrA* | -6.97 | 0.01 | 0.00 | 0.00 | Diol dehydratase-reactivating factor alpha subunit |
| GE000221 | *lsrD* | -6.97 | 0.01 | 0.00 | 0.00 | Autoinducer 2 import system permease protein LsrD |
| GE004139 | *ecnB* | -7.13 | 0.01 | 0.00 | 0.00 | Entericidin B |
| GE000568 | *pduD* | -7.14 | 0.01 | 0.00 | 0.00 | Propanediol dehydratase medium subunit |
| GE000555 | *--* | -7.19 | 0.01 | 0.00 | 0.00 | hypothetical protein |
| GE000558 | *eutE* | -7.20 | 0.01 | 0.00 | 0.00 | Ethanolamine utilization protein EutE |
| GE000559 | *yvqK* | -7.58 | 0.01 | 0.00 | 0.00 | Corrinoid adenosyltransferase |
| GE000938 | *eutS* | -7.69 | 0.00 | 0.00 | 0.00 | Ethanolamine utilization protein EutS |
| GE000565 | *--* | -7.98 | 0.00 | 0.00 | 0.00 | hypothetical protein |
| GE000939 | *eutP* | -8.09 | 0.00 | 0.00 | 0.00 | Ethanolamine utilization protein EutP |
| GE000570 | *pduB* | -8.14 | 0.00 | 0.00 | 0.00 | Propanediol utilization protein PduB |
| GE000569 | *pduC* | -8.28 | 0.00 | 0.00 | 0.00 | Propanediol dehydratase large subunit |
| GE000567 | *pduE* | -8.76 | 0.00 | 0.00 | 0.00 | Propanediol dehydratase small subunit |
| GE000571 | *pduA* | -9.26 | 0.00 | 0.00 | 0.00 | Propanediol utilization protein PduA |
| GE003032 | *--* | -13.89 | 0.00 | 0.00 | 0.00 | hypothetical protein |
| GE004192 | *phnN* | -14.17 | 0.00 | 0.00 | 0.00 | Ribose 1,5-bisphosphate phosphokinase PhnN |
| GE000560 | *eutN* | -14.64 | 0.00 | 0.00 | 0.00 | Ethanolamine utilization protein EutN |
| GE002333 | *yqaE* | -15.07 | 0.00 | 0.00 | 0.00 | UPF0057 membrane protein PA0567 |
| GE000561 | *--* | -15.72 | 0.00 | 0.00 | 0.00 | hypothetical protein |
| GE000564 | *pduA* | -16.25 | 0.00 | 0.00 | 0.00 | Propanediol utilization protein PduA |
| GE000659 | *rpoS* | -22.01 | 0.00 | 0.00 | 0.00 | RNA polymerase sigma factor RpoS |

**Table S5 The DEGs of HKE9 HKE9-C-M-*rpoS* with FDR < 0.05 and | log2FC | > 1.**

| id | Symbol | log2(FC) | 2-^ΔΔT^ | Pvalue | FDR | Description |
| --- | --- | --- | --- | --- | --- | --- |
| GE003776 | *oadG* | 13.71 | 13406.67 | 0.00 | 0.00 | Oxaloacetate decarboxylase gamma chain |
| GE003943 | *--* | 11.81 | 3583.33 | 0.00 | 0.01 | hypothetical protein |
| GE000617 | *metI* | 11.58 | 3053.33 | 0.00 | 0.01 | putative D-methionine transport system permease protein MetI |
| GE005138 | *virB11* | 9.56 | 755.00 | 0.00 | 0.00 | Type IV secretion system protein VirB11 |
| GE000180 | *gatY* | 6.54 | 93.06 | 0.00 | 0.00 | D-tagatose-1,6-bisphosphate aldolase subunit GatY |
| GE003310 | *cadA* | 6.18 | 72.67 | 0.00 | 0.00 | Inducible lysine decarboxylase |
| GE000237 | *--* | 6.07 | 67.38 | 0.00 | 0.00 | putative 15.0 kDa protein in dhaT-dhaS intergenic region |
| GE003746 | *araB* | 5.76 | 54.25 | 0.00 | 0.00 | Ribulokinase |
| GE000179 | *lacC* | 5.24 | 37.77 | 0.00 | 0.00 | Tagatose-6-phosphate kinase |
| GE003311 | *cadB* | 5.03 | 32.58 | 0.00 | 0.00 | putative cadaverine/lysine antiporter |
| GE003309 | *dtpC* | 4.84 | 28.60 | 0.00 | 0.00 | Dipeptide and tripeptide permease C |
| GE001483 | *tdcB* | 4.67 | 25.39 | 0.00 | 0.00 | L-threonine dehydratase catabolic TdcB |
| GE001484 | *tdcC* | 4.36 | 20.48 | 0.00 | 0.00 | Threonine/serine transporter TdcC |
| GE001486 | *tdcE* | 4.33 | 20.12 | 0.00 | 0.00 | PFL-like enzyme TdcE |
| GE003747 | *araA* | 4.26 | 19.10 | 0.00 | 0.00 | L-arabinose isomerase |
| GE004022 | *--* | 4.14 | 17.64 | 0.00 | 0.00 | hypothetical protein |
| GE001485 | *tdcD* | 3.93 | 15.22 | 0.00 | 0.00 | Propionate kinase |
| GE003778 | *oadB* | 3.80 | 13.91 | 0.00 | 0.00 | Oxaloacetate decarboxylase beta chain |
| GE000492 | *pecM* | 3.77 | 13.68 | 0.01 | 0.04 | Protein PecM |
| GE004016 | *iolG* | 3.69 | 12.88 | 0.00 | 0.00 | Inositol 2-dehydrogenase |
| GE004697 | *lldD* | 3.66 | 12.60 | 0.00 | 0.00 | L-lactate dehydrogenase |
| GE001482 | *tdcA* | 3.55 | 11.72 | 0.00 | 0.00 | HTH-type transcriptional regulator TdcA |
| GE000178 | *fruA* | 3.30 | 9.87 | 0.00 | 0.00 | PTS system fructose-specific EIIABC component |
| GE001284 | *--* | 3.29 | 9.77 | 0.01 | 0.03 | hypothetical protein |
| GE004017 | *iolD* | 3.15 | 8.88 | 0.00 | 0.00 | 3D-(3,5/4)-trihydroxycyclohexane-1,2-dione hydrolase |
| GE003748 | *araD* | 3.10 | 8.58 | 0.00 | 0.00 | L-ribulose-5-phosphate 4-epimerase AraD |
| GE001923 | *fbpB1* | 3.07 | 8.38 | 0.00 | 0.00 | Putative ferric transport system permease protein FbpB 1 |
| GE004699 | *lldP* | 3.06 | 8.37 | 0.00 | 0.00 | L-lactate permease |
| GE005013 | *--* | 3.06 | 8.36 | 0.00 | 0.00 | hypothetical protein |
| GE002041 | *asd* | 2.87 | 7.34 | 0.00 | 0.00 | Aspartate-semialdehyde dehydrogenase |
| GE003918 | *thpA* | 2.83 | 7.13 | 0.00 | 0.00 | D-threitol-binding protein |
| GE004015 | *iolG* | 2.82 | 7.08 | 0.00 | 0.00 | Inositol 2-dehydrogenase |
| GE004698 | *lldR* | 2.80 | 6.95 | 0.00 | 0.00 | Putative L-lactate dehydrogenase operon regulatory protein |
| GE003517 | *ecpB* | 2.74 | 6.66 | 0.00 | 0.02 | putative fimbrial chaperone EcpB |
| GE003252 | *MT0929* | 2.72 | 6.59 | 0.00 | 0.00 | putative protein MT0929 |
| GE001645 | *molC* | 2.72 | 6.58 | 0.00 | 0.01 | putative ABC transporter ATP-binding protein HI_1470 |
| GE004021 | *bauC* | 2.69 | 6.45 | 0.00 | 0.00 | Putative 3-oxopropanoate dehydrogenase |
| GE004018 | *iolC* | 2.63 | 6.20 | 0.00 | 0.00 | 5-dehydro-2-deoxygluconokinase |
| GE003893 | *btsT* | 2.62 | 6.14 | 0.00 | 0.00 | Pyruvate/proton symporter BtsT |
| GE004020 | *iolB* | 2.61 | 6.12 | 0.00 | 0.00 | 5-deoxy-glucuronate isomerase |
| GE004014 | *iolH* | 2.57 | 5.95 | 0.00 | 0.00 | Protein IolH |
| GE003173 | *suhB* | 2.56 | 5.91 | 0.00 | 0.00 | Inositol-1-monophosphatase |
| GE004603 | *acrB* | 2.56 | 5.88 | 0.01 | 0.03 | Multidrug efflux pump subunit AcrB |
| GE000177 | *fruB* | 2.53 | 5.80 | 0.00 | 0.00 | Multiphosphoryl transfer protein |
| GE000241 | *ddrA* | 2.51 | 5.68 | 0.00 | 0.00 | Diol dehydratase-reactivating factor alpha subunit |
| GE004013 | *iolE* | 2.44 | 5.43 | 0.00 | 0.00 | Inosose dehydratase |
| GE001849 | *frmR* | 2.37 | 5.17 | 0.00 | 0.00 | putative protein in bioA 5'region |
| GE003379 | *lacK* | 2.34 | 5.07 | 0.00 | 0.00 | Lactose transport ATP-binding protein LacK |
| GE001939 | *xylL* | 2.33 | 5.02 | 0.00 | 0.00 | 1,6-dihydroxycyclohexa-2,4-diene-1-carboxylate dehydrogenase |
| GE001711 | *ccmB* | 2.31 | 4.97 | 0.00 | 0.00 | Heme exporter protein B |
| GE004604 | *mexB* | 2.23 | 4.70 | 0.00 | 0.00 | Multidrug resistance protein MexB |
| GE000176 | *gatZ* | 2.22 | 4.66 | 0.00 | 0.00 | D-tagatose-1,6-bisphosphate aldolase subunit GatZ |
| GE003276 | *--* | 2.22 | 4.65 | 0.01 | 0.02 | hypothetical protein |
| GE000365 | *argO* | 2.22 | 4.64 | 0.01 | 0.02 | Arginine exporter protein ArgO |
| GE000035 | *arfA* | 2.21 | 4.64 | 0.00 | 0.00 | Alternative ribosome-rescue factor A |
| GE001378 | *araF* | 2.20 | 4.58 | 0.00 | 0.00 | L-arabinose-binding periplasmic protein |
| GE004386 | *AO090005000447* | 2.19 | 4.56 | 0.01 | 0.04 | hypothetical protein |
| GE000234 | *yvqK* | 2.18 | 4.53 | 0.01 | 0.02 | Corrinoid adenosyltransferase |
| GE003248 | *ctpF* | 2.18 | 4.52 | 0.00 | 0.00 | putative cation-transporting ATPase F |
| GE001661 | *msuD* | 2.17 | 4.50 | 0.00 | 0.01 | Methanesulfonate monooxygenase |
| GE000470 | *arsB* | 2.16 | 4.48 | 0.01 | 0.04 | Arsenical pump membrane protein |
| GE002008 | *rbcR* | 2.16 | 4.46 | 0.00 | 0.00 | hypothetical protein |
| GE002027 | *hyuE* | 2.15 | 4.43 | 0.00 | 0.00 | Hydantoin racemase |
| GE004799 | *nikD* | 2.15 | 4.42 | 0.00 | 0.00 | Nickel import ATP-binding protein NikD |
| GE003253 | *--* | 2.14 | 4.40 | 0.00 | 0.00 | hypothetical protein |
| GE001184 | *tnpR* | 2.13 | 4.37 | 0.00 | 0.00 | Transposon Tn2501 resolvase |
| GE002202 | *sotB* | 2.09 | 4.26 | 0.00 | 0.00 | putative sugar efflux transporter |
| GE003378 | *cycB* | 2.08 | 4.23 | 0.00 | 0.00 | Cyclodextrin-binding protein |
| GE004734 | *ettA* | 2.07 | 4.21 | 0.00 | 0.00 | Energy-dependent translational throttle protein EttA |
| GE002091 | *pgtP* | 2.03 | 4.08 | 0.00 | 0.00 | Phosphoglycerate transporter protein |
| GE001420 | *ybjX* | 2.03 | 4.07 | 0.00 | 0.00 | putative protein YbjX |
| GE003777 | *oadA* | 2.00 | 4.01 | 0.00 | 0.00 | Oxaloacetate decarboxylase alpha chain |
| GE005091 | *--* | -2.00 | 0.25 | 0.00 | 0.00 | hypothetical protein |
| GE002383 | *ompS2* | -2.00 | 0.25 | 0.00 | 0.01 | Outer membrane protein S2 |
| GE001177 | *BDS1* | -2.01 | 0.25 | 0.00 | 0.00 | hypothetical protein |
| GE004300 | *thiC* | -2.01 | 0.25 | 0.00 | 0.00 | Phosphomethylpyrimidine synthase |
| GE002444 | *bcsA* | -2.02 | 0.25 | 0.00 | 0.00 | Cellulose synthase catalytic subunit |
| GE001381 | *otsB* | -2.02 | 0.25 | 0.00 | 0.00 | Trehalose-phosphate phosphatase |
| GE004302 | *thiF* | -2.02 | 0.25 | 0.00 | 0.00 | Sulfur carrier protein ThiS adenylyltransferase |
| GE001286 | *dosC* | -2.03 | 0.25 | 0.00 | 0.00 | Diguanylate cyclase DosC |
| GE004301 | *thiE* | -2.03 | 0.25 | 0.00 | 0.00 | Thiamine-phosphate synthase |
| GE001972 | *--* | -2.04 | 0.24 | 0.00 | 0.01 | hypothetical protein |
| GE004172 | *cbpA* | -2.04 | 0.24 | 0.00 | 0.00 | Curved DNA-binding protein |
| GE000199 | *yqjE* | -2.05 | 0.24 | 0.00 | 0.00 | hypothetical protein |
| GE002554 | *astD* | -2.07 | 0.24 | 0.00 | 0.00 | N-succinylglutamate 5-semialdehyde dehydrogenase |
| GE001421 | *pphA* | -2.07 | 0.24 | 0.00 | 0.00 | Serine/threonine-protein phosphatase 1 |
| GE001160 | *yehX* | -2.07 | 0.24 | 0.00 | 0.00 | Glycine betaine uptake system ATP-binding protein YehX |
| GE005079 | *traA* | -2.09 | 0.23 | 0.01 | 0.03 | Pilin |
| GE002408 | *--* | -2.10 | 0.23 | 0.01 | 0.04 | hypothetical protein |
| GE002253 | *ynfD* | -2.13 | 0.23 | 0.00 | 0.00 | hypothetical protein |
| GE002555 | *astA* | -2.13 | 0.23 | 0.00 | 0.00 | Arginine N-succinyltransferase |
| GE001358 | *amyA* | -2.13 | 0.23 | 0.00 | 0.00 | Cytoplasmic alpha-amylase |
| GE001634 | *sufS* | -2.13 | 0.23 | 0.00 | 0.00 | Cysteine desulfurase |
| GE000185 | *garP* | -2.14 | 0.23 | 0.00 | 0.00 | putative galactarate transporter |
| GE002552 | *astB* | -2.14 | 0.23 | 0.00 | 0.00 | N-succinylarginine dihydrolase |
| GE001474 | *treA* | -2.17 | 0.22 | 0.00 | 0.00 | Periplasmic trehalase |
| GE001382 | *otsA* | -2.18 | 0.22 | 0.00 | 0.00 | Trehalose-6-phosphate synthase |
| GE002441 | *pleD* | -2.19 | 0.22 | 0.00 | 0.00 | Response regulator PleD |
| GE004208 | *yjcH* | -2.21 | 0.22 | 0.00 | 0.00 | hypothetical protein |
| GE004305 | *thiH* | -2.22 | 0.21 | 0.00 | 0.00 | 2-iminoacetate synthase |
| GE002737 | *PA2418* | -2.25 | 0.21 | 0.00 | 0.00 | Putative quercetin 2,3-dioxygenase PA2418 |
| GE000860 | *yphA* | -2.25 | 0.21 | 0.00 | 0.00 | hypothetical protein |
| GE004173 | *cbpM* | -2.26 | 0.21 | 0.00 | 0.00 | Chaperone modulatory protein CbpM |
| GE003901 | *yjiN* | -2.28 | 0.21 | 0.00 | 0.00 | hypothetical protein |
| GE002128 | *HI_0093* | -2.30 | 0.20 | 0.00 | 0.00 | putative protein HI_0093 |
| GE002120 | *carA* | -2.30 | 0.20 | 0.00 | 0.00 | Caffeate CoA-transferase |
| GE002440 | *--* | -2.33 | 0.20 | 0.00 | 0.00 | hypothetical protein |
| GE000200 | *yqjD* | -2.33 | 0.20 | 0.00 | 0.00 | Protein ElaB |
| GE004096 | *MT2327* | -2.34 | 0.20 | 0.00 | 0.00 | putative protein MT2327 |
| GE004840 | *ugpE* | -2.35 | 0.20 | 0.00 | 0.01 | sn-glycerol-3-phosphate transport system permease protein UgpE |
| GE001159 | *yehY* | -2.36 | 0.20 | 0.00 | 0.00 | Glycine betaine uptake system permease protein YehY |
| GE000195 | *yhaH* | -2.36 | 0.19 | 0.00 | 0.00 | Inner membrane protein YhaH |
| GE002998 | *ybhN* | -2.36 | 0.19 | 0.00 | 0.00 | Inner membrane protein YbhN |
| GE004576 | *--* | -2.37 | 0.19 | 0.00 | 0.00 | hypothetical protein |
| GE003136 | *KPK_3910* | -2.38 | 0.19 | 0.00 | 0.00 | UPF0250 protein KPK_3910 |
| GE004756 | *dppF* | -2.41 | 0.19 | 0.00 | 0.00 | Dipeptide transport ATP-binding protein DppF |
| GE000201 | *yqjC* | -2.42 | 0.19 | 0.00 | 0.00 | hypothetical protein |
| GE002127 | *glxK* | -2.42 | 0.19 | 0.00 | 0.00 | Glycerate kinase |
| GE002442 | *--* | -2.43 | 0.19 | 0.00 | 0.00 | hypothetical protein |
| GE002445 | *--* | -2.44 | 0.18 | 0.00 | 0.00 | hypothetical protein |
| GE004854 | *glgX* | -2.45 | 0.18 | 0.00 | 0.00 | Glycogen debranching enzyme |
| GE001158 | *yehZ* | -2.45 | 0.18 | 0.00 | 0.00 | Glycine betaine-binding protein YehZ |
| GE004292 | *zraP* | -2.45 | 0.18 | 0.00 | 0.00 | hypothetical protein |
| GE000196 | *yqjG* | -2.46 | 0.18 | 0.00 | 0.00 | Glutathionyl-hydroquinone reductase PcpF |
| GE001083 | *yfaE* | -2.48 | 0.18 | 0.01 | 0.04 | putative ferredoxin-like protein YfaE |
| GE002996 | *ybhP* | -2.50 | 0.18 | 0.00 | 0.00 | putative protein YbhP |
| GE001945 | *pkk2A* | -2.51 | 0.18 | 0.00 | 0.00 | putative polyphosphate kinase PKK2A |
| GE002725 | *msyB* | -2.52 | 0.17 | 0.00 | 0.00 | Acidic protein MsyB |
| GE003183 | *fabG* | -2.53 | 0.17 | 0.00 | 0.00 | 3-oxoacyl-[acyl-carrier-protein] reductase FabG |
| GE004736 | *yiaG* | -2.54 | 0.17 | 0.00 | 0.00 | putative HTH-type transcriptional regulator YiaG |
| GE002369 | *maoA* | -2.55 | 0.17 | 0.00 | 0.00 | Primary amine oxidase |
| GE004701 | *yibT* | -2.55 | 0.17 | 0.00 | 0.00 | putative protein YibT |
| GE003026 | *uspG* | -2.55 | 0.17 | 0.00 | 0.00 | Universal stress protein G |
| GE003244 | *KPN78578_05520* | -2.56 | 0.17 | 0.00 | 0.00 | Putative glutamate--cysteine ligase 2 |
| GE003029 | *nolG* | -2.56 | 0.17 | 0.00 | 0.00 | Nodulation protein NolG |
| GE002551 | *astE* | -2.57 | 0.17 | 0.00 | 0.00 | Succinylglutamate desuccinylase |
| GE002119 | *fadD13* | -2.57 | 0.17 | 0.00 | 0.00 | Long-chain-fatty-acid--CoA ligase FadD13 |
| GE002450 | *NIK1* | -2.58 | 0.17 | 0.00 | 0.00 | Sensor histidine kinase GacS |
| GE002443 | *--* | -2.59 | 0.17 | 0.00 | 0.00 | hypothetical protein |
| GE004755 | *dppD* | -2.59 | 0.17 | 0.00 | 0.00 | Dipeptide transport ATP-binding protein DppD |
| GE002125 | *--* | -2.59 | 0.17 | 0.00 | 0.00 | hypothetical protein |
| GE003676 | *gcd* | -2.60 | 0.16 | 0.00 | 0.00 | Quinoprotein glucose dehydrogenase |
| GE001878 | *ydcT* | -2.62 | 0.16 | 0.00 | 0.00 | putative ABC transporter ATP-binding protein YdcT |
| GE001423 | *yebV* | -2.63 | 0.16 | 0.00 | 0.00 | putative protein YebV |
| GE003028 | *mdtA* | -2.63 | 0.16 | 0.00 | 0.00 | Multidrug resistance protein MdtA |
| GE002447 | *--* | -2.66 | 0.16 | 0.00 | 0.00 | hypothetical protein |
| GE002126 | *--* | -2.66 | 0.16 | 0.00 | 0.00 | hypothetical protein |
| GE001629 | *--* | -2.66 | 0.16 | 0.00 | 0.00 | hypothetical protein |
| GE003389 | *ybaY* | -2.67 | 0.16 | 0.00 | 0.00 | putative lipoprotein YbaY |
| GE002627 | *yciF* | -2.67 | 0.16 | 0.00 | 0.00 | Protein YciF |
| GE001816 | *sodC* | -2.68 | 0.16 | 0.00 | 0.00 | Superoxide dismutase |
| GE003025 | *ctpF* | -2.68 | 0.16 | 0.00 | 0.00 | putative cation-transporting ATPase F |
| GE002976 | *ybiO* | -2.68 | 0.16 | 0.00 | 0.00 | Moderate conductance mechanosensitive channel YbiO |
| GE002058 | *ydhS* | -2.72 | 0.15 | 0.00 | 0.00 | putative protein YdhS |
| GE003692 | *aroP* | -2.73 | 0.15 | 0.00 | 0.00 | Aromatic amino acid transport protein AroP |
| GE004095 | *yjfY* | -2.74 | 0.15 | 0.00 | 0.00 | hypothetical protein |
| GE003559 | *gabP* | -2.75 | 0.15 | 0.00 | 0.00 | GABA permease |
| GE002805 | *pdeG* | -2.76 | 0.15 | 0.00 | 0.00 | putative cyclic di-GMP phosphodiesterase PdeG |
| GE003766 | *ygdI* | -2.77 | 0.15 | 0.00 | 0.00 | putative lipoprotein YgdI |
| GE001861 | *ydcK* | -2.78 | 0.15 | 0.00 | 0.00 | putative acetyltransferase YdcK |
| GE004137 | *blc* | -2.78 | 0.15 | 0.00 | 0.00 | Outer membrane lipoprotein Blc |
| GE004748 | *yhjY* | -2.79 | 0.14 | 0.00 | 0.00 | putative protein YhjY |
| GE001881 | *patD* | -2.79 | 0.14 | 0.00 | 0.00 | Gamma-aminobutyraldehyde dehydrogenase |
| GE002449 | *dhkJ* | -2.81 | 0.14 | 0.00 | 0.00 | Autoinducer 2 sensor kinase/phosphatase LuxQ |
| GE002367 | *paaA* | -2.82 | 0.14 | 0.00 | 0.00 | 1,2-phenylacetyl-CoA epoxidase, subunit A |
| GE002451 | *mndB* | -2.82 | 0.14 | 0.00 | 0.00 | hypothetical protein |
| GE002626 | *yciE* | -2.83 | 0.14 | 0.00 | 0.00 | Protein YciE |
| GE001877 | *ydcS* | -2.83 | 0.14 | 0.00 | 0.00 | Bifunctional polyhydroxybutyrate synthase / ABC transporter periplasmic binding protein |
| GE004752 | *dppA* | -2.89 | 0.14 | 0.00 | 0.00 | Periplasmic dipeptide transport protein |
| GE002121 | *fabG* | -2.89 | 0.13 | 0.00 | 0.00 | 3-oxoacyl-[acyl-carrier-protein] reductase FabG |
| GE002625 | *cotJC* | -2.89 | 0.13 | 0.00 | 0.00 | Protein CotJC |
| GE002003 | *DPEP1* | -2.91 | 0.13 | 0.00 | 0.00 | hypothetical protein |
| GE001035 | *yfcG* | -2.91 | 0.13 | 0.00 | 0.00 | Disulfide-bond oxidoreductase YfcG |
| GE001977 | *--* | -2.92 | 0.13 | 0.00 | 0.00 | hypothetical protein |
| GE002490 | *osmB* | -2.92 | 0.13 | 0.00 | 0.00 | Osmotically-inducible lipoprotein B |
| GE004754 | *dppC* | -2.95 | 0.13 | 0.00 | 0.00 | Dipeptide transport system permease protein DppC |
| GE004844 | *ggt* | -2.99 | 0.13 | 0.00 | 0.00 | Glutathione hydrolase proenzyme |
| GE003897 | *ssdA* | -2.99 | 0.13 | 0.00 | 0.00 | Succinate-semialdehyde dehydrogenase |
| GE000935 | *tktB* | -3.01 | 0.12 | 0.00 | 0.00 | Transketolase 2 |
| GE000659 | *rpoS* | -3.01 | 0.12 | 0.00 | 0.00 | RNA polymerase sigma factor RpoS |
| GE002547 | *osmE* | -3.01 | 0.12 | 0.00 | 0.00 | hypothetical protein |
| GE004777 | *yhjG* | -3.01 | 0.12 | 0.00 | 0.00 | AsmA family protein YhjG |
| GE003044 | *acrZ* | -3.02 | 0.12 | 0.01 | 0.02 | Multidrug efflux pump accessory protein AcrZ |
| GE004079 | *ytfK* | -3.02 | 0.12 | 0.00 | 0.00 | hypothetical protein |
| GE001925 | *narV* | -3.04 | 0.12 | 0.00 | 0.00 | Respiratory nitrate reductase 2 gamma chain |
| GE002124 | *RGN* | -3.05 | 0.12 | 0.00 | 0.00 | L-arabinolactonase |
| GE001612 | *yeaQ* | -3.05 | 0.12 | 0.00 | 0.00 | UPF0410 protein YeaQ |
| GE002497 | *acnA* | -3.07 | 0.12 | 0.00 | 0.00 | Aconitate hydratase A |
| GE004905 | *fic* | -3.07 | 0.12 | 0.00 | 0.00 | putative protein adenylyltransferase Fic |
| GE002240 | *yedK* | -3.08 | 0.12 | 0.00 | 0.00 | SOS response-associated protein YedK |
| GE002122 | *thlA* | -3.11 | 0.12 | 0.00 | 0.00 | Acetyl-CoA acetyltransferase |
| GE000217 | *lsrK* | -3.12 | 0.12 | 0.00 | 0.00 | Autoinducer-2 kinase |
| GE005103 | *hxcR* | -3.13 | 0.11 | 0.00 | 0.00 | putative type II secretion system protein HxcR |
| GE000218 | *lsrR* | -3.13 | 0.11 | 0.00 | 0.00 | Transcriptional regulator LsrR |
| GE004853 | *glgB* | -3.14 | 0.11 | 0.00 | 0.00 | 1,4-alpha-glucan branching enzyme GlgB |
| GE000225 | *patA* | -3.15 | 0.11 | 0.00 | 0.00 | Putrescine aminotransferase |
| GE002587 | *KPN78578_11640* | -3.18 | 0.11 | 0.00 | 0.00 | UPF0229 protein KPN78578_11640 |
| GE002123 | *ttuB* | -3.19 | 0.11 | 0.00 | 0.00 | Putative tartrate transporter |
| GE002448 | *--* | -3.20 | 0.11 | 0.00 | 0.00 | hypothetical protein |
| GE000661 | *yhcA* | -3.20 | 0.11 | 0.00 | 0.00 | putative MFS-type transporter YhcA |
| GE002289 | *--* | -3.23 | 0.11 | 0.00 | 0.00 | hypothetical protein |
| GE000988 | *ipdC* | -3.24 | 0.11 | 0.00 | 0.00 | Indole-3-pyruvate decarboxylase |
| GE002236 | *ibp* | -3.26 | 0.10 | 0.00 | 0.00 | Small heat shock protein ibp |
| GE002628 | *yciG* | -3.27 | 0.10 | 0.00 | 0.00 | putative protein YciG |
| GE001880 | *ydcV* | -3.27 | 0.10 | 0.00 | 0.00 | Inner membrane ABC transporter permease protein YdcV |
| GE001879 | *ydcU* | -3.32 | 0.10 | 0.00 | 0.00 | Inner membrane ABC transporter permease protein YdcU |
| GE000005 | *bfr* | -3.32 | 0.10 | 0.00 | 0.00 | Bacterioferritin |
| GE002806 | *--* | -3.32 | 0.10 | 0.00 | 0.00 | hypothetical protein |
| GE003033 | *--* | -3.35 | 0.10 | 0.00 | 0.00 | hypothetical protein |
| GE004753 | *dppB* | -3.36 | 0.10 | 0.00 | 0.00 | Dipeptide transport system permease protein DppB |
| GE002807 | *glgP* | -3.38 | 0.10 | 0.00 | 0.00 | Glycogen phosphorylase |
| GE001153 | *yohC* | -3.39 | 0.10 | 0.00 | 0.00 | hypothetical protein |
| GE004619 | *yahK* | -3.40 | 0.10 | 0.00 | 0.00 | Aldehyde reductase YahK |
| GE001929 | *narU* | -3.41 | 0.09 | 0.00 | 0.00 | Nitrate/nitrite transporter NarU |
| GE001856 | *ydcJ* | -3.43 | 0.09 | 0.00 | 0.00 | putative protein YdcJ |
| GE002980 | *ybiI* | -3.45 | 0.09 | 0.00 | 0.00 | putative protein YbiI |
| GE000781 | *yvaG* | -3.46 | 0.09 | 0.00 | 0.00 | Nodulation protein G |
| GE004193 | *phnP* | -3.46 | 0.09 | 0.00 | 0.00 | Phosphoribosyl 1,2-cyclic phosphate phosphodiesterase |
| GE002446 | *--* | -3.54 | 0.09 | 0.00 | 0.00 | hypothetical protein |
| GE003555 | *glaH* | -3.55 | 0.09 | 0.00 | 0.00 | Glutarate 2-hydroxylase |
| GE003038 | *ariR* | -3.56 | 0.08 | 0.00 | 0.00 | Regulatory protein AriR |
| GE000936 | *talA* | -3.57 | 0.08 | 0.00 | 0.00 | Transaldolase A |
| GE003037 | *--* | -3.63 | 0.08 | 0.00 | 0.00 | hypothetical protein |
| GE001927 | *narY* | -3.63 | 0.08 | 0.00 | 0.00 | Respiratory nitrate reductase 2 beta chain |
| GE003036 | *ycgZ* | -3.65 | 0.08 | 0.00 | 0.00 | putative two-component-system connector protein YcgZ |
| GE002972 | *dps* | -3.67 | 0.08 | 0.00 | 0.00 | DNA protection during starvation protein |
| GE004812 | *yhhT* | -3.70 | 0.08 | 0.00 | 0.00 | Putative transport protein YhhT |
| GE000220 | *lsrC* | -3.75 | 0.07 | 0.00 | 0.00 | Autoinducer 2 import system permease protein LsrC |
| GE003053 | *ybgS* | -3.80 | 0.07 | 0.00 | 0.00 | putative protein YbgS |
| GE003480 | *psiF* | -3.81 | 0.07 | 0.00 | 0.00 | Phosphate starvation-inducible protein PsiF |
| GE000219 | *lsrA* | -3.88 | 0.07 | 0.00 | 0.00 | Autoinducer 2 import ATP-binding protein LsrA |
| GE000223 | *lsrF* | -3.91 | 0.07 | 0.00 | 0.00 | 3-hydroxy-5-phosphonooxypentane-2,4-dione thiolase |
| GE004804 | *padC* | -3.92 | 0.07 | 0.00 | 0.00 | putative phenolic acid decarboxylase |
| GE002090 | *kbp* | -3.93 | 0.07 | 0.00 | 0.00 | Potassium binding protein Kbp |
| GE003558 | *gabT* | -3.95 | 0.06 | 0.00 | 0.00 | 4-aminobutyrate aminotransferase GabT |
| GE003557 | *gabD* | -3.95 | 0.06 | 0.00 | 0.00 | Succinate-semialdehyde dehydrogenase GabD |
| GE000074 | *yhcO* | -3.96 | 0.06 | 0.00 | 0.00 | putative protein YhcO |
| GE000224 | *lsrG* | -3.98 | 0.06 | 0.00 | 0.00 | (4S)-4-hydroxy-5-phosphonooxypentane-2,3-dione isomerase |
| GE000600 | *ygdI* | -4.02 | 0.06 | 0.00 | 0.00 | putative lipoprotein YgdI |
| GE000221 | *lsrD* | -4.04 | 0.06 | 0.00 | 0.00 | Autoinducer 2 import system permease protein LsrD |
| GE002319 | *fumC* | -4.09 | 0.06 | 0.00 | 0.00 | Fumarate hydratase class II |
| GE003475 | *yaiA* | -4.19 | 0.05 | 0.00 | 0.00 | putative protein YaiA |
| GE001467 | *ycgB* | -4.23 | 0.05 | 0.00 | 0.00 | putative protein YcgB |
| GE003313 | *--* | -4.24 | 0.05 | 0.00 | 0.00 | hypothetical protein |
| GE003556 | *lhgD* | -4.26 | 0.05 | 0.00 | 0.00 | L-2-hydroxyglutarate dehydrogenase |
| GE000866 | *csiE* | -4.27 | 0.05 | 0.00 | 0.00 | Stationary phase-inducible protein CsiE |
| GE000222 | *lsrB* | -4.28 | 0.05 | 0.00 | 0.00 | Autoinducer 2-binding protein LsrB |
| GE004192 | *phnN* | -4.32 | 0.05 | 0.00 | 0.00 | Ribose 1,5-bisphosphate phosphokinase PhnN |
| GE004904 | *yhfG* | -4.32 | 0.05 | 0.00 | 0.00 | putative protein YhfG |
| GE001191 | *fbaB* | -4.38 | 0.05 | 0.00 | 0.00 | Fructose-bisphosphate aldolase class 1 |
| GE004870 | *--* | -4.45 | 0.05 | 0.00 | 0.00 | hypothetical protein |
| GE003032 | *--* | -4.50 | 0.04 | 0.00 | 0.00 | hypothetical protein |
| GE001928 | *narZ* | -4.50 | 0.04 | 0.00 | 0.00 | Respiratory nitrate reductase 2 alpha chain |
| GE000557 | *ADH2* | -4.63 | 0.04 | 0.00 | 0.00 | Aldehyde-alcohol dehydrogenase |
| GE001973 | *ydeI* | -4.64 | 0.04 | 0.00 | 0.00 | putative protein YdeI |
| GE002540 | *katE* | -4.84 | 0.03 | 0.00 | 0.00 | Catalase HPII |
| GE003832 | *KPK_4780* | -4.84 | 0.03 | 0.00 | 0.00 | UPF0391 membrane protein KPK_4780 |
| GE000572 | *pduF* | -4.92 | 0.03 | 0.00 | 0.00 | Propanediol diffusion facilitator |
| GE003833 | *osmY* | -4.92 | 0.03 | 0.00 | 0.00 | Osmotically-inducible protein Y |
| GE000564 | *pduA* | -4.94 | 0.03 | 0.00 | 0.00 | Propanediol utilization protein PduA |
| GE001975 | *osmC* | -4.94 | 0.03 | 0.00 | 0.00 | Peroxiredoxin OsmC |
| GE001964 | *yhxD* | -4.94 | 0.03 | 0.00 | 0.00 | putative oxidoreductase YhxD |
| GE001926 | *narW* | -5.10 | 0.03 | 0.00 | 0.00 | putative nitrate reductase molybdenum cofactor assembly chaperone NarW |
| GE000552 | *pduW* | -5.11 | 0.03 | 0.00 | 0.00 | putative propionate kinase |
| GE002586 | *yeaG* | -5.15 | 0.03 | 0.00 | 0.00 | putative protein YeaG |
| GE000756 | *ygaM* | -5.21 | 0.03 | 0.00 | 0.00 | putative protein YgaM |
| GE000559 | *yvqK* | -5.40 | 0.02 | 0.00 | 0.00 | Corrinoid adenosyltransferase |
| GE000558 | *eutE* | -5.49 | 0.02 | 0.00 | 0.00 | Ethanolamine utilization protein EutE |
| GE000562 | *pduL* | -5.60 | 0.02 | 0.00 | 0.00 | Phosphate propanoyltransferase |
| GE000553 | *pduV* | -5.61 | 0.02 | 0.00 | 0.00 | Propanediol utilization protein PduV |
| GE000556 | *rnfC* | -5.73 | 0.02 | 0.00 | 0.00 | Na(+)-translocating ferredoxin:NAD(+) oxidoreductase complex subunit C |
| GE000162 | *yhbO* | -5.74 | 0.02 | 0.00 | 0.00 | Protein/nucleic acid deglycase 2 |
| GE000555 | *--* | -5.80 | 0.02 | 0.00 | 0.00 | hypothetical protein |
| GE000554 | *pduU* | -5.89 | 0.02 | 0.00 | 0.00 | Propanediol utilization protein PduU |
| GE000570 | *pduB* | -5.91 | 0.02 | 0.00 | 0.00 | Propanediol utilization protein PduB |
| GE000563 | *ccmK* | -5.96 | 0.02 | 0.00 | 0.00 | Carbon dioxide-concentrating mechanism protein CcmK |
| GE004139 | *ecnB* | -5.98 | 0.02 | 0.00 | 0.00 | Entericidin B |
| GE000561 | *--* | -6.29 | 0.01 | 0.00 | 0.00 | hypothetical protein |
| GE004252 | *yjbJ* | -6.44 | 0.01 | 0.00 | 0.00 | UPF0337 protein ECA0631 |
| GE000566 | *ddrA* | -6.45 | 0.01 | 0.00 | 0.00 | Diol dehydratase-reactivating factor alpha subunit |
| GE000571 | *pduA* | -6.51 | 0.01 | 0.00 | 0.00 | Propanediol utilization protein PduA |
| GE000568 | *pduD* | -6.92 | 0.01 | 0.00 | 0.00 | Propanediol dehydratase medium subunit |
| GE000569 | *pduC* | -7.19 | 0.01 | 0.00 | 0.00 | Propanediol dehydratase large subunit |
| GE000565 | *--* | -7.22 | 0.01 | 0.00 | 0.00 | hypothetical protein |
| GE000567 | *pduE* | -7.41 | 0.01 | 0.00 | 0.00 | Propanediol dehydratase small subunit |
| GE004005 | *can* | -11.44 | 0.00 | 0.01 | 0.04 | Carbonic anhydrase 2 |
| GE000560 | *eutN* | -14.64 | 0.00 | 0.00 | 0.00 | Ethanolamine utilization protein EutN |
| GE002333 | *yqaE* | -15.07 | 0.00 | 0.00 | 0.00 | UPF0057 membrane protein PA0567 |
| GE001354 | *yodD* | -15.94 | 0.00 | 0.00 | 0.00 | hypothetical protein |

**Table S6 The DEGs of HKE9-M-*rpoS* vs HKE9-C-M-*rpoS* with FDR < 0.05 and | log2FC | > 1.**

| id | Symbol | log2(FC) | 2^^-ΔΔT^ | Pvalue | FDR | Description |
| --- | --- | --- | --- | --- | --- | --- |
| GE000659 | *rpoS* | 19.00 | 523533.33 | 0.00 | 0.00 | RNA polymerase sigma factor RpoS |
| GE003943 | *--* | 11.81 | 3583.33 | 0.00 | 0.01 | hypothetical protein |
| GE000617 | *metI* | 11.58 | 3053.33 | 0.00 | 0.01 | putative D-methionine transport system permease protein MetI |
| GE000770 | *yraI* | 10.97 | 2000.00 | 0.01 | 0.04 | putative fimbrial chaperone LpfB |
| GE000939 | *eutP* | 8.59 | 385.30 | 0.00 | 0.00 | Ethanolamine utilization protein EutP |
| GE000938 | *eutS* | 7.98 | 252.13 | 0.00 | 0.00 | Ethanolamine utilization protein EutS |
| GE003310 | *cadA* | 7.60 | 194.57 | 0.00 | 0.00 | Inducible lysine decarboxylase |
| GE000940 | *eutQ* | 7.45 | 174.33 | 0.00 | 0.00 | Ethanolamine utilization protein EutQ |
| GE005138 | *virB11* | 7.39 | 167.78 | 0.00 | 0.00 | Type IV secretion system protein VirB11 |
| GE003311 | *cadB* | 7.06 | 133.60 | 0.00 | 0.00 | putative cadaverine/lysine antiporter |
| GE000942 | *eutD* | 6.64 | 100.06 | 0.00 | 0.00 | Ethanolamine utilization protein EutD |
| GE000941 | *eutT* | 6.60 | 96.81 | 0.00 | 0.00 | Ethanolamine utilization cobalamin adenosyltransferase |
| GE003309 | *dtpC* | 6.22 | 74.48 | 0.00 | 0.00 | Dipeptide and tripeptide permease C |
| GE003746 | *araB* | 5.97 | 62.66 | 0.00 | 0.00 | Ribulokinase |
| GE000947 | *eutG* | 5.54 | 46.55 | 0.00 | 0.00 | Ethanolamine utilization protein EutG |
| GE000946 | *eutJ* | 5.18 | 36.27 | 0.00 | 0.00 | Ethanolamine utilization protein EutJ |
| GE000944 | *eutN* | 5.09 | 33.97 | 0.00 | 0.00 | Ethanolamine utilization protein EutN |
| GE000945 | *eutE* | 5.05 | 33.17 | 0.00 | 0.00 | Ethanolamine utilization protein EutE |
| GE000948 | *eutH* | 5.03 | 32.56 | 0.00 | 0.00 | Ethanolamine utilization protein EutH |
| GE000180 | *gatY* | 4.75 | 26.97 | 0.00 | 0.00 | D-tagatose-1,6-bisphosphate aldolase subunit GatY |
| GE000949 | *eutA* | 4.49 | 22.40 | 0.00 | 0.00 | Ethanolamine utilization protein EutA |
| GE004022 | *--* | 4.46 | 22.04 | 0.00 | 0.00 | hypothetical protein |
| GE003747 | *araA* | 4.42 | 21.39 | 0.00 | 0.00 | L-arabinose isomerase |
| GE003512 | *glnP* | 4.03 | 16.36 | 0.00 | 0.00 | Putative glutamine transport system permease protein GlnP |
| GE003937 | *MXAN_5909* | 4.00 | 15.99 | 0.00 | 0.00 | putative oxidoreductase MXAN_5909 |
| GE004017 | *iolD* | 3.70 | 13.00 | 0.00 | 0.00 | 3D-(3,5/4)-trihydroxycyclohexane-1,2-dione hydrolase |
| GE000179 | *lacC* | 3.62 | 12.33 | 0.00 | 0.00 | Tagatose-6-phosphate kinase |
| GE002430 | *--* | 3.44 | 10.89 | 0.00 | 0.00 | hypothetical protein |
| GE001923 | *fbpB1* | 3.42 | 10.72 | 0.00 | 0.00 | Putative ferric transport system permease protein FbpB 1 |
| GE003940 | *MTS1* | 3.38 | 10.43 | 0.00 | 0.01 | Hydroxymycolate synthase MmaA4 |
| GE000943 | *eutM* | 3.34 | 10.13 | 0.00 | 0.00 | Ethanolamine utilization protein EutM |
| GE004971 | *insB1* | 3.31 | 9.93 | 0.01 | 0.04 | Insertion element IS1 1 protein InsB |
| GE003939 | *--* | 3.22 | 9.32 | 0.00 | 0.00 | hypothetical protein |
| GE004020 | *iolB* | 3.15 | 8.85 | 0.00 | 0.00 | 5-deoxy-glucuronate isomerase |
| GE003938 | *--* | 3.10 | 8.60 | 0.00 | 0.00 | hypothetical protein |
| GE004018 | *iolC* | 3.07 | 8.41 | 0.00 | 0.00 | 5-dehydro-2-deoxygluconokinase |
| GE000178 | *fruA* | 3.01 | 8.04 | 0.00 | 0.00 | PTS system fructose-specific EIIABC component |
| GE003252 | *MT0929* | 3.00 | 8.01 | 0.00 | 0.00 | putative protein MT0929 |
| GE003906 | *ytiA* | 2.96 | 7.78 | 0.00 | 0.02 | hypothetical protein |
| GE000221 | *lsrD* | 2.94 | 7.65 | 0.00 | 0.00 | Autoinducer 2 import system permease protein LsrD |
| GE004016 | *iolG* | 2.88 | 7.35 | 0.00 | 0.00 | Inositol 2-dehydrogenase |
| GE004183 | *yjdN* | 2.86 | 7.28 | 0.00 | 0.00 | hypothetical protein |
| GE004021 | *bauC* | 2.86 | 7.26 | 0.00 | 0.00 | Putative 3-oxopropanoate dehydrogenase |
| GE004603 | *acrB* | 2.84 | 7.17 | 0.01 | 0.04 | Multidrug efflux pump subunit AcrB |
| GE004015 | *iolG* | 2.84 | 7.14 | 0.00 | 0.00 | Inositol 2-dehydrogenase |
| GE003748 | *araD* | 2.80 | 6.94 | 0.00 | 0.01 | L-ribulose-5-phosphate 4-epimerase AraD |
| GE001483 | *tdcB* | 2.79 | 6.93 | 0.00 | 0.00 | L-threonine dehydratase catabolic TdcB |
| GE004710 | *sgbH* | 2.76 | 6.77 | 0.00 | 0.02 | 3-keto-L-gulonate-6-phosphate decarboxylase SgbH |
| GE003936 | *--* | 2.75 | 6.75 | 0.00 | 0.00 | hypothetical protein |
| GE001711 | *ccmB* | 2.60 | 6.07 | 0.00 | 0.00 | Heme exporter protein B |
| GE000177 | *fruB* | 2.59 | 6.04 | 0.00 | 0.00 | Multiphosphoryl transfer protein |
| GE002008 | *rbcR* | 2.59 | 6.03 | 0.00 | 0.00 | hypothetical protein |
| GE004013 | *iolE* | 2.58 | 5.99 | 0.00 | 0.00 | Inosose dehydratase |
| GE004014 | *iolH* | 2.56 | 5.89 | 0.00 | 0.00 | Protein IolH |
| GE001482 | *tdcA* | 2.54 | 5.80 | 0.00 | 0.00 | HTH-type transcriptional regulator TdcA |
| GE004554 | *aglA* | 2.53 | 5.78 | 0.00 | 0.00 | PTS system alpha-glucoside-specific EIICB component |
| GE003918 | *thpA* | 2.49 | 5.63 | 0.00 | 0.00 | D-threitol-binding protein |
| GE003945 | *ybgA* | 2.44 | 5.42 | 0.00 | 0.00 | putative protein YbgA |
| GE000950 | *eutB* | 2.42 | 5.35 | 0.00 | 0.00 | Ethanolamine ammonia-lyase heavy chain |
| GE000249 | *--* | 2.37 | 5.18 | 0.00 | 0.00 | hypothetical protein |
| GE003379 | *lacK* | 2.35 | 5.10 | 0.00 | 0.00 | Lactose transport ATP-binding protein LacK |
| GE000574 | *cbiA* | 2.32 | 4.98 | 0.00 | 0.00 | Cobyrinate a,c-diamide synthase |
| GE001484 | *tdcC* | 2.31 | 4.97 | 0.00 | 0.00 | Threonine/serine transporter TdcC |
| GE003773 | *citD* | 2.31 | 4.95 | 0.00 | 0.00 | Citrate lyase acyl carrier protein |
| GE003778 | *oadB* | 2.31 | 4.94 | 0.00 | 0.00 | Oxaloacetate decarboxylase beta chain |
| GE000576 | *cbiC* | 2.29 | 4.88 | 0.00 | 0.00 | Cobalt-precorrin-8 methylmutase |
| GE001551 | *nasA* | 2.27 | 4.81 | 0.00 | 0.00 | Nitrate reductase |
| GE000237 | *--* | 2.24 | 4.72 | 0.00 | 0.00 | putative 15.0 kDa protein in dhaT-dhaS intergenic region |
| GE000570 | *pduB* | 2.23 | 4.68 | 0.00 | 0.01 | Propanediol utilization protein PduB |
| GE000035 | *arfA* | 2.22 | 4.65 | 0.00 | 0.00 | Alternative ribosome-rescue factor A |
| GE000328 | *intB* | 2.19 | 4.57 | 0.01 | 0.04 | Putative protein IntB |
| GE003944 | *--* | 2.19 | 4.55 | 0.00 | 0.00 | hypothetical protein |
| GE000575 | *cobD* | 2.17 | 4.50 | 0.00 | 0.00 | Cobalamin biosynthesis protein CobD |
| GE001658 | *--* | 2.15 | 4.44 | 0.01 | 0.04 | hypothetical protein |
| GE004396 | *ilvM* | 2.14 | 4.42 | 0.00 | 0.00 | Acetolactate synthase isozyme 2 small subunit |
| GE003185 | *--* | 2.13 | 4.37 | 0.00 | 0.00 | hypothetical protein |
| GE000746 | *ygaZ* | 2.13 | 4.36 | 0.00 | 0.00 | Inner membrane protein YgaZ |
| GE000298 | *yqhD* | 2.09 | 4.26 | 0.00 | 0.00 | Long-chain-alcohol dehydrogenase 2 |
| GE004397 | *ilvG* | 2.08 | 4.24 | 0.00 | 0.00 | Acetolactate synthase isozyme 2 large subunit |
| GE003917 | *rbsA2* | 2.07 | 4.20 | 0.00 | 0.00 | Ribose import ATP-binding protein RbsA 2 |
| GE000578 | *cbiE* | 2.02 | 4.05 | 0.00 | 0.00 | Cobalt-precorrin-7 C(5)-methyltransferase |
| GE000951 | *eutC* | 2.02 | 4.05 | 0.00 | 0.00 | Ethanolamine ammonia-lyase light chain |
| GE000331 | *nupG* | 2.00 | 4.01 | 0.00 | 0.00 | Nucleoside permease NupG |
| GE000219 | *lsrA* | 2.00 | 4.01 | 0.00 | 0.00 | Autoinducer 2 import ATP-binding protein LsrA |
| GE003136 | *KPK_3910* | -2.01 | 0.25 | 0.00 | 0.00 | UPF0250 protein KPK_3910 |
| GE005099 | *--* | -2.03 | 0.24 | 0.00 | 0.00 | hypothetical protein |
| GE005140 | *--* | -2.03 | 0.24 | 0.00 | 0.00 | hypothetical protein |
| GE000522 | *lysA* | -2.07 | 0.24 | 0.00 | 0.00 | Diaminopimelate decarboxylase |
| GE002128 | *HI_0093* | -2.08 | 0.24 | 0.00 | 0.00 | putative protein HI_0093 |
| GE003766 | *ygdI* | -2.08 | 0.24 | 0.00 | 0.00 | putative lipoprotein YgdI |
| GE005100 | *--* | -2.09 | 0.23 | 0.00 | 0.00 | hypothetical protein |
| GE005141 | *--* | -2.09 | 0.23 | 0.00 | 0.00 | hypothetical protein |
| GE003666 | *folK* | -2.12 | 0.23 | 0.00 | 0.00 | 2-amino-4-hydroxy-6- hydroxymethyldihydropteridine pyrophosphokinase |
| GE000195 | *yhaH* | -2.15 | 0.23 | 0.00 | 0.00 | Inner membrane protein YhaH |
| GE001192 | *rbtT* | -2.20 | 0.22 | 0.00 | 0.00 | Ribitol transporter |
| GE005145 | *outE* | -2.20 | 0.22 | 0.00 | 0.00 | Type II secretion system protein E |
| GE000487 | *fim* | -2.22 | 0.21 | 0.00 | 0.00 | Fimbrial subunit type 1 |
| GE002125 | *--* | -2.25 | 0.21 | 0.00 | 0.00 | hypothetical protein |
| GE002127 | *glxK* | -2.31 | 0.20 | 0.00 | 0.00 | Glycerate kinase |
| GE002618 | *--* | -2.34 | 0.20 | 0.00 | 0.00 | hypothetical protein |
| GE002120 | *carA* | -2.36 | 0.19 | 0.00 | 0.00 | Caffeate CoA-transferase |
| GE005090 | *--* | -2.38 | 0.19 | 0.00 | 0.00 | hypothetical protein |
| GE005097 | *traG* | -2.39 | 0.19 | 0.00 | 0.00 | Conjugal transfer protein TraG |
| GE002126 | *--* | -2.42 | 0.19 | 0.00 | 0.00 | hypothetical protein |
| GE005108 | *--* | -2.47 | 0.18 | 0.00 | 0.00 | hypothetical protein |
| GE005149 | *--* | -2.47 | 0.18 | 0.00 | 0.00 | hypothetical protein |
| GE003519 | *ecpR* | -2.47 | 0.18 | 0.00 | 0.01 | HTH-type transcriptional regulator EcpR |
| GE005089 | *--* | -2.52 | 0.17 | 0.00 | 0.00 | hypothetical protein |
| GE002236 | *ibp* | -2.53 | 0.17 | 0.00 | 0.00 | Small heat shock protein ibp |
| GE005082 | *--* | -2.56 | 0.17 | 0.00 | 0.00 | hypothetical protein |
| GE002119 | *fadD13* | -2.60 | 0.16 | 0.00 | 0.00 | Long-chain-fatty-acid--CoA ligase FadD13 |
| GE000085 | *oadA* | -2.66 | 0.16 | 0.01 | 0.05 | Oxaloacetate decarboxylase alpha chain |
| GE001083 | *yfaE* | -2.68 | 0.16 | 0.01 | 0.04 | putative ferredoxin-like protein YfaE |
| GE004114 | *--* | -2.83 | 0.14 | 0.00 | 0.00 | hypothetical protein |
| GE005146 | *--* | -2.88 | 0.14 | 0.00 | 0.00 | hypothetical protein |
| GE002124 | *RGN* | -2.95 | 0.13 | 0.00 | 0.00 | L-arabinolactonase |
| GE002121 | *fabG* | -2.97 | 0.13 | 0.00 | 0.00 | 3-oxoacyl-[acyl-carrier-protein] reductase FabG |
| GE005106 | *--* | -3.08 | 0.12 | 0.00 | 0.00 | hypothetical protein |
| GE005147 | *--* | -3.08 | 0.12 | 0.00 | 0.00 | hypothetical protein |
| GE005103 | *hxcR* | -3.25 | 0.11 | 0.00 | 0.00 | putative type II secretion system protein HxcR |
| GE002122 | *thlA* | -3.28 | 0.10 | 0.00 | 0.00 | Acetyl-CoA acetyltransferase |
| GE005091 | *--* | -3.29 | 0.10 | 0.00 | 0.00 | hypothetical protein |
| GE002123 | *ttuB* | -3.30 | 0.10 | 0.00 | 0.00 | Putative tartrate transporter |
| GE004804 | *padC* | -3.46 | 0.09 | 0.00 | 0.00 | putative phenolic acid decarboxylase |

Figure S1 RNA-Seq features. (A) PCA and Pearson correlation coefficient between HKE9, HKE9-M-*rpoS* and HKE9-C-M-*rpoS*; (B) Volcanic maps between HKE9 and HKE9-M-*rpoS*; (C) Volcanic maps between HKE9 and HKE9-C-M-*rpoS*; (D) Volcanic maps between HKE9-M-*rpoS* and HKE9-C-M-*rpoS*.


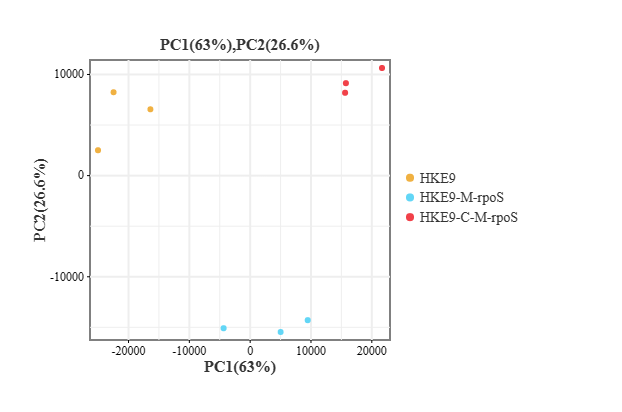
A
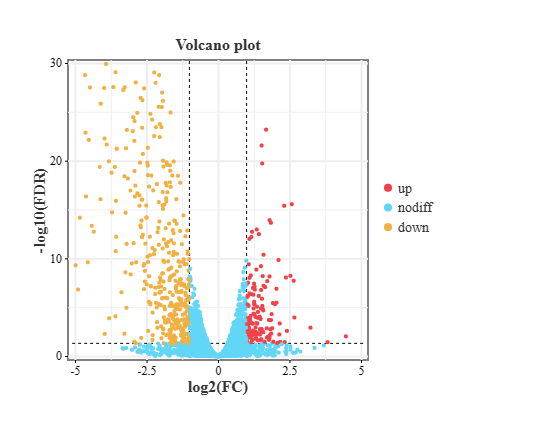
B


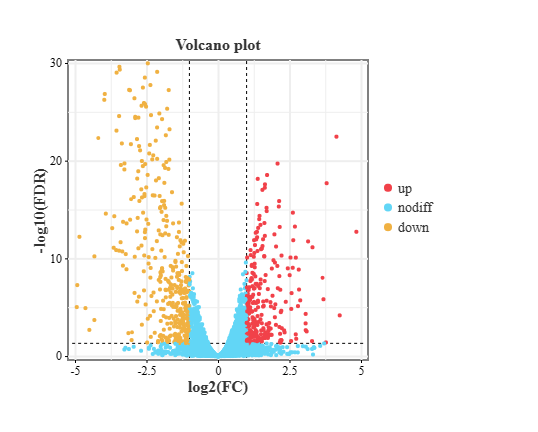
C
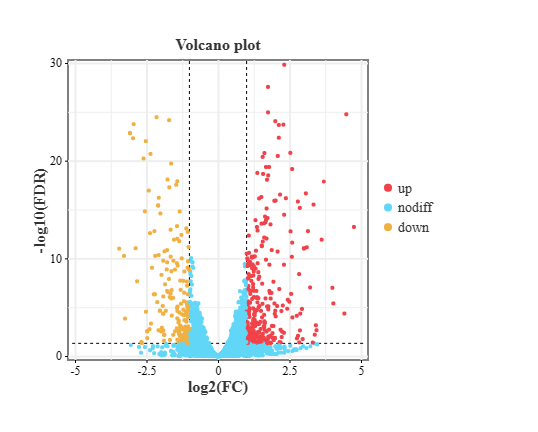
D

For Figure1A, the transcription of three replicates of HKE9, HKE9-M-*rpoS* and HKE9-C-M-*rpoS* had a significant inter groups difference; For the Volcanic maps of 1B, 1C, 1D, it showed the up-regulated (RED) and down-regulated (YELLOW) genes, the detail information of those genes was listed in TableS4, S5, S6, separately.

Figure S2 Validation of transcriptome results and quantitative RT-PCR. (A) HKE9 vs HKE9-M-*rpoS* RNA validation; (B) HKE9 vs HKE9-C-M- *rpoS* RNA validation.


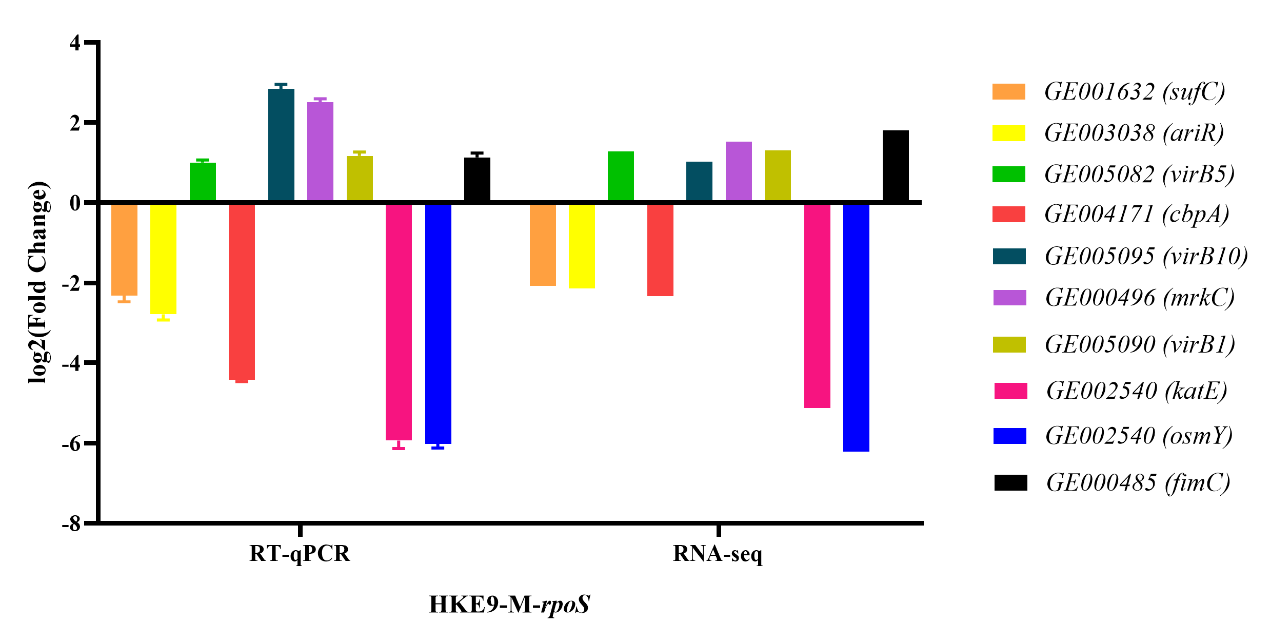
A


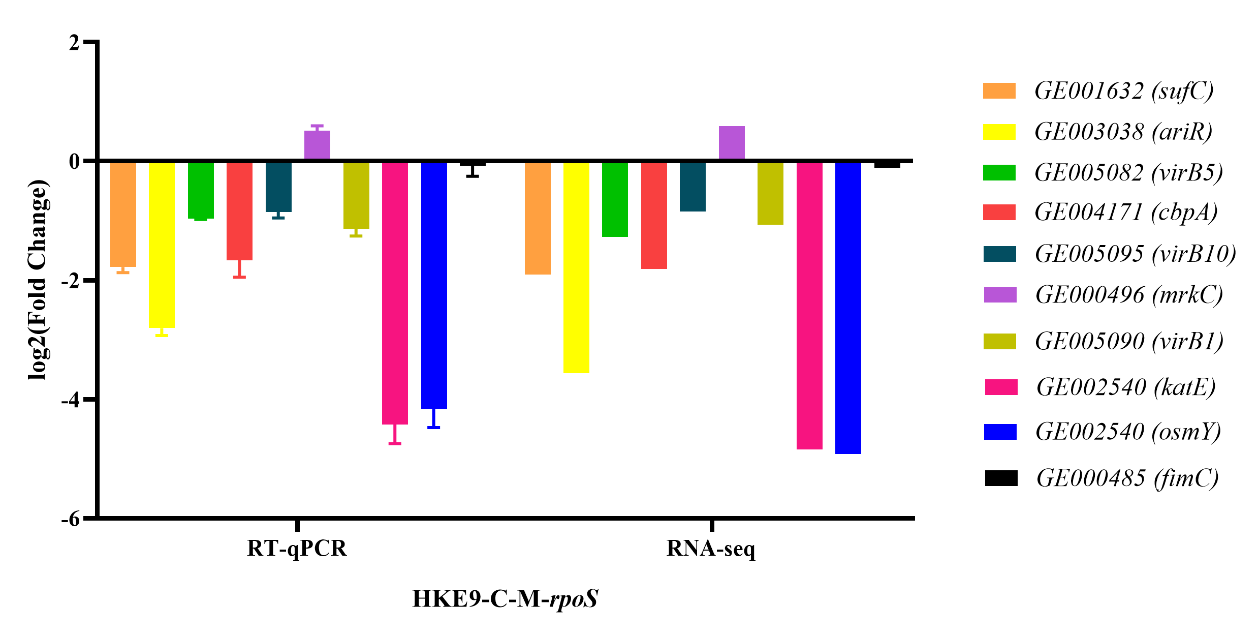
B


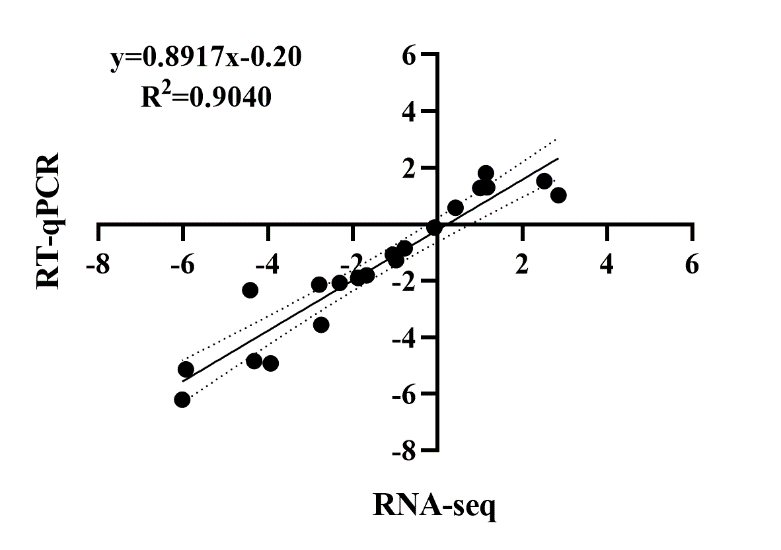
C
